# Upregulation of lysine‐specific demethylase 6B aggravates inflammatory pain through H3K27me3 demethylation‐dependent production of TNF‐α in the dorsal root ganglia and spinal dorsal horn in rats

**DOI:** 10.1111/cns.14281

**Published:** 2023-06-08

**Authors:** Yiming Qiao, Liren Li, Liying Bai, Yan Gao, Yin Yang, Li Wang, Xueli Wang, Zongyi Liang, Ji‐Tian Xu

**Affiliations:** ^1^ Department of Physiology and Neurobiology, School of Basic Medical Sciences Zhengzhou University Zhengzhou China; ^2^ Department of Anesthesiology, Pain and Perioperative Medicine, The First Affiliated Hospital Zhengzhou University Zhengzhou China; ^3^ Neuroscience Research Institute Zhengzhou University Zhengzhou China

**Keywords:** dorsal root ganglia, epigenetics, inflammatory pain, lysine‐specific demethylase 6B, spinal cord, TNF‐α

## Abstract

**Aims:**

Lysine‐specific demethylase 6B (KDM6B) serves as a key mediator of gene transcription. It regulates expression of proinflammatory cytokines and chemokines in variety of diseases. Herein, the role and the underlying mechanisms of KDM6B in inflammatory pain were studied.

**Methods:**

The inflammatory pain was conducted by intraplantar injection of complete Freund's adjuvant (CFA) in rats. Immunofluorescence, Western blotting, qRT‐PCR, and chromatin immunoprecipitation (ChIP)‐PCR were performed to investigate the underlying mechanisms.

**Results:**

CFA injection led to upregulation of KDM6B and decrease in the level of H3K27me3 in the dorsal root ganglia (DRG) and spinal dorsal horn. The mechanical allodynia and thermal hyperalgesia following CFA were alleviated by the treatment of intrathecal injection of GSK‐J4, and by microinjection of AAV‐EGFP‐KDM6B shRNA in the sciatic nerve or in lumbar 5 dorsal horn. The increased production of tumor necrosis factor‐α (TNF‐α) following CFA in the DRGs and dorsal horn was inhibited by these treatments. ChIP‐PCR showed that CFA‐induced increased binding of nuclear factor κB with TNF‐α promoter was repressed by the treatment of microinjection of AAV‐EGFP‐KDM6B shRNA.

**Conclusions:**

These results suggest that upregulated KDM6B via facilitating TNF‐α expression in the DRG and spinal dorsal horn aggravates inflammatory pain.

## Graphical abstract

The intraplantar CFA‐induced upregulation of KDM6B promoted binding of NF‐κB with TNF‐α promoter via inducing demethylation of H3k27me3. And this combination enhanced TNF‐α expression in the DRG and spinal dorsal horn and contributed to the pathogenesis of inflammatory pain following CFA injection.

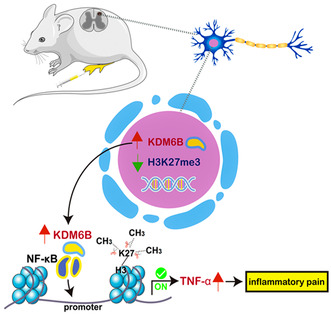

## 1 INTRODUCTION

Chronic inflammatory pain, which lasts for 6 weeks or longer, is mainly treated by nonsteroidal anti‐inflammatory drugs (NSAIDs) and opioids in clinics, and these methods are limited due to their side effects.^1^ Despite the number of studies have been performed, scientific understandings of the original mechanisms of inflammatory pain are still not fully understood.^2^ It has been well documented that proinflammatory mediators which released at sites of inflammation are capable of sensitizing pain‐sensing primary afferent neurons, and consequently, lead to a persistent increase in pain‐related synaptic transmission in the spinal dorsal horn.^3^ However, the regulation of these proinflammatory mediators' expression remains largely unclear.

Emerging evidence suggests that epigenetics play a fundamental role in regulating gene expression in response to environmental stimuli.^4^, ^5^, ^6^, ^7^, ^8^ Lysine‐specific demethylase 6B (KDM6B), with another name JMJD3, is a member of the JmjC histone demethylases family that specifically demethylates the trimethylated lysine at position 27 of H3 (H3K27me3) protein to regulate correlated gene expression.^9^ H3K27me3 is associated with a condensed chromatin conformation and exerts a repressive epigenetic mark.^10^ The upregulation of KDM6B demethylates H3K27me3 to H3K27me2 or H3K27me1, thus leading to the removal of the methylation mark from H3K27 and actives gene transcription.^11^, ^12^ Compelling evidence suggests that KDM6B is involved in the regulation of cell differentiation and inflammation by targeting distinct transcription factors which control cytokine gene expression.^10^, ^13^, ^14^ KDM6B depletion or inhibition decreases the expression of toll‐like receptor 4 and negates downstream NF‐κB proinflammatory signaling and subsequently ameliorates lipopolysaccharide‐induced inflammation in a mastitis model.^15^, ^16^, ^17^ Recent studies reveal that abnormal expression of KDM6B in the brain contributes to the pathogenesis of alcohol dependence,^18^ cocaine reward memory,^19^ and increased susceptibility to depression^20^ by epigenetic regulation of proinflammatory signaling pathway. Using lumbar 5 spinal nerve ligation (SNL), we found that upregulated KDM6B in the dorsal root ganglia and spinal dorsal horn contributes to the development and maintenance of neuropathic pain via facilitating the expression of IL‐6.^21^ Recently, Achuthan reported that granulocyte‐macrophage colony‐stimulating factor (GM‐CSF) via enhancing JMJD3 demethylase activity mediates arthritic pain.^22^ But the underlying mechanisms are incompletely understood. In the current study, the inflammatory pain was conducted by intraplantar injection of complete Freund's adjuvant (CFA) in male rats. The expression and the role of KDM6B in the pathogenesis of abnormal pain were investigated.

## 2 MATERIALS AND METHODS

### 2.1 Animals

Male Sprague–Dawley rats weighing 180–250 g were used. The rats were housed in separate cages with free access to food and water. The room temperature was kept at 23 ± 2°C under a 12:12‐h light–dark cycles. The animals were purchased from the Zhengzhou Laboratory Animal Center of Zhengzhou University in China. All animal experimental procedures were approved by the Animal Care and Use Committee of Zhengzhou University, China, and were carried out in accordance with the guidelines of the National Institutes of Health on animal care and the ethical guidelines for investigation of experimental pain in conscious animals.

### 2.2 Intraplantar injection of complete Freund's adjuvant for inflammatory pain

The inflammatory pain was conducted as described previously.^23^ After transient anesthesia, a single hypodermic injection of 100 μL complete Freund's adjuvant (CFA) was performed in the left plantar hind paw. For the control group, an equivalent volume of sterilized normal saline was injected in the same way in rats.

### 2.3 Pain‐related behavioral test

The behavioral tests were performed followed our formerly described methods.^24^ In brief, each animal was loosely adapted in a plastic box on a metal mesh for 3 consecutive days (15 min/day) before the baseline measurement. The paw withdrawal threshold (PWT) was identified to evaluate the mechanical sensitivity by treating the plantar surface of the hind paw with von Frey hairs, and 50% PWT was determined based on the up–down method.^25^ The evaluation of heat hypersensitivity was assessed by paw withdrawal latency (PWL) using a plantar analgesia tester (7370, Ugo Basile) according to the protocols published by Hargreaves et al.^26^ All the evaluators were blinded to the experimental design.

### 2.4 Intrathecal catheterization and drugs delivery

Drugs were delivered by intrathecal (i.t.) injection to rats. The intrathecal catheterization was performed as described previously.^27^ One week after the surgery, the rats were subjected to i.t. injection of GSK‐J4, a specific inhibitor of KDM6B, which was performed 30 min before intraplantar injection of CFA and once daily thereafter for 3 days. The doses of GSK‐J5 (5, 25, and 50 μg/10 μL, TOCRIS, 4594/10) used in the current experiment were based on a previous study.^21^

### 2.5 Intraspinal cord and intrasciatic nerve microinjection

To knockdown KDM6B only in the L5 spinal dorsal horn or in the DRG, the pAAV2‐CBG‐EGFP‐3xFLAG‐WPRE‐H1‐kdm6b shRNA or negative control pAAV2‐CBG‐EGFP‐3xFLAG‐WPREH1‐ncRNA (Obio Technology) was microinjected into unilateral L5 spinal dorsal horn or sciatic nerve following the methods described previously.^21^, ^28^ The viral solution (2 μL, titer >1.2 × 10^13^/mL) was injected at a rate of 100 nL/min with the micropipette connected to a Hamilton syringe. Four weeks later, the CFA injection in the plantar was carried out on the AAV transfected side.

### 2.6 Western blotting

Western blotting was performed according to our previously published procedures.^29^, ^30^ Briefly, after animals were sacrificed by decapitation, the L4/5 DRGs and L4‐6 spinal dorsal horn were harvested and homogenized with lysis buffer. After the protein concentrations were measured, the samples were subjected to electrophoresis and transferred onto PVDF membranes. The blotting membranes were blocked and incubated overnight with primary antibody. The proteins were detected with horseradish peroxidase‐conjugated secondary antibodies, visualized using the chemiluminescence reagents (Millipore P90719), and detected by a machine of ProteinSimple (FluorChem E). The intensities of blots were quantified by a computer‐assisted imaging analysis system (ImageJ, NIH). The following primary antibodies were used: KDM6B (1:1000, Invitrogen PA5 72751), TNF‐α (1:500, Invitrogen PA1 40281; 1:500 Abcam ab6671), NF‐κB p65 (1:1000, CST 4764s), NF‐κB p‐p65 (1:1000, CST 3033s), histone H3K27me3 (1:1000, Invitrogen P/N A15024), histone H3 (1:1000, Abcam ab4955), and β‐actin (1:10,000, Sigma A1978).

### 2.7 Immunofluorescence staining

Immunofluorescence staining was done following our previous methods.^29^, ^31^ After perfusing through ascending aorta with 4% paraformaldehyde, the L4/5 DRGs and L4‐6 spinal cord were removed and placed into 4% paraformaldehyde for postfixing overnight. Transverse DRG (16 μm) and spinal cord sections (25 μm) were cut on a cryostat and prepared for immunofluorescence staining with primary antibodies for KDM6B (1:200, Invitrogen PA5 72751) and TNF‐α (1:100, Invitrogen PA1 40281; 1:100 Abcam ab6671). For double immunofluorescence staining, the sections were incubated with a mixture of FITC‐(1:200, Jackson ImmunoResearch) and Cy3‐conjugated secondary antibody (1:400, Jackson ImmunoResearch) for 2 h at room temperature. All sections were examined by a high‐resolution laser confocal fluorescence microscope (Nikon A1R MP^+^). The ImageJ was used to quantify the intensity of immunofluorescence following the method described previously.^21^

### 2.8 RNA extraction and real‐time quantitative polymerase chain reaction (qPCR)

qPCR was performed following the method described previously.^24^, ^29^ After the rats were sacrificed by decapitation, the L4/5 DRGs and spinal dorsal horn were harvested. Total RNA was extracted via the Trizol method. Reverse transcription was performed using oligo‐dT primers and PrimeScriptII RTase (TAKARA) according to the manufacturer's protocol. Each sample was run in triplicate in a 20 μL reaction volume which contains 10 μM each of forward and reverse primers, 10 μL of SYBR Green qPCR Super Mix (Invitrogen), and 25 ng of cDNA. Reactions were performed in an Applied ABI QuantStudio 3 Real‐Time PCR System. The relative expression of KDM6B and TNF‐α mRNA was quantified through the 2^−ΔΔCT^ method. The rat‐specific primer sequences of KDM6B, TNF‐α, and GAPDH were listed in Table 1.

**TABLE 1:** Sequences (5′‐3′) of primers used.

Gene	Forward primer	Reverse primer
KDM6B	CCTGTTCGTGACAAGTGAGAAT	AGCTCCTCAGTGCGGTACT
TNF‐α	ATGGGCTCCCTCTCATCAGTTCC	CCTCCGCTTGGTGGTTTGCTAC
GAPDH	GACATGCCGCCTGGAGAAAC	AGCCCAGGATGCCCTTTAGT

### 2.9 Chromatin immunoprecipitation (ChIP) assays

ChIP assay was performed with a ChIP Assay Kit (Millipore, Catalog # 17‐295) following our described method previously.^21^, ^29^ In brief, the homogenized solution from L4‐5 DRGs or spinal dorsal horn was cross‐linked with 1% formaldehyde at 37°C for 10 min and terminated by the addition of 125 mM glycine. Sonication conditions to the lysed sample were tested to yield DNA fragments averaging 600–800 bp as assessed by agarose gel electrophoresis. After precleaned with protein G agarose, the samples were subjected to immunoprecipitation with 10 μg rabbit anti‐NF‐κB p‐p65 (Invitrogen) or normal rate IgG, which served as negative control, at 4°C overnight. Ten percent of the sample was used for immunoprecipitation as the input. The precipitated protein–DNA complexes were eluted and purified and subjected to PCR for amplification of the TNF‐α promoter fragments, and the protein was subjected to Western blotting to examine the level of H3K27me3 at the NF‐κB‐binding site. The binding sites of NF‐κB in the promoter region of TNF‐α were predicted from the PROMO (http://alggen.lsi.upc.es) and JASPAR databases (http://jaspar2016.genereg.net). The primers were designed to amplify NF‐κB p65‐bound DNA fragments (−1 to −1800 bp of the TNF‐α promoter region) and used to detect the TNF‐α promoter gene by ChIP‐PCR. All the primers were listed in Table 2.

**TABLE 2:** Primers to TNF‐α promoter region.

No	Forward primer	Reverse primer
P1	CTGGTGAGGACGGAGAGGAGATTC	GCTGTGTACCGGCTGCCTTTATAG
P2	TGCCTTCAGCCACTTCCTCT	ACCCTGGGAACTGAAACCCA
P3	CAGAGGGTGGGAGAGTGTCCAG	GGGCTTAGACTGCTGCTTTCGG
P4	TCTGCTTGTGTCTGTCTTGGATTGG	CTCCCAGAACCTCCGTCTCTCC
P5	AGAGCAGGGTATTTGTGGGTCTAGG	TCTGTGGTAACTGACGCCTTTGTG
P6	GAATCTAGGGAAACTCAGGAACTGG	CAGGAACTTCTAGACAGGCTTGAG

### 2.10 Statistical analysis

All data are presented as the means ± SD and analyzed with GraphPad Prism 8.2.1 (GraphPad). Sample sizes per group were predicted based on a power analysis (G*Power 3.0.10) and the experience of our previous studies.^21^, ^29^ All data were subjected to tests for normality by Shapiro–Wilk test. If the Data exhibit normal distribution, the differences were tested using Dunnett's multiple‐comparisons test or using Student's *t‐*test if only two groups were applied. Otherwise, if the data do not exhibit a normal distribution, it was analyzed via a nonparametric Kruskal–Wallis or Mann–Whitney *U*‐test. Data were considered significant when *p* < 0.05.

## 3 RESULTS

### 3.1 Intraplantar injection of CFA resulted in upregulation of KDM6B and reduction in the level of H3K27me3 in the DRG and spinal dorsal horn

**FIGURE 1:**
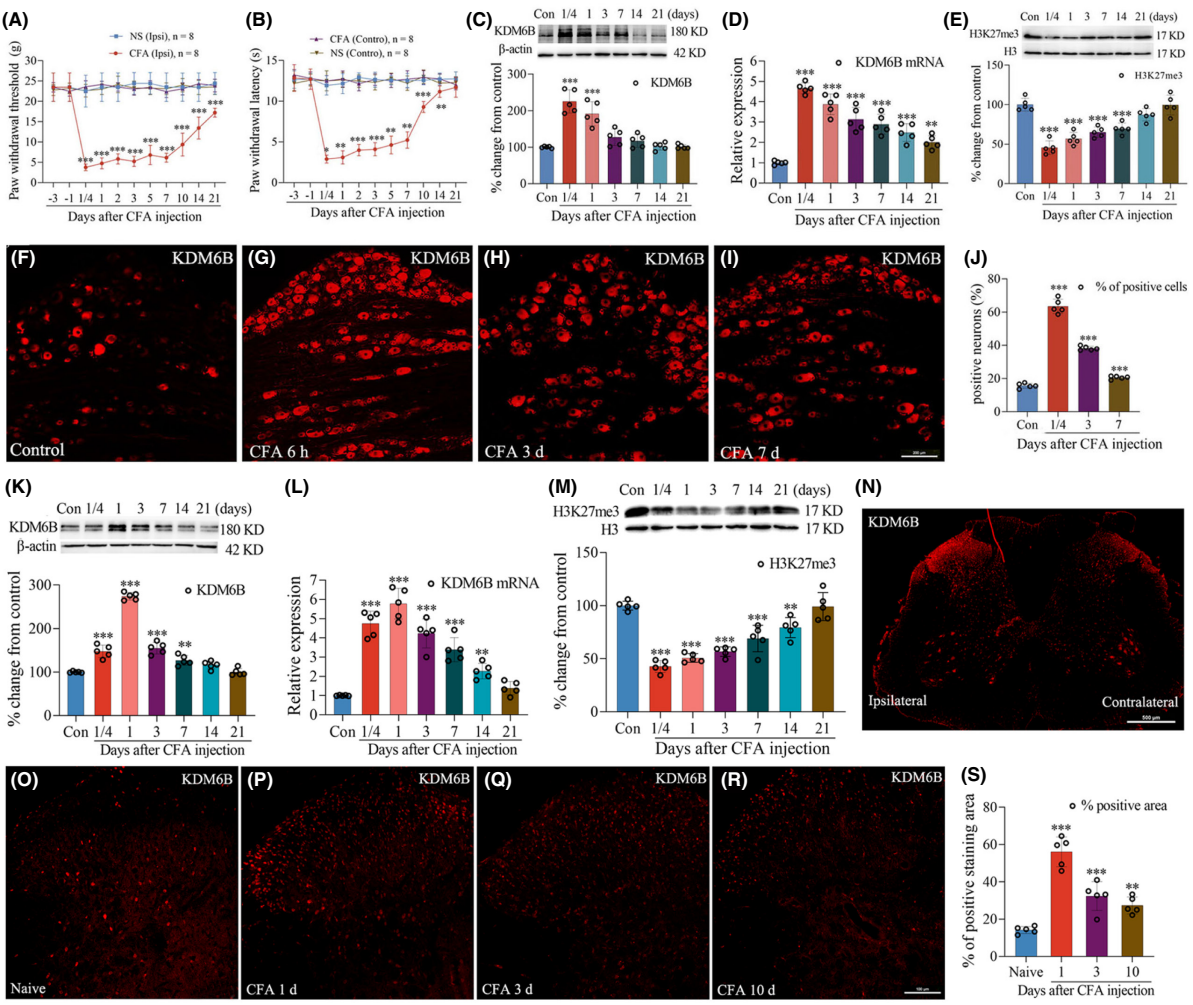
Intraplantar injection of CFA resulted in increase in the expression of KDM6B and decrease in the level of H3K27me3 in the L4/5 DRGs and spinal dorsal horn. (A, B) CFA injection led to reductions in PWT (A) and PWL (B) in ipsilateral hind paw. (C–E) CFA injection induced increase in the production of KDM6B protein (C) and mRNA (D), and decrease in the level of H3K27me3 (E). (F–I) Images of immunofluorescence staining showed increase in KDM6B positive‐staining cells after CFA injection. (J) Quantitative analysis revealed a significant increase in KDM6B positive‐staining cells after CFA injection. (K–M) CFA induced increase in the production of KDM6B protein (K) and mRNA (L), and decrease in the level of H3K27me3 (M) in the ipsilateral dorsal horn. (N) Representative images of immunofluorescence staining showed a clear increase in KDM6B positive‐staining immunoreactivity in the ipsilateral dorsal horn 1 day after CFA injection. (O–S) Quantitative analysis revealed a significant increase in KDM6B‐positive staining area in the dorsal horn after CFA injection. All data are presented as mean ± SD. *p < 0.05, **p < 0.01, ***p < 0.001 vs. control group or baseline. Scale bar: (F–I and O–R) = 100 μm, (N) = 500 μm.

Consistent with our previous study,^23^ intraplantar injection of CFA resulted in significant decrease in PWT (Figure 1A) and PWL (Figure 1B) in the ipsilateral hind paw. Correlating the change in pain‐related behavior, the Western blot and qPCR analysis showed that the CFA treatment significantly increased the production of KDM6B protein and the expression of KDM6B mRNA in the ipsilateral DRGs (**p < 0.01, ***p < 0.001 vs. control group, Figure 1C,D). Moreover, the CFA injection led to a significant reduction in H3K27me3 in the DRG (***p < 0.001 vs. control group, Figure 1E). Images of immunofluorescence staining showed an increase in KDM6B immunoreactivity in ipsilateral DRG (***p < 0.001 vs. control group, Figure 1F–J).

In addition, the CFA injection caused increases in KDM6B protein (Figure 1K) and mRNA (Figure 1L), and reduction in the level of H3K27me3 (Figure 1M) in the ipsilateral spinal dorsal horn. Compared with the control group, the significant increases in KDM6B protein and mRNA occurred at 6 h and lasted to day 7 after CFA injection. However, the reduction in H3K27me3 occurred at 6 hours and lasted to day 7 following CFA injection (**p < 0.01, ***p < 0.001 vs. control group, Figure 1K–M). Images of immunofluorescence staining showed a clear increased immunoreactivity of KDM6B protein in the ipsilateral dorsal horn than that of contralateral dorsal horn 1 day after CFA injection (Figure 1N). The quantitative data showed this increase started at 6 h and persistence over 10 days after CFA injection (**P < 0.01, ***P < 0.001 vs. naive group, Figure 1O–S). The Shapiro–Wilk test was used for normality, nonparametric Kruskal–Wallis test was used for difference in the results in Figure 1A,B, and Dunnett's multiple‐comparisons test was used for difference in the results in Figure 1C–E,J–M,S.

### 3.2 The cell type and the transition of KDM6B‐expressing cells in the DRG and spinal dorsal horn following CFA injection

**FIGURE 2:**
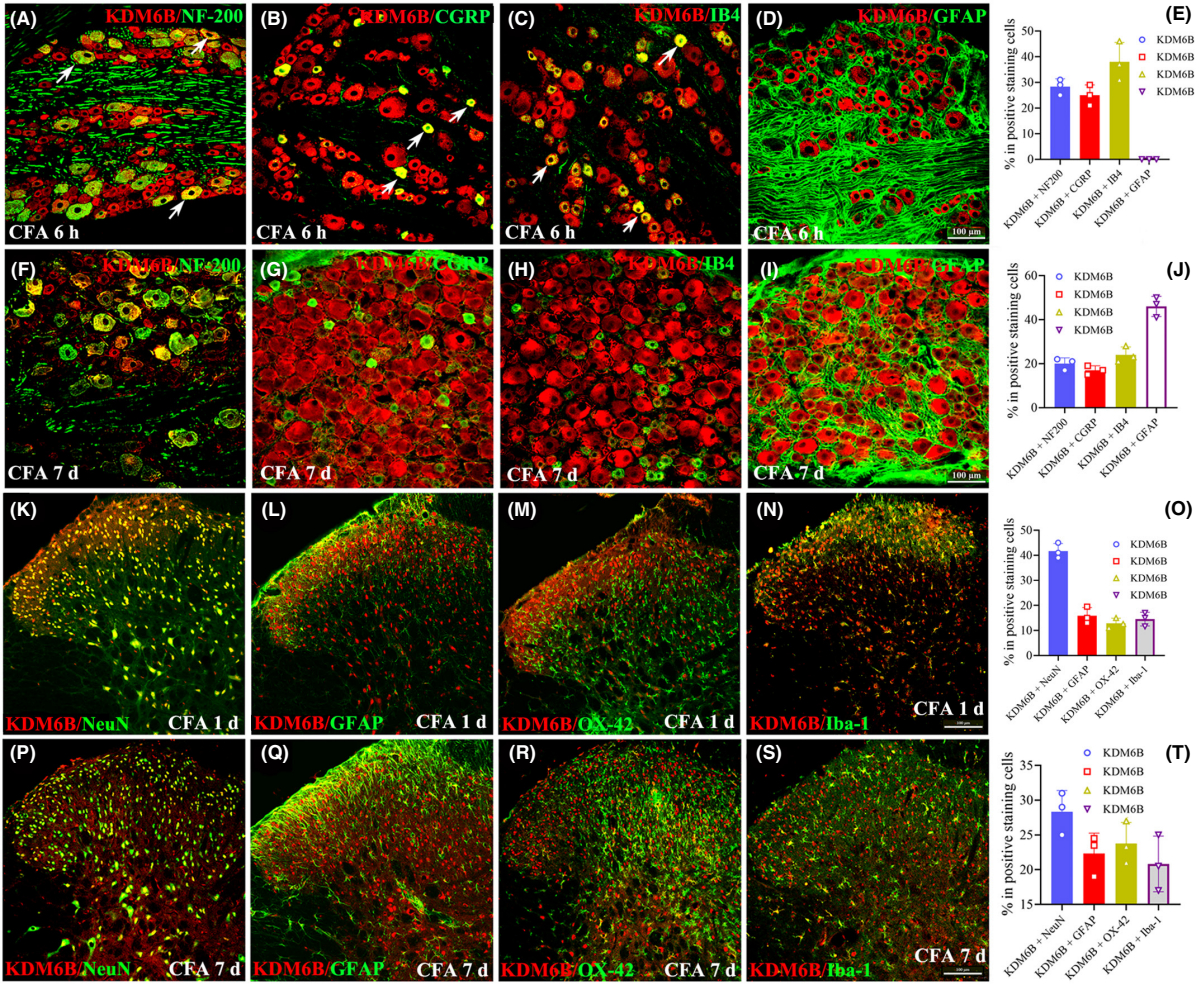
The cell types expressing KDM6B in the DRG and spinal dorsal horn following CFA injection. (A–E) Representative images of double immunofluorescence staining in the ipsilateral L5 DRG between KDM6B and NF‐200, CGRP, IB4, and GFAP showing colocalization of KDM6B with NF‐200 (A), CGRP (B), and IB4 (C), but not with GFAP (D) at 6 h after CFA injection (E). (F–J) Images showing that KDM6B colocalized not only with NF‐200 (F), CGRP (G), and IB4 (H) but also with GFAP (I) marked cells in the DRG on day 7 after CFA injection (J). (K–O) Representative images showing colocalization between KDM6B and NeuN (K), GFAP (L), OX‐42 (M), and Iba‐1 (N) in the dorsal horn on day 1 after CFA injection (O). (P–T) Images showing colocalization between KDM6B and NeuN (P), GFAP (Q), OX‐42 (R), and Iba‐1 (S) in the dorsal horn on day 7 after CFA injection (T). Scale bar = 100 μm.

To identify the cell types that expressed KDM6B after CFA treatment, we performed double immunofluorescence staining of KDM6B with four cell‐specific markers of DRG: NF‐200 (myelinated A‐fiber, Figure 2A), CGRP (unmyelinated peptidergic C‐fiber, Figure 2B), IB4 (unmyelinated nonpeptidergic C‐fiber, Figure 2C), and GFAP (satellite glial cells, Figure 2D). The results showed that KDM6B was primarily colocalized with NF‐200, CGRP, and IB4, but not with GFAP at 6 h after CFA injection (Figure 2A–E). However, except NF‐200‐ (Figure 2F), CGRP‐ (Figure 2G), and IB4 (Figure 2H)‐marked cells, a clear colocalization of KDM6B with GFAP (Figure 2I,J) was also detected on day 7 after CFA injection. In the dorsal horn, the double‐immunofluorescence staining was carried out between KDM6B and NeuN (a marker of the neuronal cell), GFAP (a marker of astrocyte), OX‐42 (a marker of microglia), and Iba‐1 (a marker of activated and inactivated microglia), respectively. The results showed that the KDM6B colocalized with NeuN (Figure 2K), GFAP (Figure 2L), OX‐42 (Figure 2M), and Iba‐1 (Figure 2N) on day 1 after CFA injection. However, an in increased colocalization of KDM6B with GFAP (Figure 2Q), OX‐42 (Figure 2R), and Iba‐1 (Figure 2S) was detected on day 7 after CFA injection (Figure 2T).

### 3.3 Intrathecal injection of GSK‐J4 alleviated mechanical allodynia and thermal hyperalgesia and prevented increase in the expression of TNF‐α in the DRG and spinal dorsal horn following CFA injection

**FIGURE 3:**
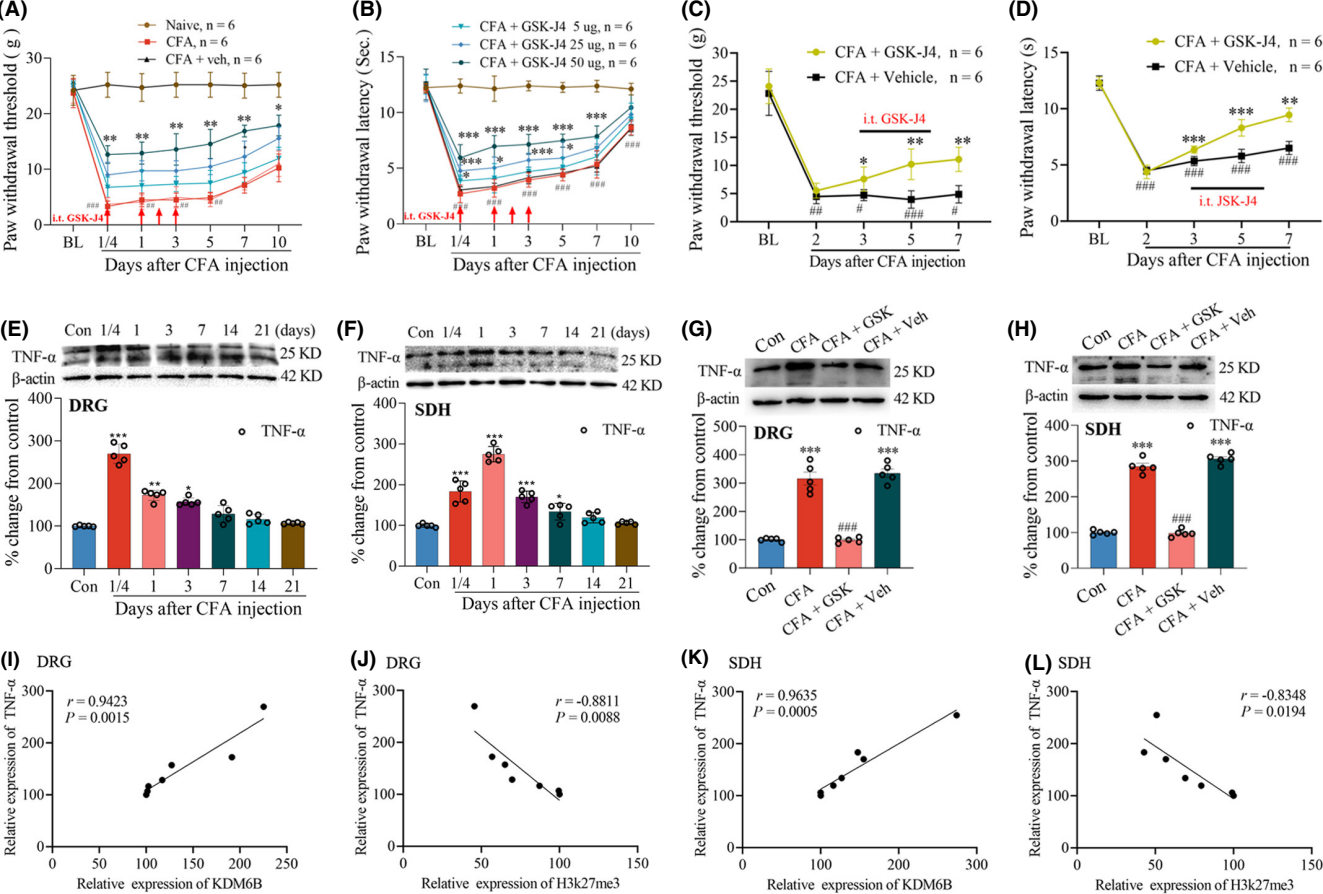
Repeat intrathecal (i.t.) injections of GSK‐J4 impaired inflammatory pain and suppressed the increase in the production of TNF‐α in the DRG and spinal dorsal horn following CFA injection. (A, B) Prior i.t. injection of GSK‐J4 dose dependently prevented the reduction in PWT (A) and PWL (B) after CFA injection. (C, D) The established mechanical allodynia (C) and thermal hyperalgesia (D) were alleviated by the treatment of GSK‐J4. (E, F) Intraplantar injection of CFA led to increase in the production of TNF‐α in the DRG (E) and spinal dorsal horn (F). (G, H) The CFA‐induced increase in the production of TNF‐α in the DRG (G), and dorsal horn (H) was inhibited by the treatment of GSK‐J4. (I–L) Results of correlation analysis between the production of KDM6B and TNF‐α, and between H3K27me3 and TNF‐α in the DRG and dorsal horn following CFA injection. *p < 0.05, **p < 0.01, ***p < 0.001; ^#^ *p <* 0.05, ^##^ *p <* 0.01, ^###^ *p <* 0.001. BL, baseline; Con, control; SDH, spinal dorsal horn; Veh, vehicle.

It has been reported that GSK‐J4 worked as a specific inhibitor of KDM6B in the treatment of inflammatory diseases.^32^, ^33^ In the current study, the repeat intrathecal (i.t.) injections of GSK‐J4 at doses of 5, 25, and 50 μg were performed to examine the role of KDM6B in the CFA‐induced inflammatory pain. Results of behavioral tests showed that GSK‐J4 treatment prevented the reductions in PWT and PWL after CFA injection. Compared to CFA plus vehicle (Veh: i.t. normal saline) group, the i.t. injection of GSK‐J4 resulted in significant increases in PWT (Figure 3A) and PWL (Figure 3B) in a dose‐dependent manner (*p < 0.05, **p < 0.01, and ***p < 0.001 vs. CFA or CFA + Vehicle group; ^##^ *p* < 0.01, ^###^ *P* < 0.001 vs. baseline). To further observe the role of KDM6B in the established inflammatory pain, the repeat i.t. injections of GSK‐J4 (50 μg/10 μL) were started on day 3 after CFA treatment. The results showed that CFA‐induced reductions in PWT (Figure 3C) and PWL (Figure 3D) were partially reversed by the treatment of GSK‐J4 (*p < 0.05, **p < 0.01, ***p < 0.001 vs. CFA + Vehicle group; ^#^ *p* < 0.05, ^##^ *p* < 0.01, ^###^ *p* < 0.001 vs. baseline). Compelling evidence has shown the TNF‐α plays critical role in the development of inflammatory diseases and pain. In the current study, we detected a significant increased expression of TNF‐α, which peaked at 6 h and lasted to day 3 in the DRG (Figure 3E). In the dorsal horn (Figure 3F), the increased production of TNF‐α occurred at 6 h, peaked on day 1, and persisted to day 7 following CFA injection (*p < 0.05, **p < 0.01, ***p < 0.001 vs. control group). However, these increases in TNF‐α were repressed in both the DRG (Figure 3G) and dorsal horn (Figure 3H) following the treatment of GSK‐J4 (***p < 0.001 vs. control (Con: intraplantar injection of saline) group; ^###^ *p* < 0.001 vs. CFA or CFA + Vehicle). To verify the relationship between KDM6B and TNF‐α, the correlation analysis was performed by Pearson *r*‐test. The results showed that there was a positive correlation between the production of KDM6B and TNF‐α in both the DRG (*r* = 0.9423; *p* = 0.0015, Figure 3I) and spinal dorsal horn (*r* = 0.9635; *p* = 0.0005, Figure 3K) following CFA injection. However, a negative correlation existed between the production of H3K27me3 and TNF‐α in both the DRG (*r* = −0.8811; *p* = 0.0088, Figure 3J) and spinal dorsal horn (*r* = −0.8348; *p* = 0.0194, Figure 3L). It implies that the CFA‐induced TNF‐α expression may depend on KDM6B activity. The Shapiro–Wilk test was used for normality, nonparametric Kruskal–Wallis test was used for difference in the results in Figure 3A,B,E,F, Mann–Whitney *U*‐test was used in Figure 3C,D, and Dunnett's multiple‐comparisons test was used in Figure 3G,H.

### 3.4 Knockdown KDM6B in the spinal dorsal horn and DRG attenuated the inflammatory pain and suppressed the TNF‐α production following CFA injection

**FIGURE 4:**
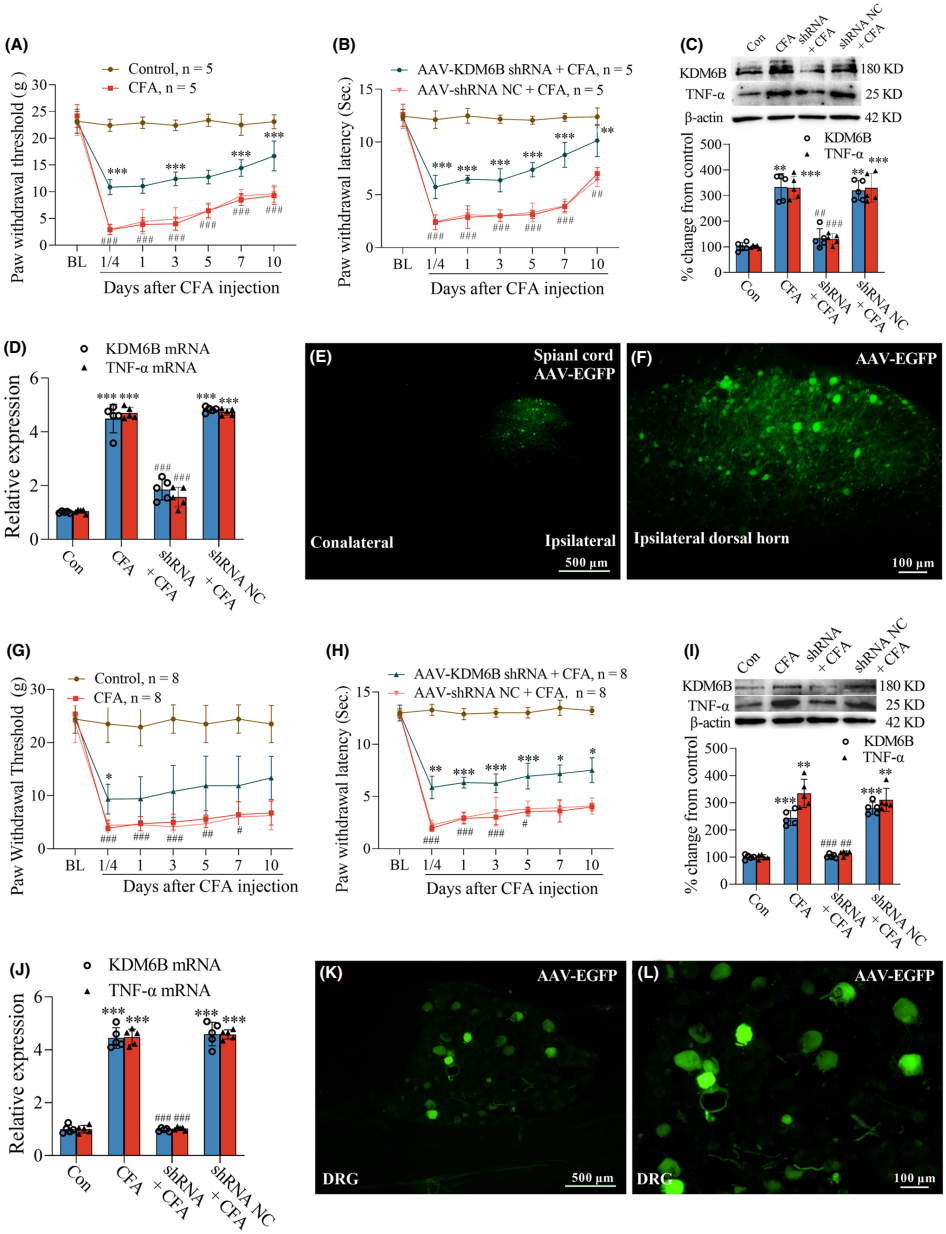
Microinjection of AAV‐EGFP‐KDM6B shRNA in the L5 spinal dorsal horn or sciatic nerve alleviated the inflammatory pain and reduced the TNF‐α production in the dorsal horn or DRGs following CFA injection. (A, B) L5 spinal cord injection of AAV‐EGFP‐KDM6B shRNA prevented the decrease in PWT (A) and PWL (B) following CFA injection. (C, D) The CFA‐induced increase in the production of KDM6B and TNF‐α protein (C) and mRNA (D) in the dorsal horn was inhibited by L5 spinal cord injection of AAV‐EGFP‐KDM6B shRNA. (E, F) Images from the spinal cord of injection of AAV‐EGFP‐KDM6B shRNA showing clear EGFP fluorescence in the ipsilateral (E and F), but not contralateral (E) dorsal horn. (G, H) Microinjection of AAV‐EGFP‐KDM6B shRNA in the sciatic nerve partially prevented the CFA‐induced reduction in PWT (G) and PWL (H). (I, J) The CFA‐induced increase in the production of KDM6B and TNF‐α protein (I) and mRNA (J) in the L4/5 DRGs was inhibited by the treatment of injection of AAV‐EGFP‐KDM6B shRNA in sciatic nerve. (K, L) Images from the L5 DRG of microinjection of AAV‐EGFP‐KDM6B shRNA in sciatic nerve showing clear EGFP fluorescence. *p < 0.05, **p < 0.01, ***p < 0.001; ^#^ *p <* 0.05, ^##^ *p <* 0.01, ^###^ *p <* 0.001. Scale bar: (E, K) = 500 μm and (F, L) = 100 μm.

To further verify the role of KDM6B in the pathogenesis of inflammatory pain, the microinjection of AAV‐EGFP KDM6B shRNA in the L5 spinal dorsal horn was performed. Compared with CFA or AAV‐EGFP‐shRNA NC (negative control, NC) plus CFA group, intraspinal cord injection of AAV‐EGFP‐KDM6B shRNA resulted in a significant increase in PWT (Figure 4A) and PWL (Figure 4B) following CFA injection (**p < 0.01, ***p < 0.001; ^##^ *p* < 0.01, ^###^ *p* < 0.001 vs. baseline). Results of Western blot (Figure 4C) and qPCR (Figure 4D) showed that the treatment of AAV‐EGFP‐KDM6B shRNA reduced the production of KDM6B protein and mRNA, and prevented the increased expression of TNF‐α (Figure 4C,D) in the dorsal horn (**p < 0.01, ***p < 0.001 vs. control group; ^##^ *p* < 0.01, ^###^ *p* < 0.001 vs. CFA group or AAV‐EGFP‐shRNA NC + CFA group). The L4‐5 spinal sections from intraspinal cord injection of AAV2‐EGFP‐KDM6B shRNA displayed clearly green fluorescence of EGFP in ipsilateral, but not contralateral, dorsal horn (Figure 4E,F).

To verify the role of DRG‐expressing KDM6B in the development of inflammatory pain, the intrasciatic nerve injection of AAV‐EGFP‐KDM6B shRNA was performed 4 weeks before CFA injection. The behavioral tests showed a significant increase in PWT (Figure 4G) and PWL (Figure 4H) in the group of AAV‐EGFP‐KDM6B shRNA plus CFA, but not in AAV‐EGFP‐shRNA NC plus CFA, when compared with CFA injection alone (*p < 0.05, **p < 0.01, ***p < 0.001; ^#^ *p* < 0.05, ^##^ *p* < 0.01, ^###^ *p* < 0.001 vs. baseline). The expression of KDM6B and TNF‐α protein (Figure 4I) and mRNA (Figure 4J) in the L4/5 DRGs were increased in the treatments of CFA or AAV‐EGFP‐shRNA NC plus CFA. But these increases were inhibited in the group of AAV‐EGFP‐KDM6B shRNA plus CFA (Figure 4I,J) (**p < 0.01, ***p < 0.001 vs. control group; ^##^ *p* < 0.01, ^###^ *p* < 0.001 vs. CFA group or AAV‐EGFP‐shRNA NC + CFA group). The L5 DRG sections from intrasciatic nerve injection of AAV‐EGFP‐KDM6B shRNA showed distinct green fluorescence of EGFP (Figure 4K,L). The Shapiro–Wilk test was used for normality, nonparametric Kruskal–Wallis test was used for difference in the results in Figure 4A,B,G,H, and Dunnett's multiple‐comparisons test was used in Figure 4C,D,I,J.

### 3.5 The cell‐type of TNF‐α‐expressing cells in the DRG and spinal dorsal horn following CFA injection

**FIGURE 5:**
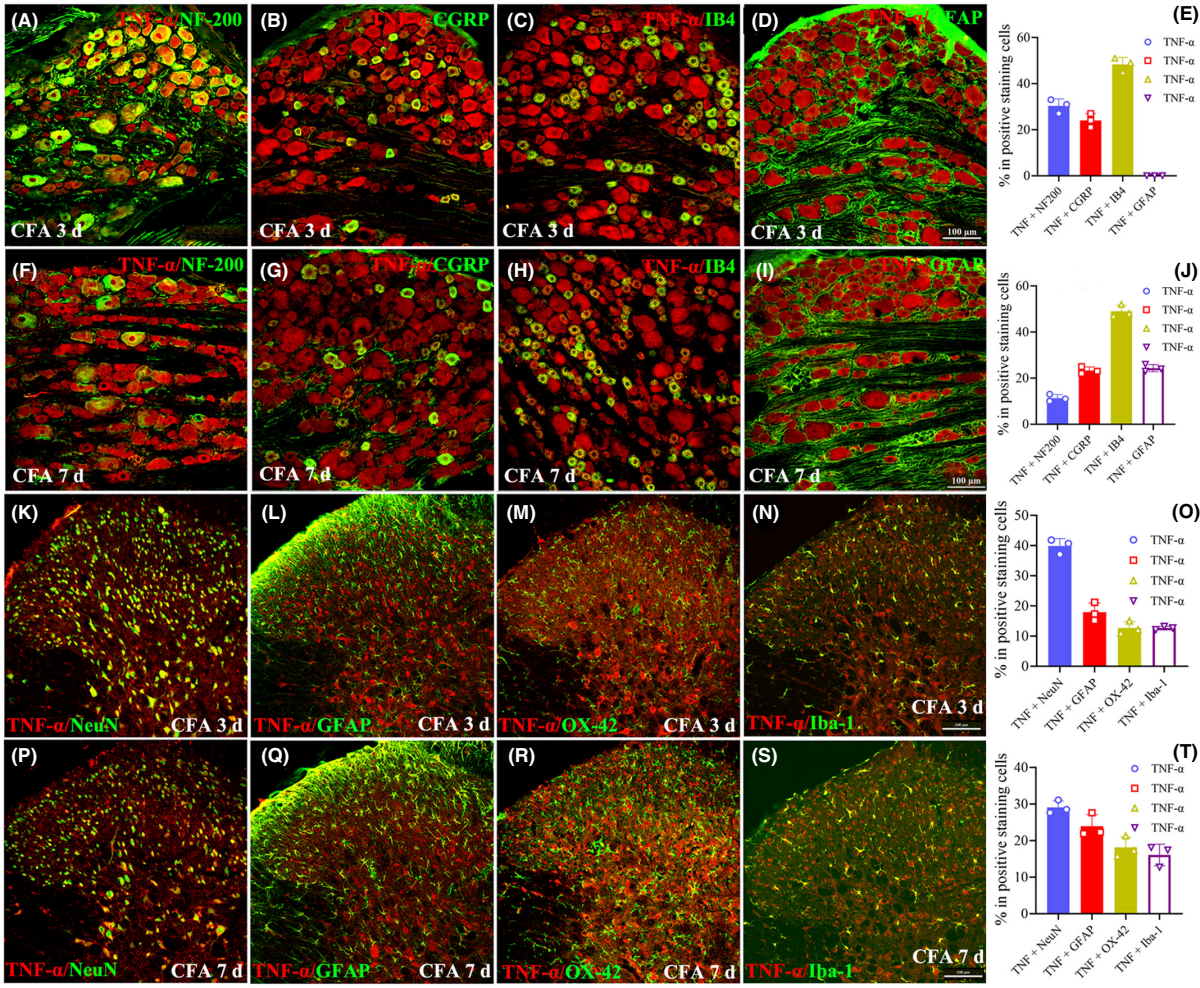
The cell type and transition of TNF‐α‐expressing cells in the DRG and spinal dorsal horn following CFA injection. (A–E) The representative images showing that the TNF‐α exclusively expressed in NF‐200‐ (A), CGRP‐ (B), and IB4‐ (C) marked neurons, but not in GFAP‐marked satellite glial cells (D and E) in the DRG on day 3 after CFA injection. (F–J) Except for NF‐200‐ (F), CGRP‐ (G), and IB4‐ (H) marked neurons, an increased expression of TNF‐α in GFAP‐marked satellite glial cells (I) was observed on day 7 after CFA injection (J). (K–O) The representative images showing a clear expression of TNF‐α in NeuN‐ (K), GFAP‐ (L), OX‐42‐ (M), and Iba‐1‐marked cells (N) in the dorsal horn on day 3 after CFA injection (O). (P–T) Compared to day 3, the TNF‐α in NeuN‐marked neuronal cells was decreased (P), and in GFAP‐marked astrocytes (Q) and OX‐42 (R) and Iba‐1 (S)‐marked microglial were increased in the dorsal horn on day 7 after CFA injection (T). Scale bar: A–D, F–I, K–N, P–S = 100 μm.

To observe the TNF‐α‐expressing cells in the DRG and spinal dorsal horn, the double immunofluorescence was performed. The results showed that the TNF‐α expressed (Figure 5A–E), exclusively, in neurons in the DRG on day 3 after CFA. But the satellite glial cells also expressed TNF‐α (Figure 5F–J) in the DRG on day 7 after CFA. In the dorsal horn, the TNF‐α expressed in neurons, astrocytes, and microglia on day 3 after CFA (Figure 5K–O), but an increased expression of TNF‐α in astrocytes and microglia was observed on day 7 after CFA injection (Figure 5P–T). The above results showed that TNF‐α expressed in the same cells at almost same time point as KDM6B in both the DRG and dorsal horn.

### 3.6 The binding of NF‐κB with TNF‐α promoter in the DRG and spinal dorsal horn depended on the demethylated activity of KDM6B following CFA injection

**FIGURE 6:**
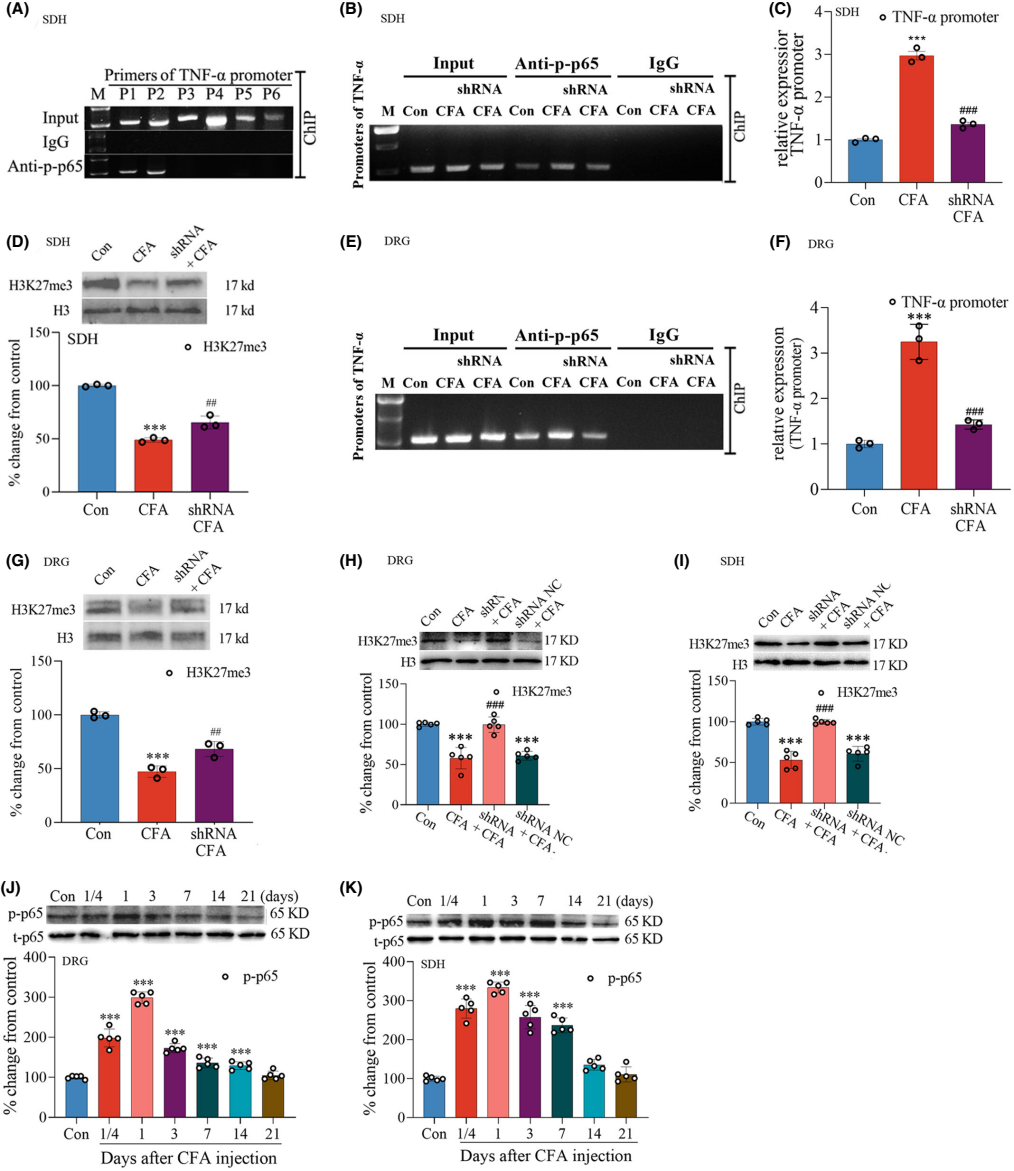
Microinjection of AAV‐EGFP‐KDM6B shRNA in the L5 spinal dorsal horn and sciatic nerve repressed the combination of NF‐κB with TNF‐α promoters following CFA injection. (A) Image of ChIP‐PCR showing primers P1 and P2 to TNF‐α promoter gene produced their predicted products when the amplified reaction was performed on the anti‐NF‐κB p‐p65‐linked DNA fragments. (B, C) Results of ChIP‐PCR showing that CFA‐induced increase in the combination of NF‐κB with TNF‐α promoter fragments in the dorsal horn was reversed by the treatment of microinjection of AAV‐EGFP‐KDM6B shRNA in the L5 spinal dorsal horn. (D) CFA‐induced reduction in H3K27me3 in NF‐κB p‐p65‐binding site in the dorsal horn was reversed by the treatments of microinjection of AAV‐EGFP‐KDM6B shRNA. (E, F) Results of ChIP‐PCR showing that the CFA‐induced increase in the binding of NF‐κB p65 with TNF‐α promoter in the DRG was inhibited by the treatment of intrasciatic nerve injection of AAV‐EGFP‐KDM6B shRNA. (G) The CFA‐induced reduction in H3K27me3 in NF‐κB p‐p65‐binding site in the DRG was reversed by the treatment of microinjection of AAV‐EGFP‐KDM6B shRNA in the sciatic nerve. (H, I) The CFA‐induced decrease in H3K27me3 in the DRG (H) and dorsal horn (I) was partially reversed by AAV‐EGFP‐KDM6B shRNA. (J, K) CFA injection led to significant increase in the level of NF‐κB p‐p65 in the L4/5 DRGs (J) and spinal dorsal horn (K). ***p < 0.001; ^##^ *p <* 0.01, ^###^ *p <* 0.001. SDH: spinal dorsal horn.

Previous study has shown that the LPS‐induced increased expression of TNF‐α is inhibited by the treatment of GSK‐J4.^34^, ^35^ In the current study, the ChIP‐PCR was used to examine the binding of NF‐κB at the promoter region of TNF‐α. The results showed that primers P1 (to TNF‐α promoter sequence: forward: CTGGTGAGGACGGAGA GGAGATTC; reverse: GCTGTGTACCGGCTGCCTTTATAG) and P2 (to TNF‐α promoter sequence: forward: TGCCTTCAGCCACTTCCTCT; reverse: ACCCTGGG AACTGAAACCCA), but not p3, p4, p5, and p6, produced the predicted products when the amplified reaction was performed on the anti‐NF‐κB p‐p65‐linked DNA fragments (Figure 6A). Therefore, the primer P2 (forward: TGCCTTCAGCCACTTCCTCT; reverse: ACCCTGGGAACTGAAACCCA) was used in the following ChIP‐PCR. The results showed that CFA injection resulted in a clear increase in TNF‐α promoter fragments, which were precipitated by anti‐NF‐κB p‐p65, compared to that of the control group. But this increase was reversed by the treatment of intraspinal cord injection of AAV‐EGFP‐KDM6B shRNA (Figure 6B). Results of qPCR also showed a significantly increased TNF‐α promoter DNA fragments precipitated by anti‐NF‐κB p‐p65, and this increase was reduced in the group of AAV‐EGFP‐KDM6B shRNA plus CFA (***p < 0.001 vs. control group; ^###^ *p* < 0.001 vs. CFA group, Figure 6C). Moreover, the level of H3K27me3 in the binding site of NF‐κB p65 was reduced in the group of CFA. But this reduction was reversed by the treatments of AAV‐EGFP‐KDM6B shRNA plus CFA (***p < 0.01 vs. control group; ^##^ *p* < 0.01 vs. CFA group, Figure 6D). The same results were also observed in the DRG in which the CFA‐induced increase in the binding of NF‐κB p65 with TNF‐α promoter was inhibited by the treatment of intrasciatic nerve injection of AAV‐EGFP‐KDM6B shRNA (***p < 0.001 vs. control group; ^###^ *p* < 0.001 vs. CFA group, Figure 6E,F), and the reduction in H3K27me3 in NF‐κB p65‐binding site was reversed by the treatment of AAV‐EGFP‐KDM6B shRNA (***p < 0.01 vs. control group; ^##^ *p* < 0.01 vs. CFA group, Figure 6G). The total level of H3K27me3, which was decreased by CFA injection in both the DRG and dorsal horn, was reversed by the treatment of AAV‐EGFP‐KDM6B shRNA injection (***p < 0.001 vs. control group; ^###^ *p* < 0.001 vs. CFA group, Figure 5H,I). The CFA injection also caused a remarkable increase in the level of NF‐κB p‐p65 in both the DRG (Figure 6J) and dorsal horn (Figure 6K) (***p < 0.001 vs. control group, Figure 6J,K). The Shapiro–Wilk test was used for normality, and Dunnett's multiple‐comparisons test was used in Figure 6C,D,F–K.

## 4 DISCUSSION

Previous studies have implicated a critical role of KDM6B in the pathogenesis of inflammatory diseases.^13^ Herein, we demonstrated that KDM6B was involved in the development of inflammatory pain. Intraplantar injection of CFA resulted in significant increase in the production of KDM6B and decrease in the level of H3K27me3 in both the DRG and spinal dorsal horn. The CFA‐induced mechanical allodynia and thermal hyperalgesia were alleviated by i.t. injection of GSK‐J4 or by microinjection of AAV‐EGFP‐KDM6B shRNA in both the sciatic nerve and L5 spinal dorsal horn. Mechanistically, the upregulated KDM6B promoted the binding of NF‐κB with TNF‐α promoter via inducing demethylation of H3K27me3. And this combination enhanced TNF‐α expression in the DRG and dorsal horn following CFA injection. These observations provided new evidence for further elucidating the mechanism of inflammatory pain.

Although the importance of epigenetically regulated gene expression in the induction and maintenance of inflammatory diseases has been well documented in the past decade,^36^, ^37^ the role of some enzymes involved in epigenetics in the pathogenesis of chronic pain still needs to be verified.^38^ H3K27me3 is a critical epigenetic event frequently associated with gene repression. KDM6B is a member of JmjC histone demethylases family that specifically demethylate the trimethylate lysine at position 27 of H3 protein to regulate correlated gene expression^39^, ^40^ and involves in the proinflammatory gene production.^13^, ^17^, ^22^, ^35^, ^41^ In our current study, we found that intraplantar injection of CFA led to increase in the expression of KDM6B and a reduction in the level of H3K27me3 in both the DRG and spinal dorsal horn. It has been demonstrated that the demethylation of H3K27me3 is one of the key steps to trigger gene expression via chromatin remodeling.^42^ The establishment of chronic pain, especially the peripheral sensitization in the DRG^43^ and central sensitization in the spinal dorsal horn,^44^ needs variety of de novo proteins to be produced. Therefore, we speculated that the upregulated KDM6B might be involved in the genesis of inflammatory pain via triggering gene expression. Our pain‐related behavioral tests showed a significant alleviative mechanical allodynia and thermal hyperalgesia following repeat i.t. injections of GSK‐J4. To discriminate the different functions of upregulated KDM6B in the DRG and spinal dorsal horn during the development of inflammatory pain, the microinjections of AAV‐EGFP‐KDM6B shRNA in both the sciatic nerve and L5 spinal dorsal horn were performed, separately. The results showed that the CFA‐induced abnormal pain was attenuated by the treatment of specific knockdown KDM6B in both the DRG and dorsal horn. These results indicate CFA‐induced upregulation of KDM6B in the DRG, and dorsal horn is required in the pathogenesis of inflammatory pain. Recently, Achuthan and colleagues reported that GM‐CSF via enhancing JMJD3 activity mediates arthritis. Treatment with GSK‐J4 reduces the production of CCL17 and alleviates arthritic pain.^22^ These findings agree with our present study.

Compelling evidence has highlighted the proinflammatory role of KDM6B in various inflammatory diseases by regulating the expression of several NF‐κB‐dependent cytokines.^13^, ^35^ The TNF‐α has been demonstrated as a potent mediator in the pathogenesis of variety of chronic pain.^45^, ^46^, ^47^, ^48^ In the current study, we detected an increased production of TNF‐α in both the DRG and spinal dorsal horn following CFA injection. But this increase was suppressed by the treatments of GSK‐J4 and AAV‐EGFP‐KDM6B shRNA. Previous studies have shown that peripheral nerve injury‐ and inflammation‐induced TNF‐α expression distributes not only in glial but also in neuronal cells in the DRG and dorsal horn.^49^ In the present study, we found that TNF‐α expressed in the same cells at almost the same time point as KDM6B in both the DRG and dorsal horn. The early increase in KDM6B and TNF‐α mainly comes from neurons, and the glial cells contributed to a delayed increase in KDM6B and TNF‐α in both the DRG and dorsal horn following CFA injection. These results indicate that CFA‐induced TNF‐α expression depends on the regulation of KDM6B. Recently, Davis reported that the increased JMJD3 in aortic tissues reduces the repressive H3K27 trimethylation at NF‐κB‐binding sites on inflammatory gene promoters, and inhibition of JMJD3 via GSK‐J4 reduces inflammatory cytokine expression.^41^ Our current results showed that CFA injection induced an increased binding of NF‐κB p65 with TNF‐α promoter, and this increase accompanied a significant reduction in the level of H3K27me3 in the area of TNF‐α promoter in both the DRG and spinal dorsal horn. It indicates that the CFA‐induced upregulation of KDM6B might through mediating H3K27me3 demethylation at TNF‐α promoter facilitates the binding of NF‐κB with TNF‐α promoter, and subsequently promotes expression of TNF‐α in the dorsal horn and DRG.

Obviously, the TNF‐α expression is not a unique factor of KDM6B‐mediated inflammatory pain in the present study. It has been reported that KDM6B‐regulated demethylation of H3K27me3 works as a transcriptional coactivator in several gene expression programs.^39^, ^50^, ^51^ Therefore, we speculate that upregulated KDM6B at present study may not only promote NF‐κB‐mediated TNF‐α expression but also regulate other signaling pathway, and may contribute together to the CFA‐induced inflammatory pain. Whether the other cytokines, chemokines, ion channels, and receptors were regulated by KDM6B and were involved in the pathogenesis of inflammatory pain still need further study in the future. Because a nonspecific promotor CBG was used in pAAV2‐CBG‐EGFP‐3xFLAG‐WPRE‐H1‐kdm6b shRNA, both the neurons and glial cells in the DRG and dorsal horn should be transfected by the treatment. Therefore, we cannot precisely discriminate the role of KDM6B expressed in the neurons and the glial cells in the pathogenesis of inflammatory pain in the present study. This also is one important issue that needs to be studied in the future.

## 5 CONCLUSION

Our present study reveals that upregulation of KDM6B via regulating the expression of TNF‐α in the DRG and spinal dorsal horn contributes to the pathogenesis of intraplantar CFA‐induced inflammatory pain. Targeting KDM6B might be a promising strategy for treating chronic inflammatory pain.

## AUTHOR CONTRIBUTIONS

Ji‐Tian Xu conceived the project and supervised all experiments. Yiming Qiao, Liren Li, Liying Bai, and Yan Gao produced the animal model, conducted the behavioral experiments, and carried out the Western blotting and ChIP‐PCR assays. Yiming Qiao, Liren Li, Xueli Wang, Li Wang, and Zongyi Liang carried out the AAV‐EGFP KDM6B shRNA transfection and immunofluorescence staining and analyzed the data. Ji‐Tian Xu, Liren Li, and Yiming Qiao wrote the manuscript. All the authors read and discussed the manuscript.

## FUNDING INFORMATION

This study was funded by the National Natural Science Foundation of China (Grant Numbers: 82171237 and 81571079).

## CONFLICT OF INTEREST STATEMENT

The authors declare no potential conflicts of interest with respect to the research, authorship, and publication of this article.

## CONSENT FOR PUBLICATION

The study did not involve human subjects so there is no need for consent to participate.

## Supporting information


Appendix S1.

## Data Availability

The data that support the findings of this study are available from the corresponding author upon reasonable request. The source files that support the article have been placed in the Supplementary Materials.
