# Supplementary material for: Upregulation of lysine‐specific demethylase 6B aggravates inflammatory pain through H3K27me3 demethylation‐dependent production of TNF‐α in the dorsal root ganglia and spinal dorsal horn in rats
Source: CNS Neurosci Ther. 2023 Jun 8;29(11):3479–92. doi: 10.1111/cns.14281 (PMC10580362; doi:10.1111/cns.14281)
Supplement: Supplementary file 1 — Appendix S1. [file CNS-29-3479-s001.pdf]

Full unedited images for Western blot:

Full unedited blot for Figure 1C KDM6B

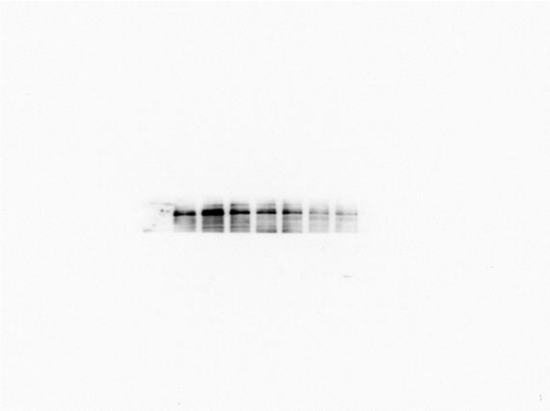

Full unedited blot for Figure 1C ACTIN

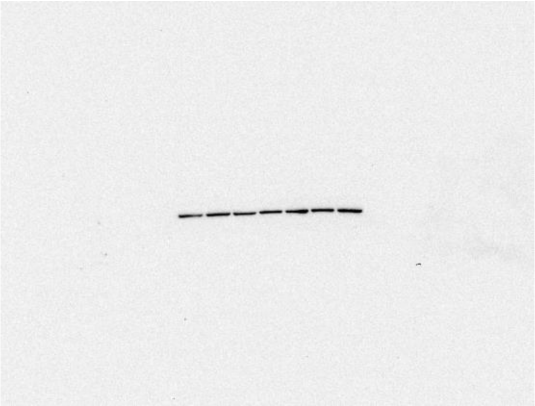

Full unedited blot for Figure 1E H3K27me3

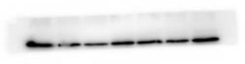

Full unedited blot for Figure 1E H3

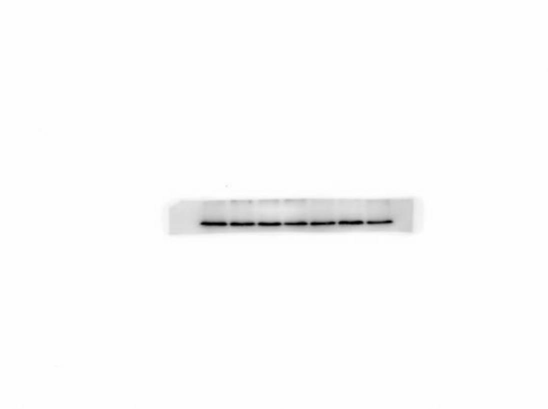

Full unedited blot for Figure 1K KDM6B

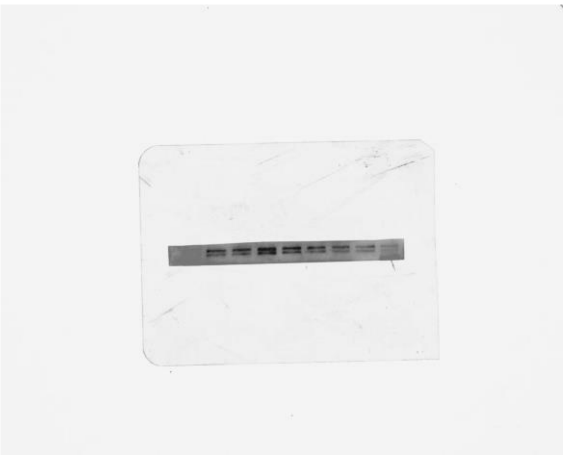

Full unedited blot for Figure 1K ACTIN

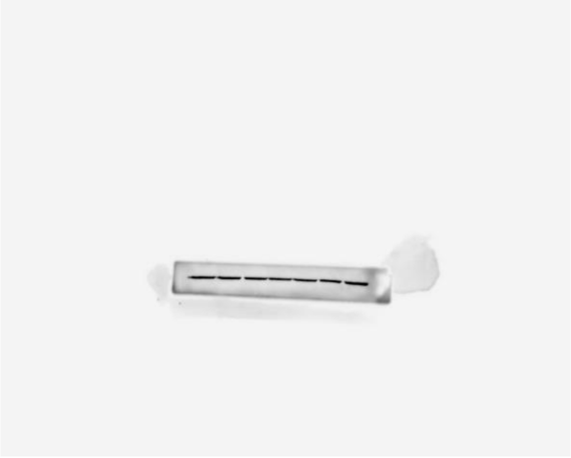

Full unedited blot for Figure 1O H3K27me3

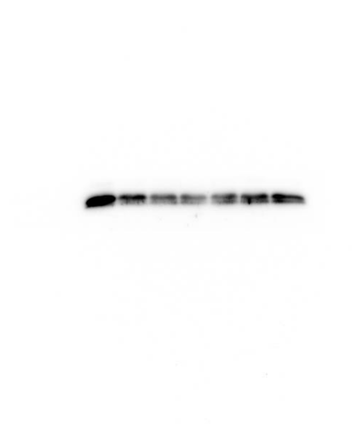

Full unedited blot for Figure 1O H3

Full unedited blot for Figure 3E TNF-α

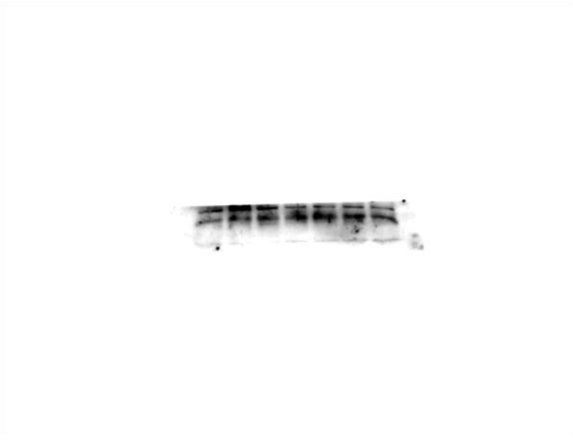

Full unedited blot for Figure 3E ACTIN

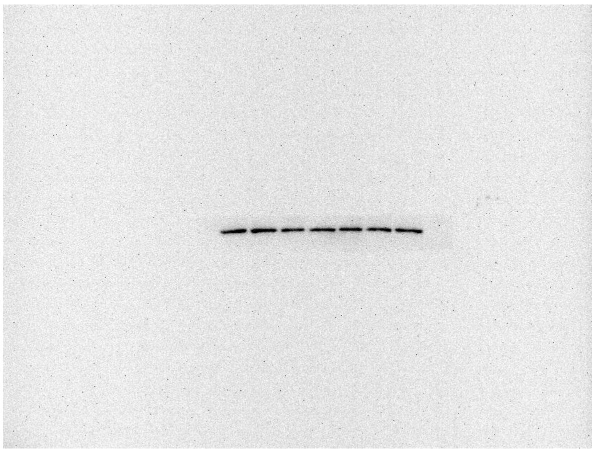

Full unedited blot for Figure 3F TNF-α

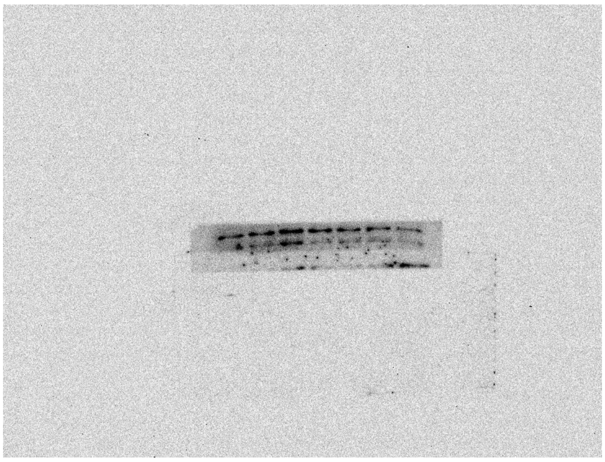

Full unedited blot for Figure 3F ACTIN

Full unedited blot for Figure 3G TNF-α

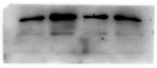

Full unedited blot for Figure 3G ACTIN

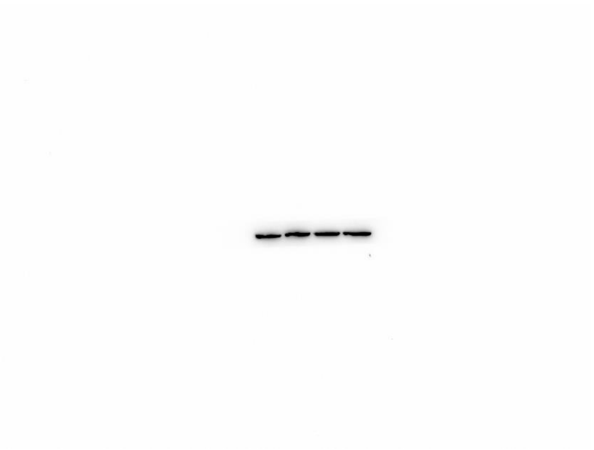

Full unedited blot for Figure 3H TNF-α

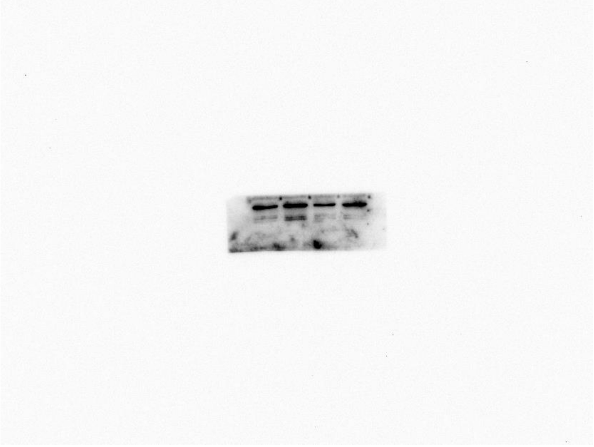

Full unedited blot for Figure 3H ACTIN

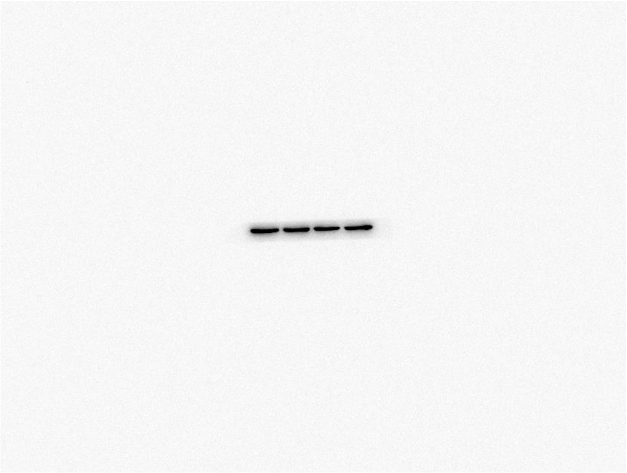

Full unedited blot for Figure 4C KDM6B

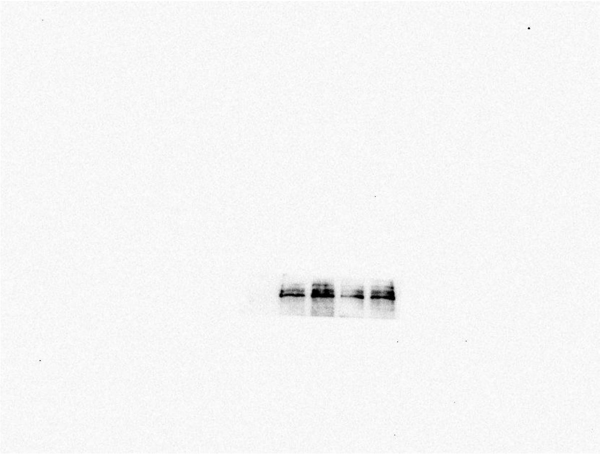

Full unedited blot for Figure 4C TNF-α

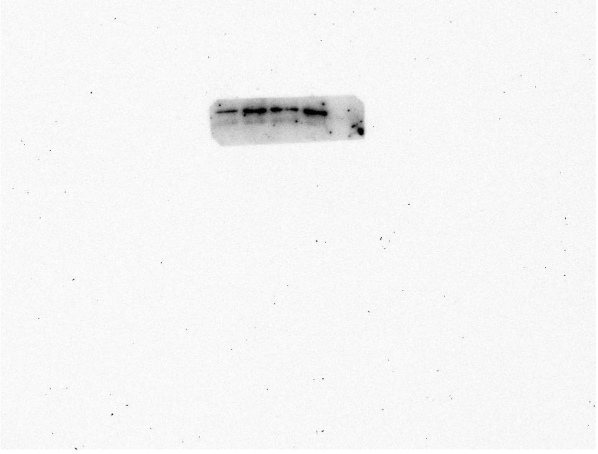

Full unedited blot for Figure 4C ACTIN

Full unedited blot for Figure 4I KDM6B

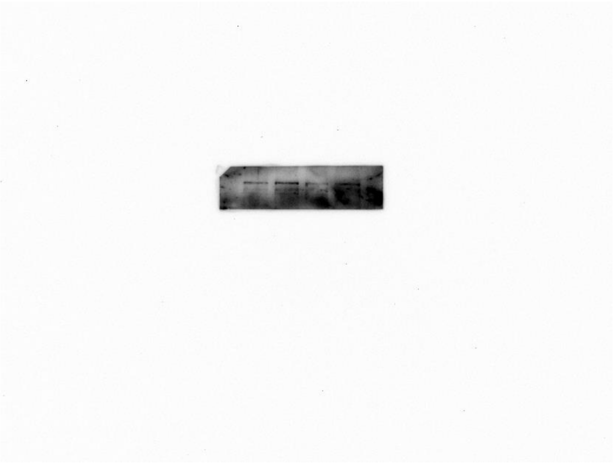

Full unedited blot for Figure 4I TNF-α

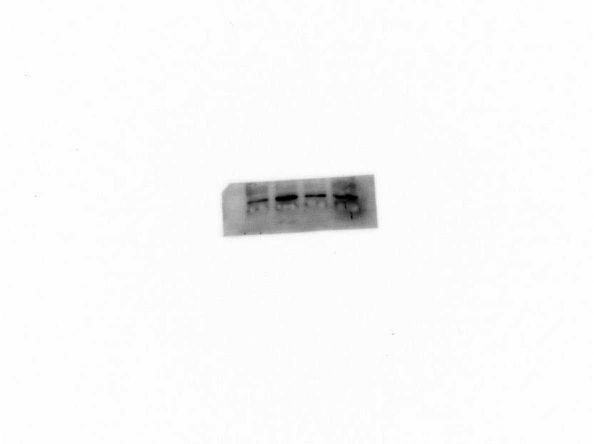

Full unedited blot for Figure 4I ACTIN

Full unedited blot for Figure 6D H3K27me3

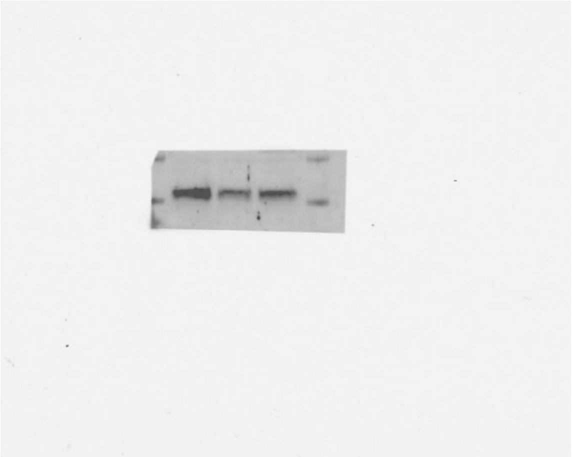

Full unedited blot for Figure 6D H3

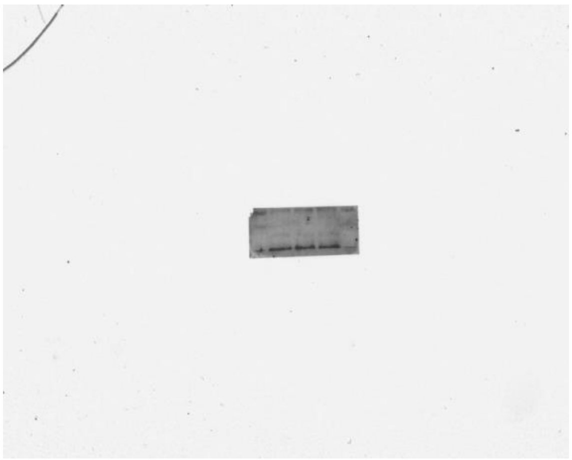

Full unedited blot for Figure 6G H3K27me3

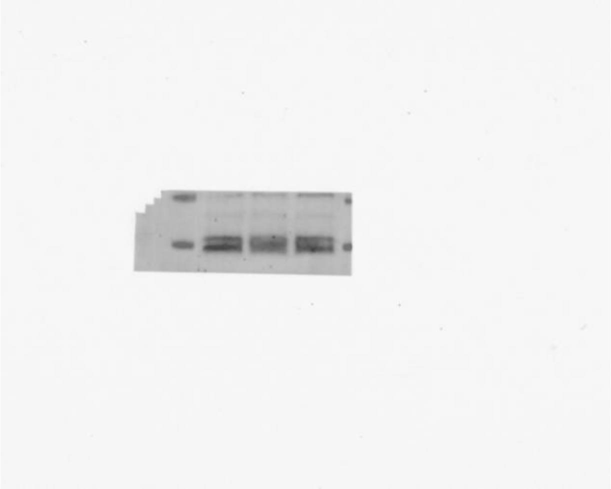

Full unedited blot for Figure 6G H3

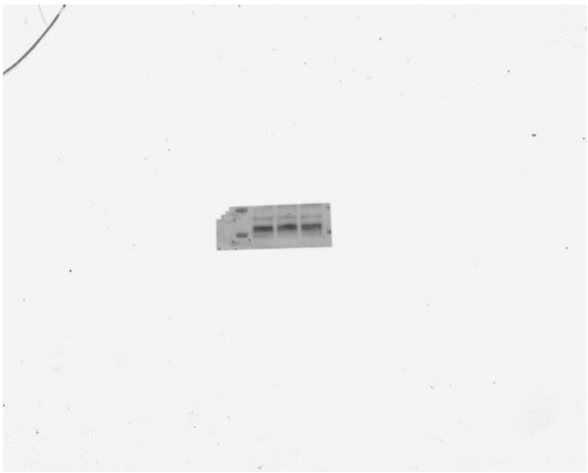

Full unedited blot for Figure 6H H3K27me3

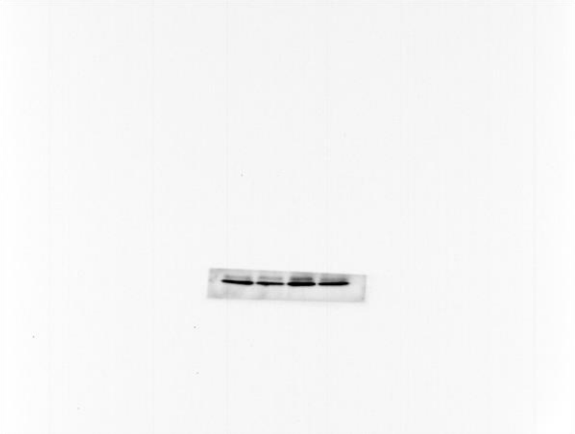

Full unedited blot for Figure 6H H3

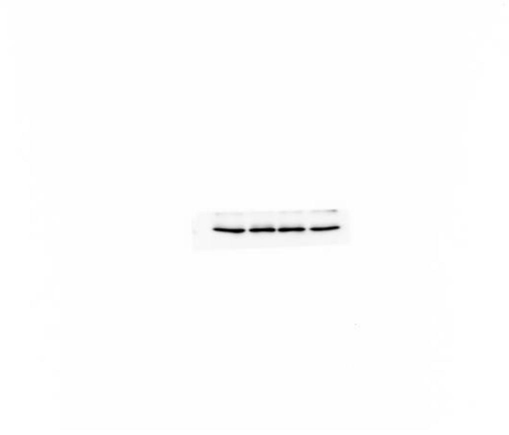

Full unedited blot for Figure 6I H3K27me3

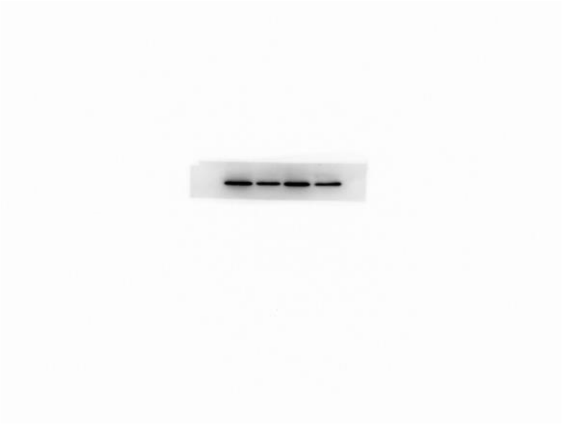

Full unedited blot for Figure 6I H3

Full unedited blot for Figure 6J p-p65

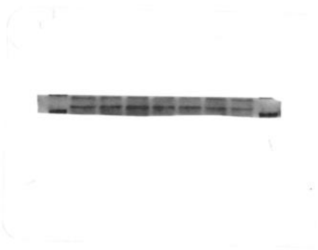

Full unedited blot for Figure 6J t-p65

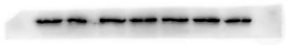

Full unedited blot for Figure 6K p-p65

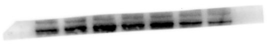

Full unedited blot for Figure 6K t-p65

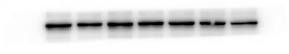

Full unedited images for immunofluorescences staining:

Full unedited image for Figure 1F

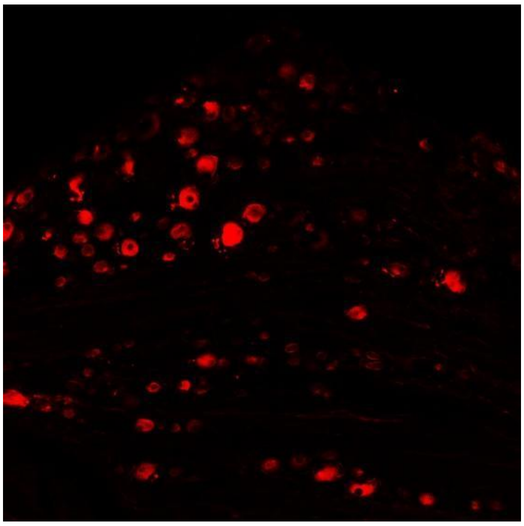

Full unedited image for Figure 1G

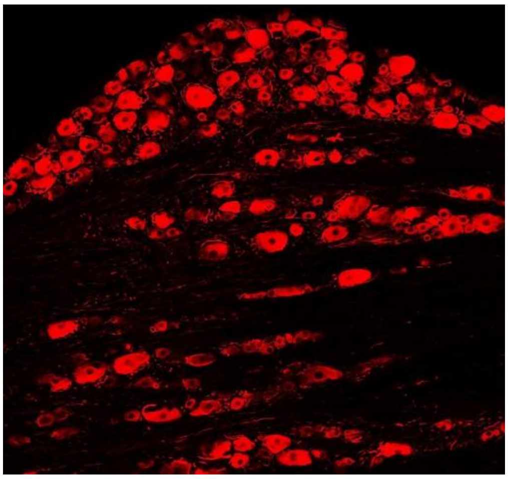

Full unedited image for Figure 1H

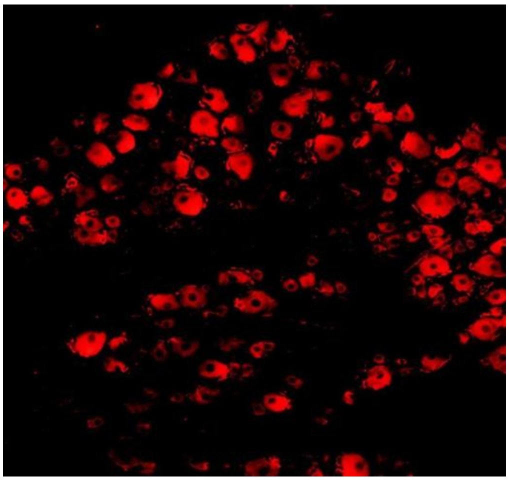

Full unedited image for Figure 1I

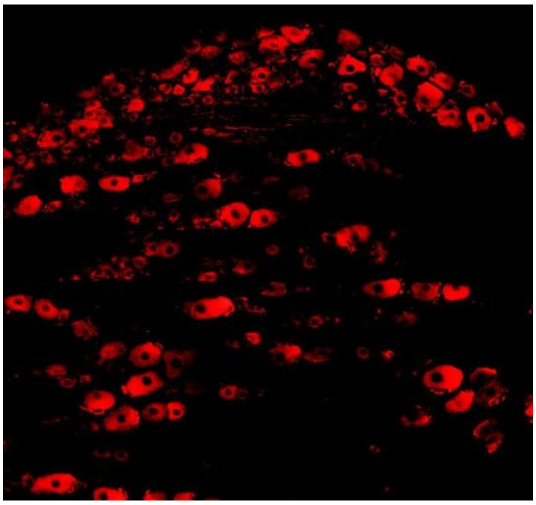

Full unedited image for Figure 1P

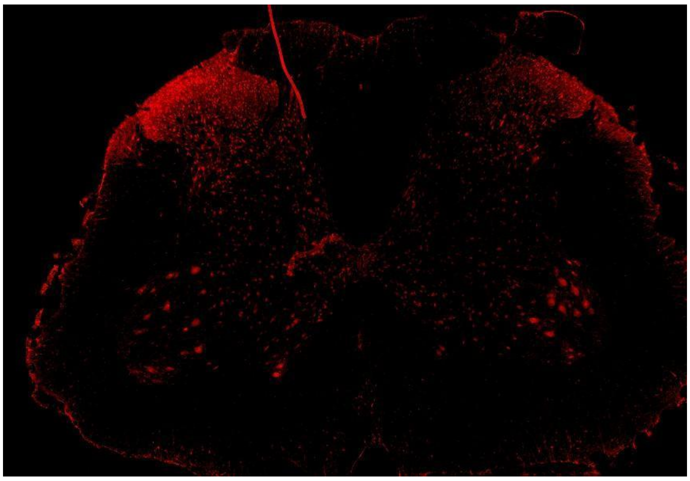

Full unedited image for Figure 1Q

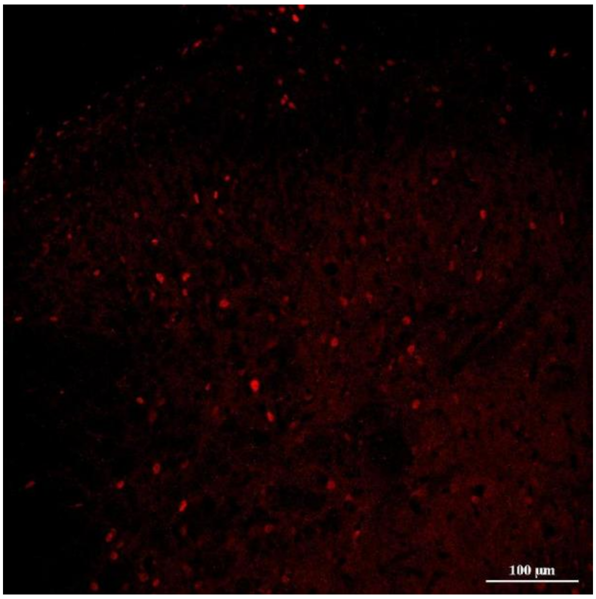

Full unedited image for Figure 1R

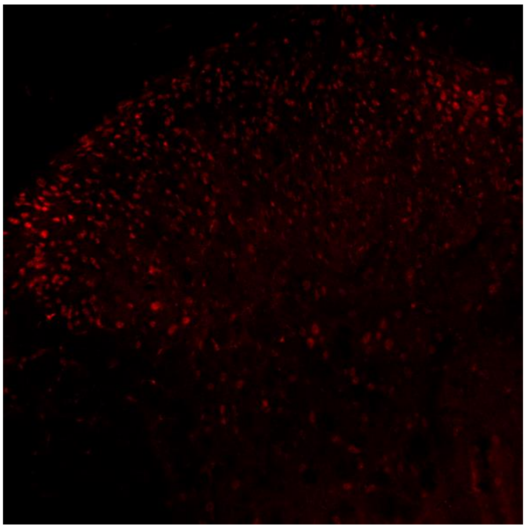

Full unedited image for Figure 1S

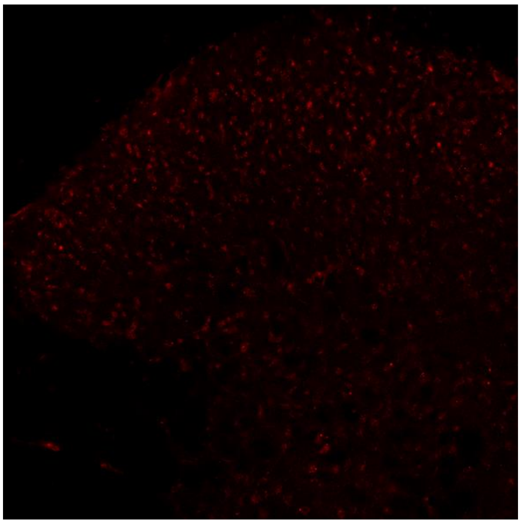

Full unedited image for Figure 1T

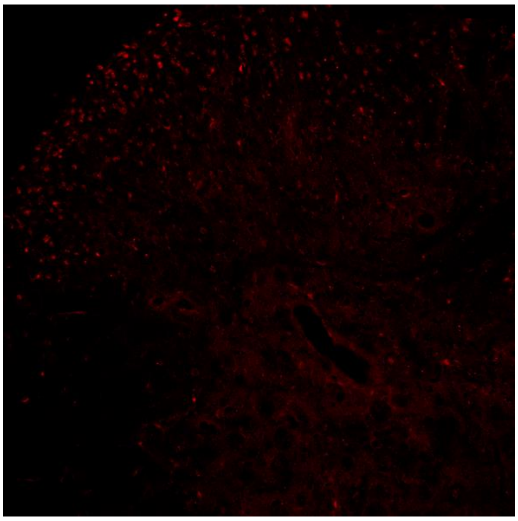

Full unedited image for Figure 2A

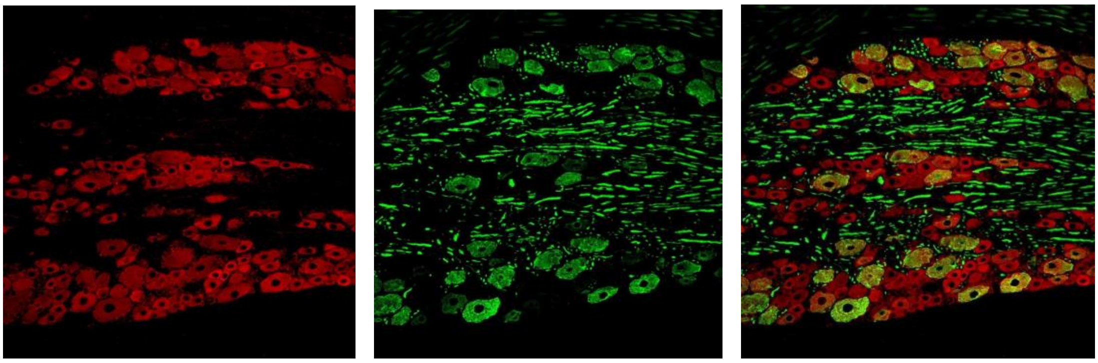

Full unedited image for Figure 2B

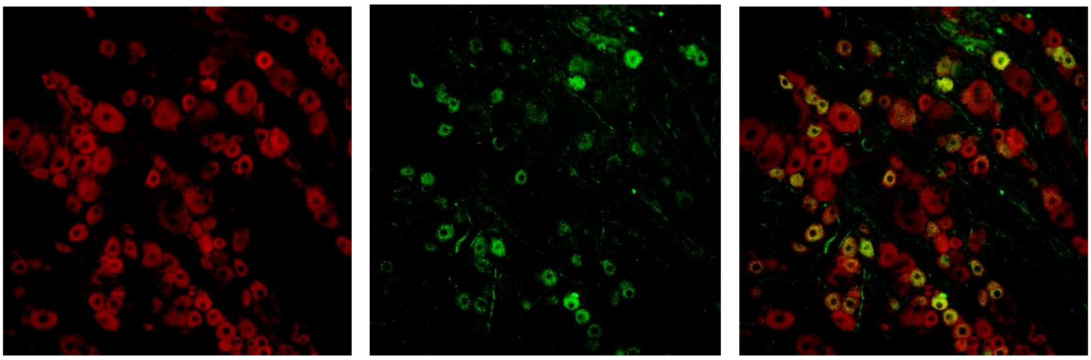

Full unedited image for Figure 2C

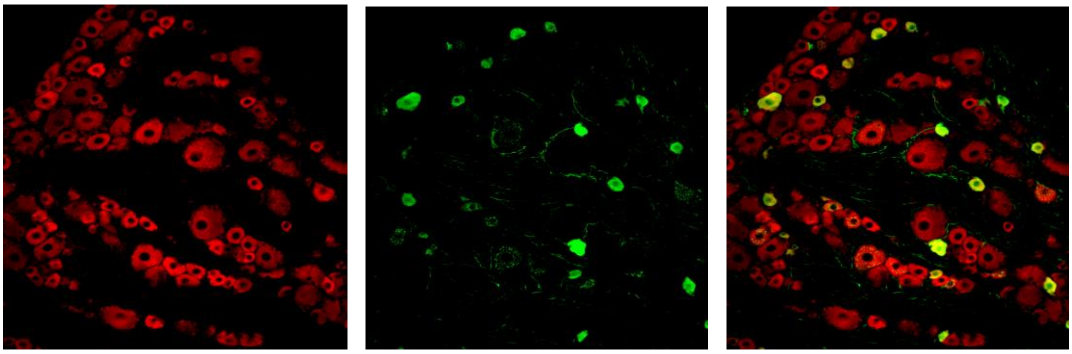

Full unedited image for Figure 2D

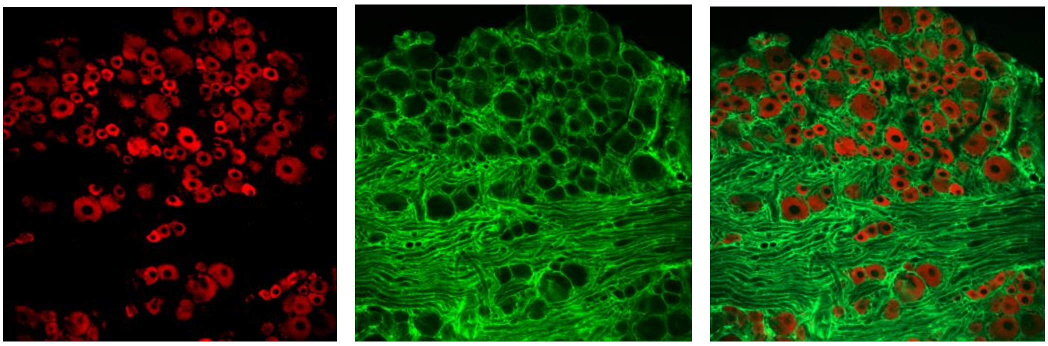

Full unedited image for Figure 2F-I

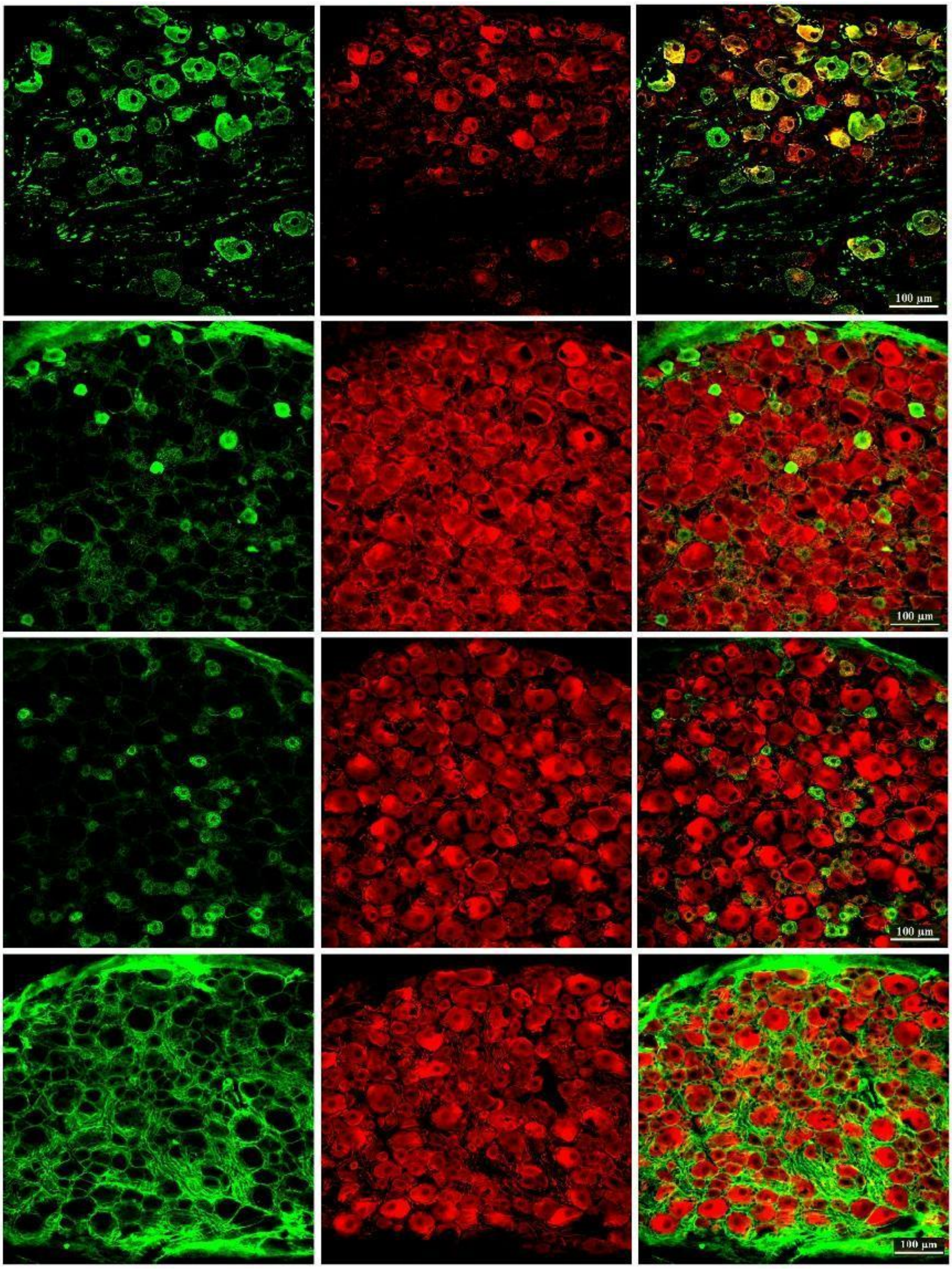

Full unedited image for Figure 2K

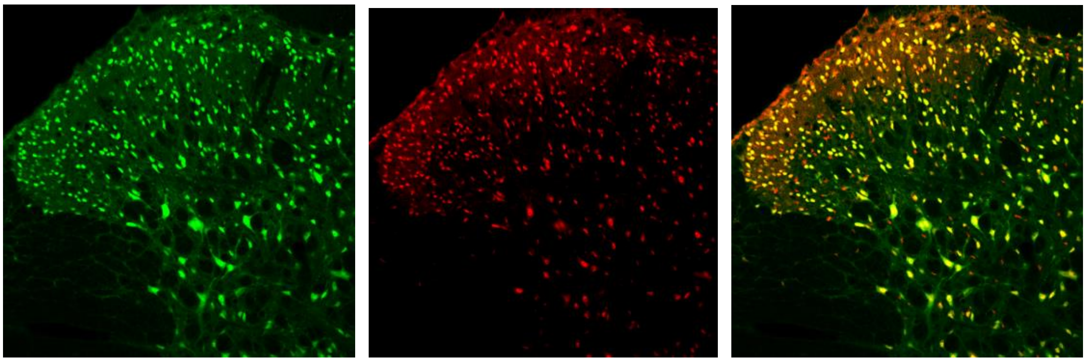

Full unedited image for Figure 2L

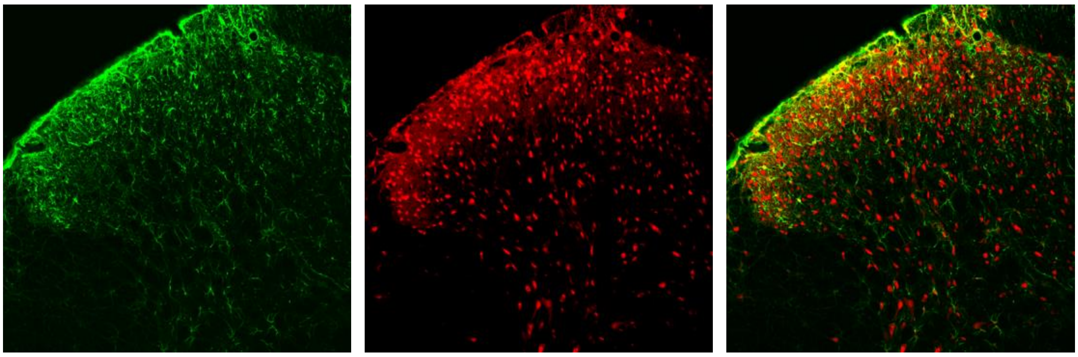

Full unedited image for Figure 2O

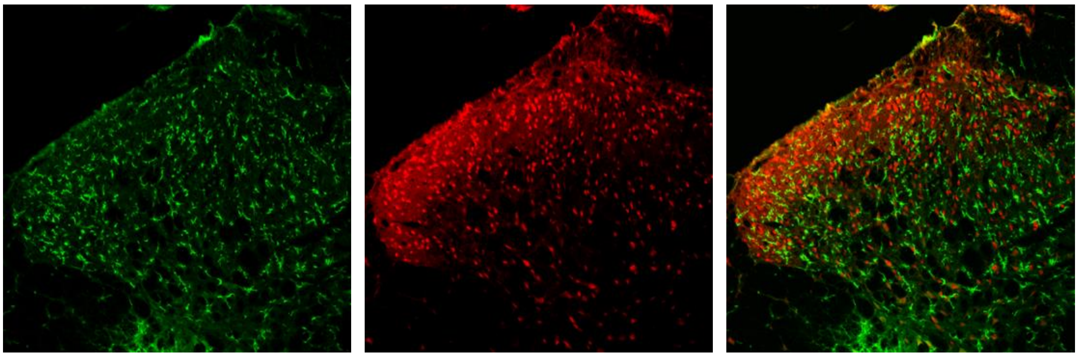

Full unedited image for Figure 2P

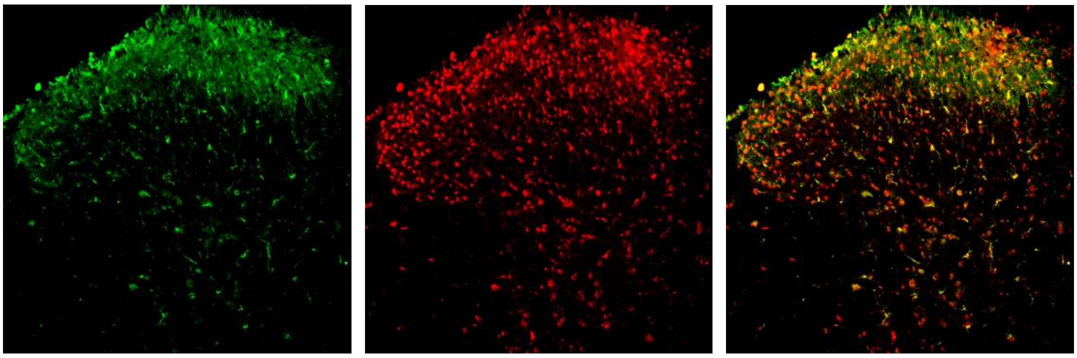

Full unedited image for Figure 2R-U

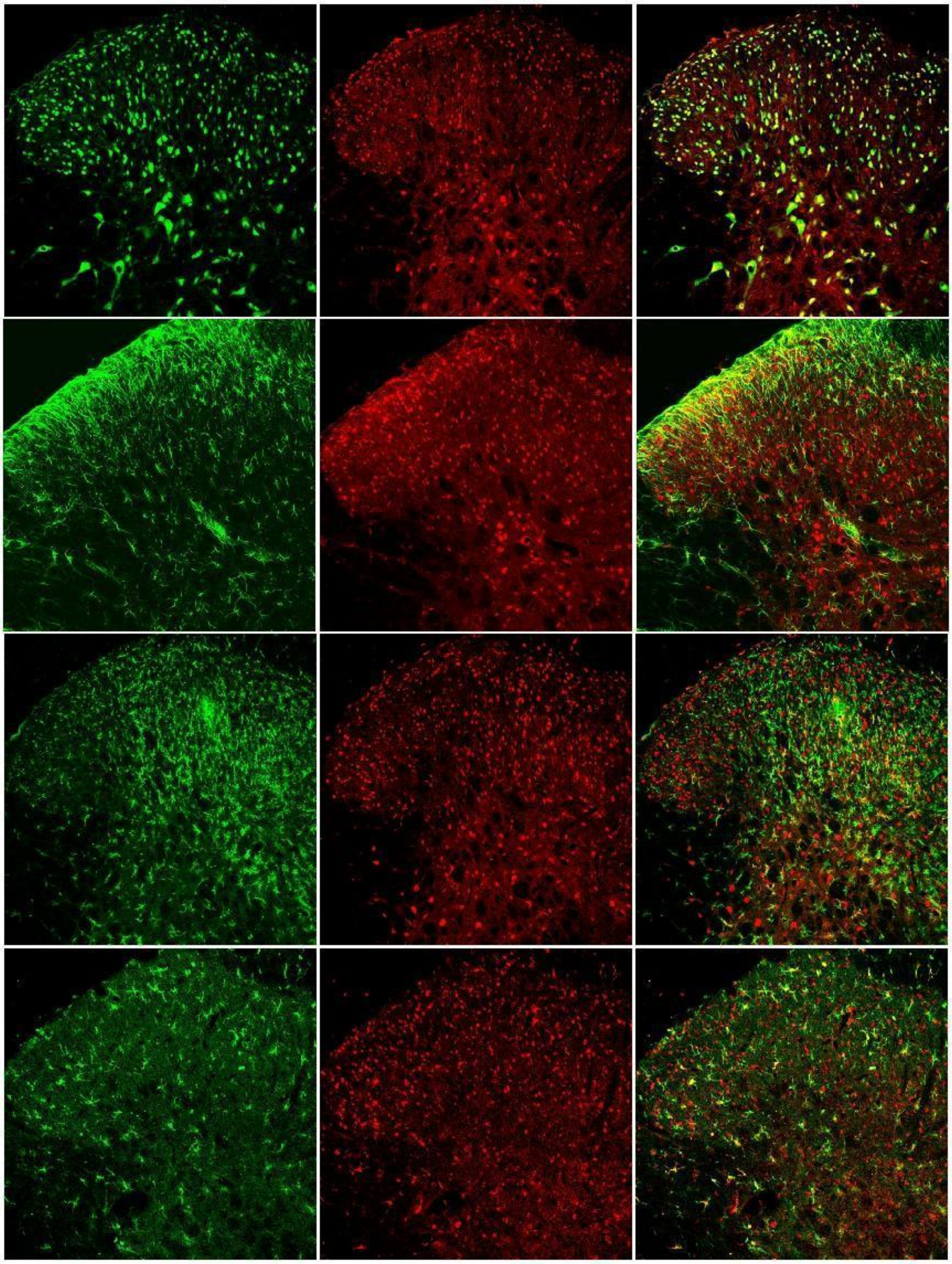

Full unedited image for Figure 4E

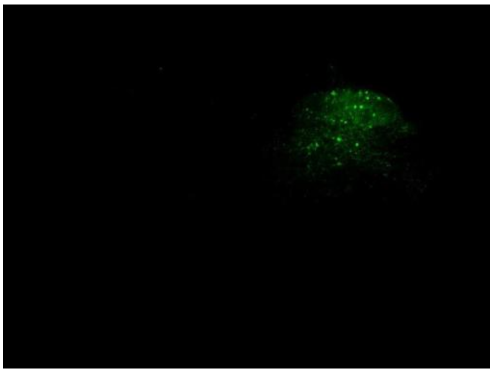

Full unedited image for Figure 4F

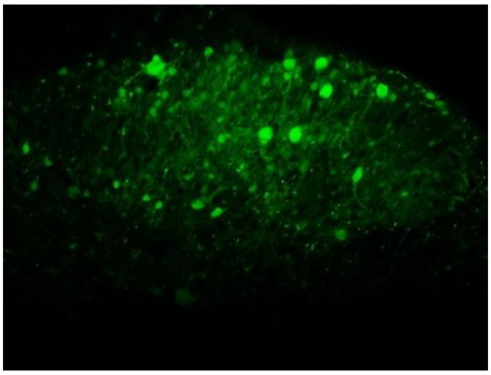

Full unedited image for Figure 4K

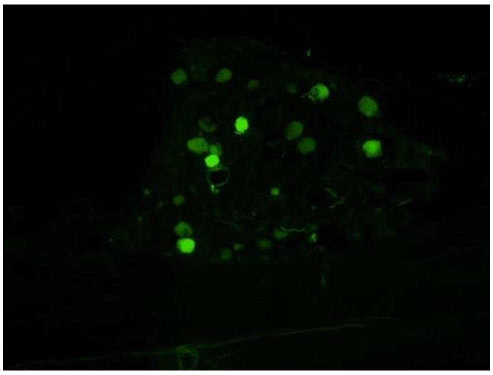

Full unedited image for Figure 4L

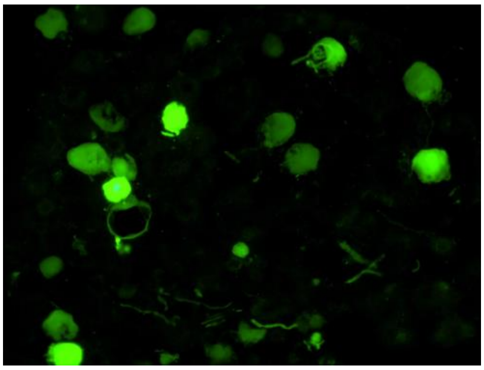

Full unedited image for Figure 5A-D

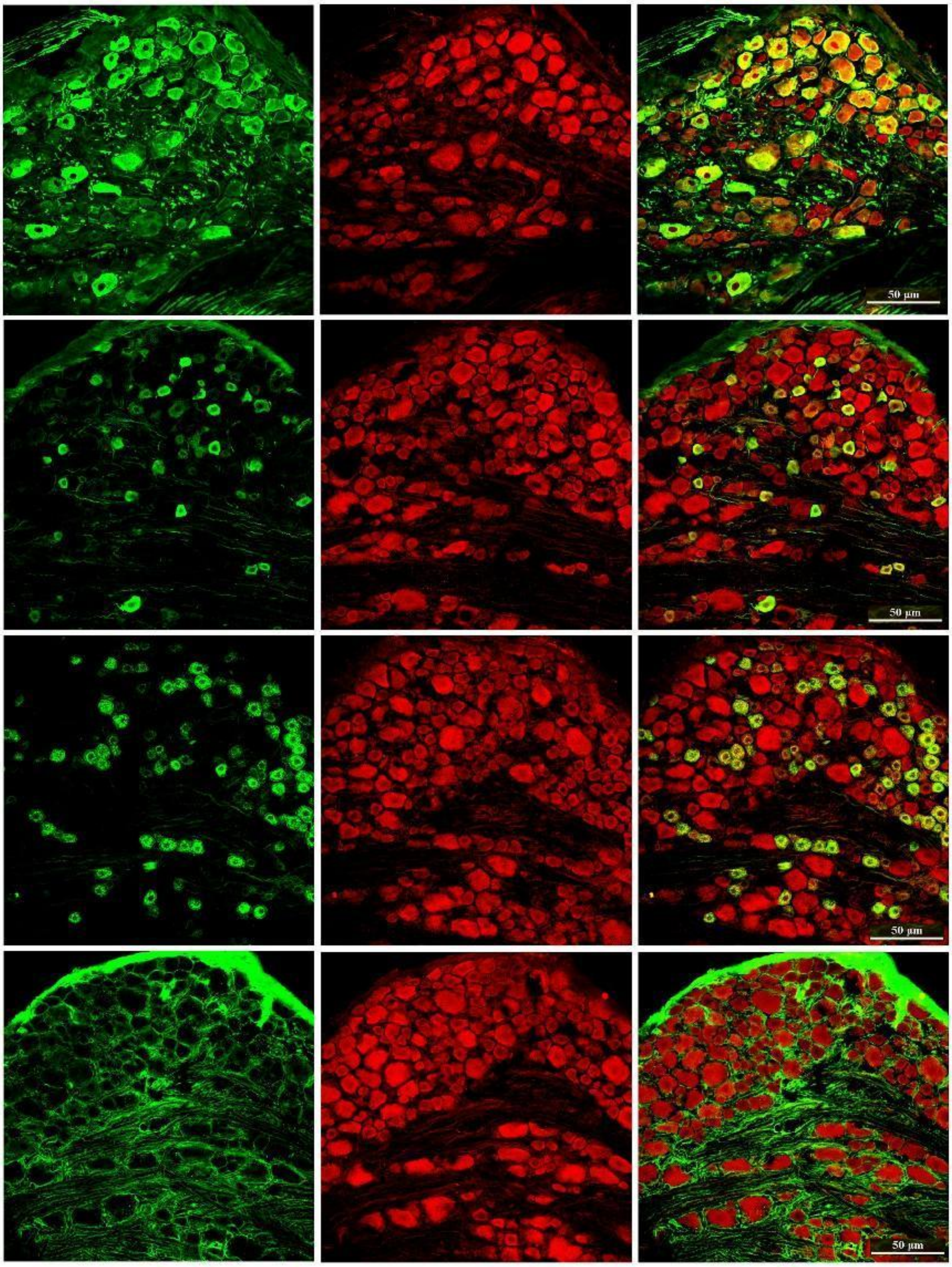

Full unedited image for Figure 5F-I

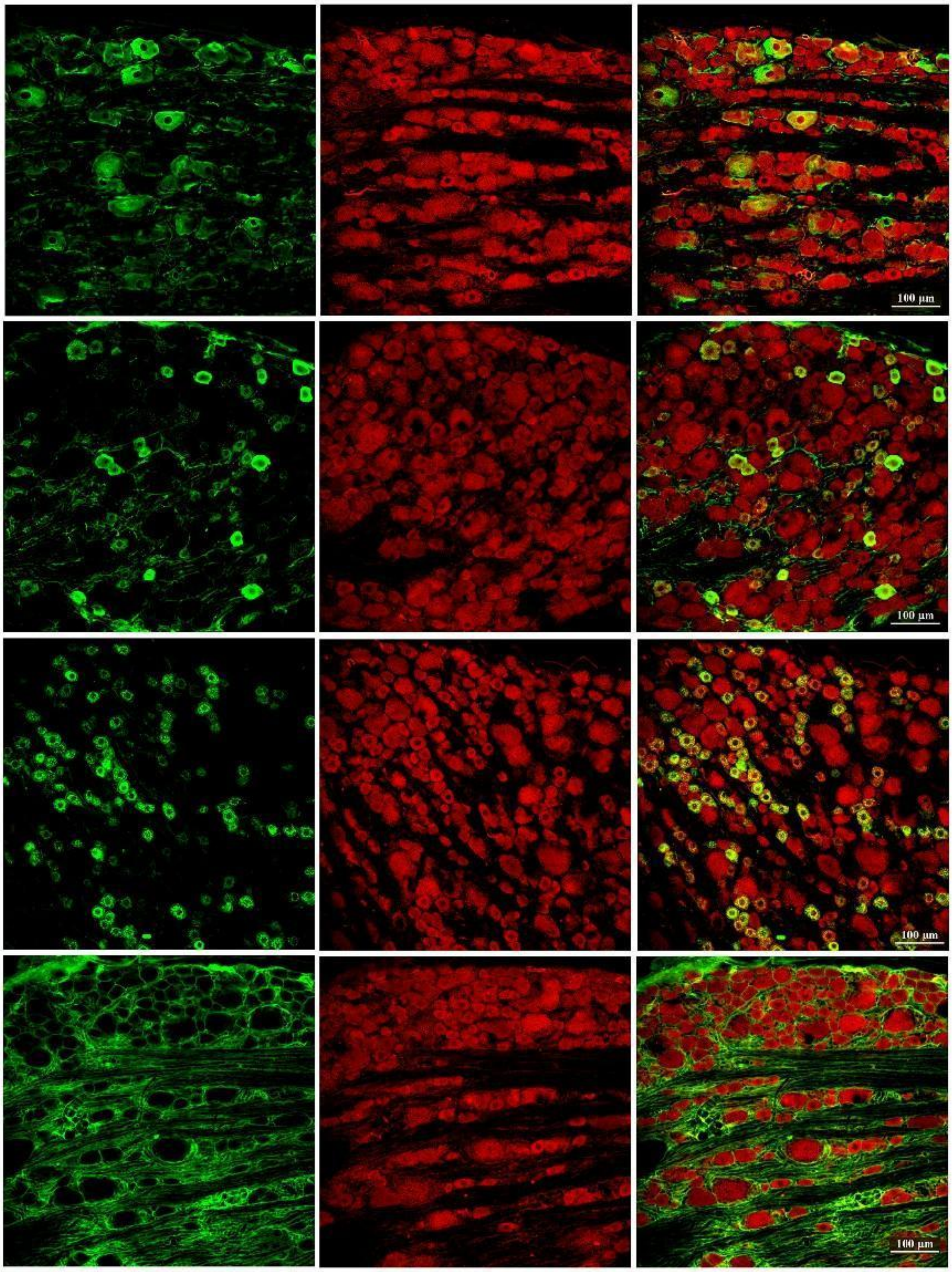

Full unedited image for Figure 5K-P

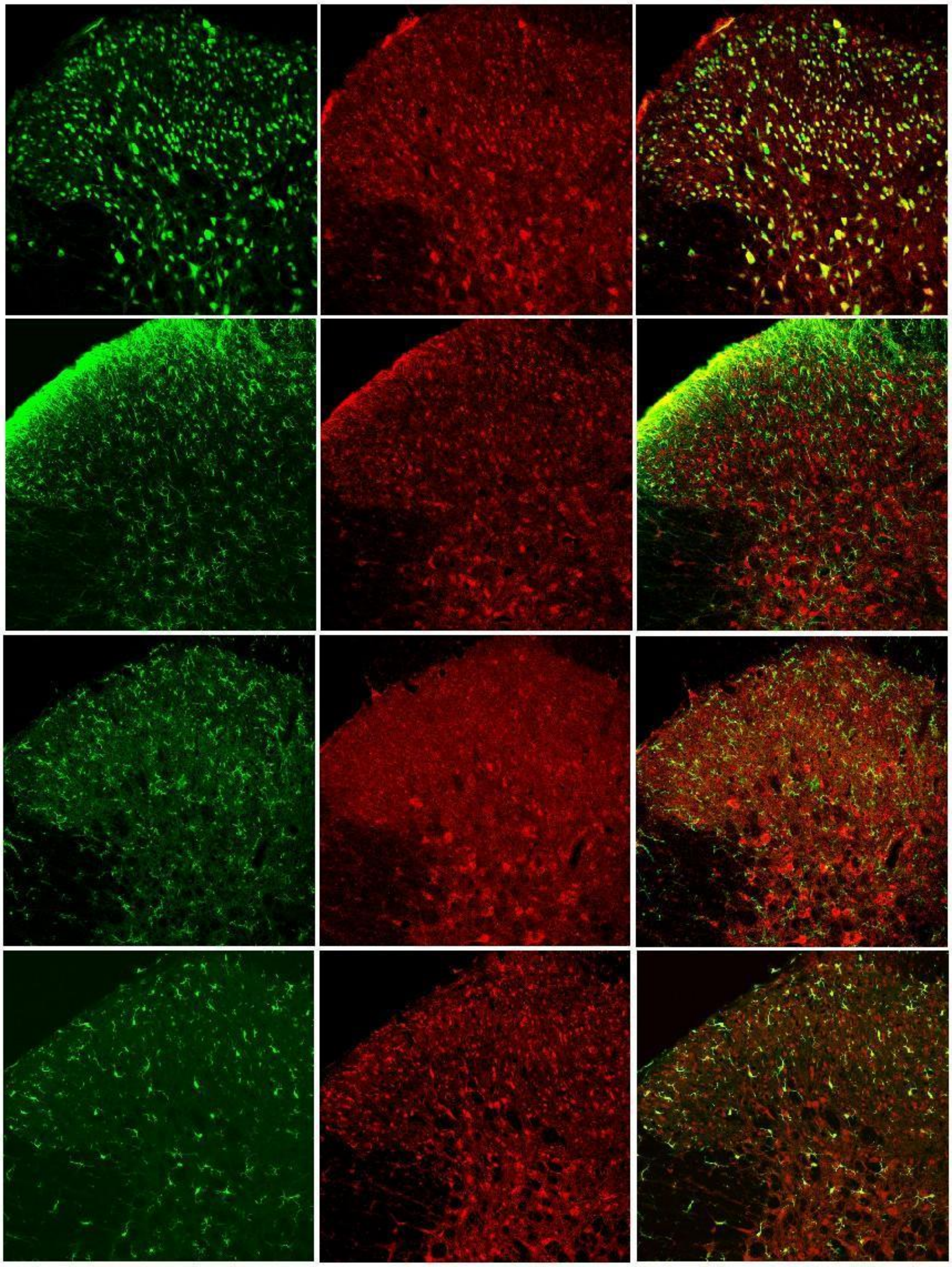

Full unedited image for Figure 5R-U

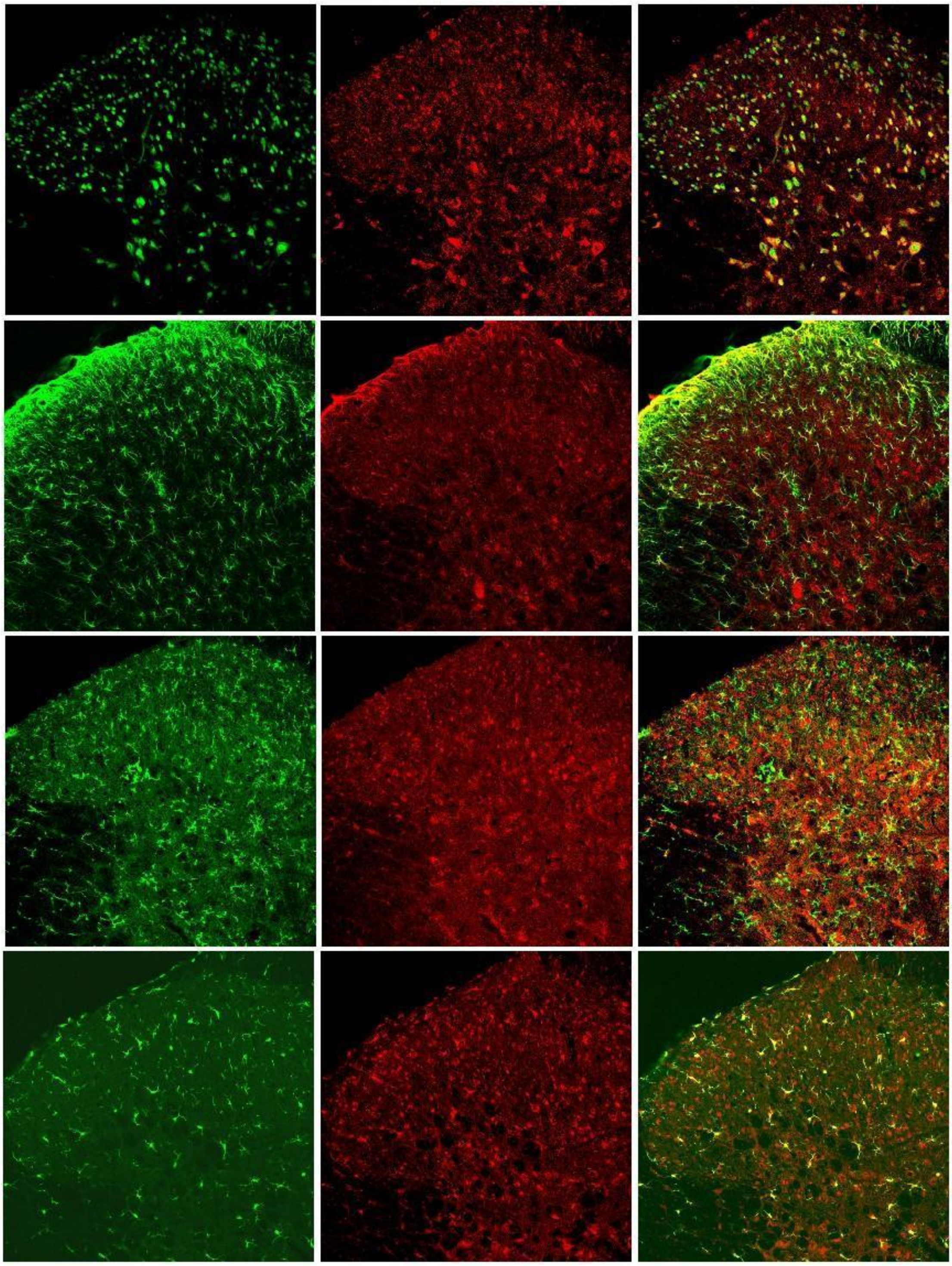

Full unedited images for ChIP-PCR:

Full unedited gel for Figure 6A

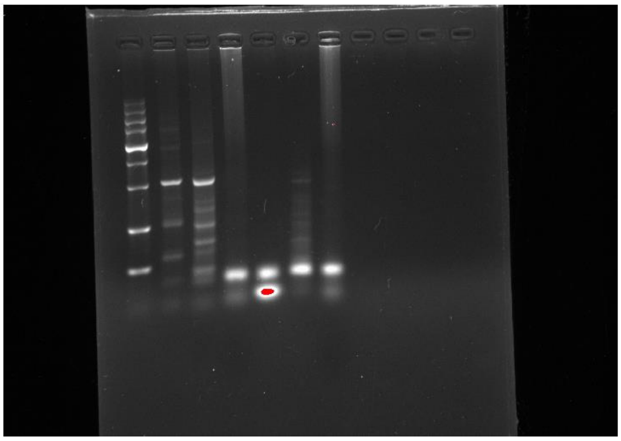

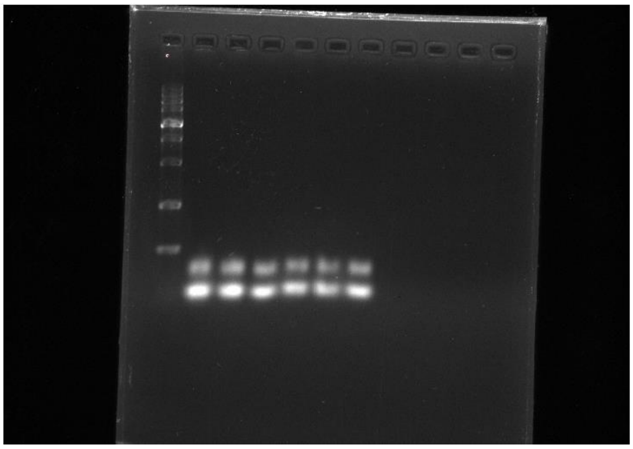

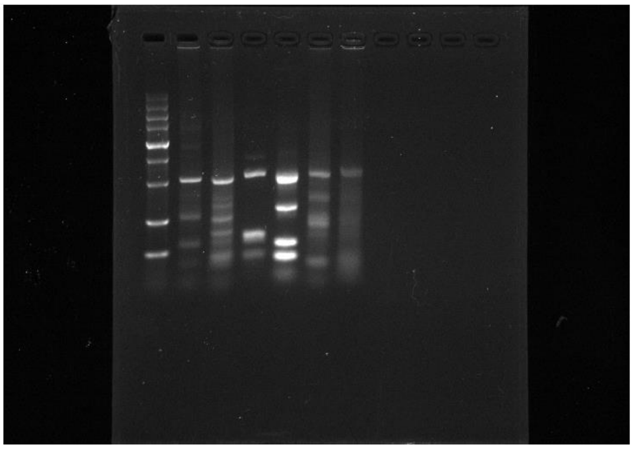

Full unedited gel for Figure 6B

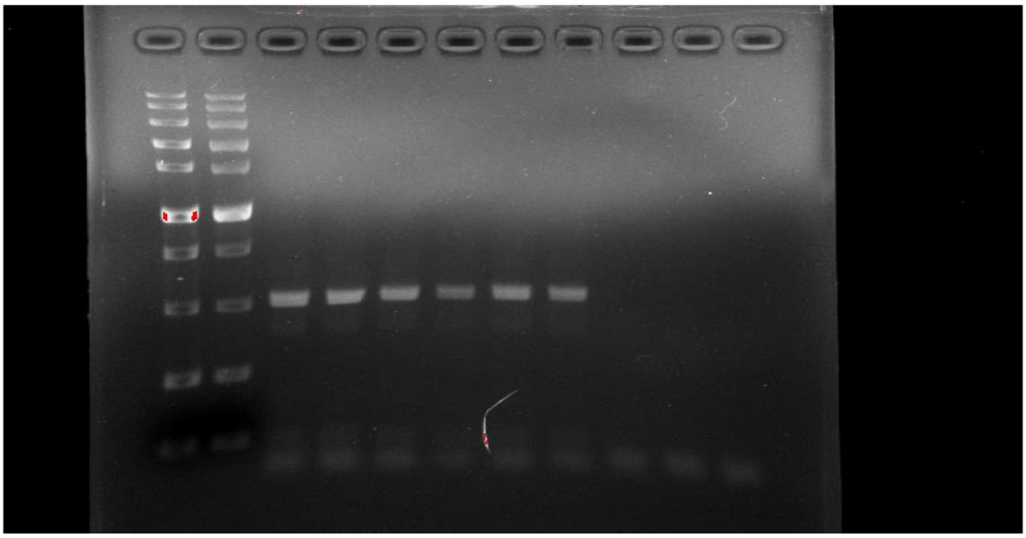

Full unedited gel for Figure 6E

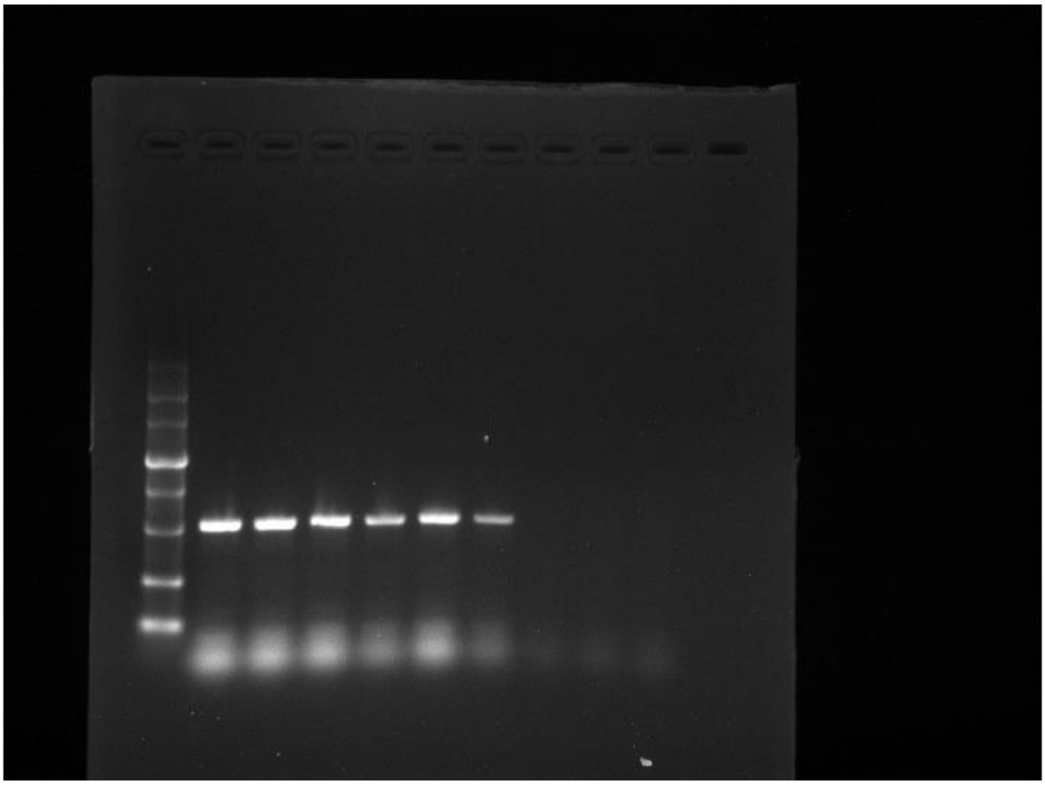

The normalization tests and statistical analysis for all the data in the manuscript:

Figure 1A

Figure 1A PWT	-3	-1	6 h	1	2	3	5	7	10	14	21
CFA (Ipsi)	25.368	25.368	4.338	6.736	6.736	6.736	11.978	6.736	11.978	17.81	17.81
	17.81	17.81	2.805	4.338	4.338	4.338	4.338	6.736	11.978	11.978	17.81
	25.368	25.368	4.338	6.736	6.736	4.338	6.736	4.338	6.736	11.978	17.81
	25.368	25.368	2.805	4.338	4.338	4.338	6.736	6.736	6.736	11.978	15.452
	25.368	25.368	2.805	2.805	4.338	4.338	4.338	6.736	6.736	11.978	15.452
	25.368	25.368	4.338	4.338	6.736	4.338	6.736	6.736	11.978	17.81	17.81
	17.81	17.81	4.338	4.338	6.736	6.736	6.736	6.736	11.978	11.978	17.81
	25.368	25.368	4.338	4.338	6.736	6.736	6.736	4.338	6.736	11.978	17.81
NS (Ipsi)	25.368	25.368	25.368	25.368	17.81	25.368	25.368	25.368	25.368	17.81	25.368
	25.368	25.368	17.81	25.368	25.368	25.368	25.368	25.368	25.368	25.368	17.81
	25.368	17.81	17.81	25.368	17.81	25.368	25.368	25.368	25.368	25.368	25.368
	17.81	25.368	25.368	25.368	25.368	25.368	25.368	17.81	17.881	25.368	25.368
	25.368	25.368	25.368	25.368	25.368	25.368	25.368	25.368	25.368	25.368	25.368
	25.368	17.81	25.368	17.81	25.368	25.368	25.368	25.368	25.368	25.368	25.368
	25.368	25.368	25.368	25.368	25.368	25.368	25.368	25.368	25.368	25.368	25.368
	17.81	25.368	17.81	17.81	25.368	17.81	17.81	17.81	17.81	17.81	25.368
CFA (Contro)	22.61	21.74	21.19	23.01	22.37	22.27	25.5	23.48	22.11	25.79	22.59
	23.59	23.78	24.06	24.96	22.99	23.11	25.62	21.86	22.29	25.45	24.89

	23.45	21.86	22.68	24.33	23.73	22.22	21.61	21.01	22.65	23.66	25.92
	22.93	21.21	22.13	24.73	25.45	22.06	24.01	23.3	21.31	22.79	23.74
	24.67	23.2	23.59	23.32	24.42	25.4	24.32	21.19	25.19	23.67	25.6
	22.27	24.16	24.49	25.06	22.5	21.7	22.64	21.46	22.92	23.8	24.13
	25.62	22.81	24.96	23.44	23.56	25.52	22.25	25.12	22.61	24.5	22.66
	22.56	21.01	25.53	24.37	23.18	22.08	21.48	21.64	25.54	24.98	21.53
NS (Contro)	22.39	22.07	21.17	21.74	24.72	21.58	22.35	23.38	21.83	21.44	25.16
	21.82	23.36	22.03	23.41	22.25	24.21	22.11	22.61	21.72	25.87	21.39
	24.83	22.8	24.18	21.83	21.04	21.37	25.87	24.8	25.6	21.59	22.44
	23.88	24.17	22.94	22.26	22.14	21.15	22.04	21.17	24.23	22.66	25.36
	23.48	21.04	21.03	25.33	25.82	23.6	24.96	22.11	25.91	23.71	21.74
	21.14	23.71	22.07	22.39	25.72	23.64	22.27	23.36	24.42	22.05	21.84
	23.58	25.34	22.1	22.49	25.83	25.89	24.37	25.41	22.98	25.23	25.77
	21.2	22.85	25.58	24.51	25.86	24.63	21.88	22.03	25.16	24.82	24.79

**PWT -3 d**				
	CFA (Ipsi)	NS (Ipsi)	CFA (Control)	NS (Contro)
Test for normal distribution				
Shapiro-Wilk test				
W	0.5659	0.5659	0.8941	0.9298
P value	<0.0001	<0.0001	0.2552	0.5147
Passed normality test (alpha=0.05)?	No	No	Yes	Yes
P value summary	****	****	ns	ns
Number of values	8	8	8	8
**Data does not exhibit a normal distribution, so use a non-parametric equivalent-the Kruskal-Wallis test.**				

Kruskal-Wallis test				
P value	0.1993			
Exact or approximate P value?	Approximate			
P value summary	ns			
Do the medians vary signif. (P < 0.05)?	No			
Number of groups	4			
Kruskal-Wallis statistic	4.65			
Dunn's multiple comparisons test	Mean rank diff.	Significant?	Summary	Adjusted P Value
NS (Ipsi) vs. CFA (Ipsi)	0	No	ns	>0.9999
NS (Ipsi) vs. CFA (Control)	4.75	No	ns	0.8931
NS (Ipsi) vs. NS (Contro)	8.25	No	ns	0.2115
**PWT -1 d**				
	CFA (Ipsi)	NS (Ipsi)	CFA (Control)	NS (Contro)
Test for normal distribution				
Shapiro-Wilk test				
W	0.5659	0.5659	0.9314	0.9924
P value	<0.0001	<0.0001	0.5291	0.9979
Passed normality test (alpha=0.05)?	No	No	Yes	Yes
P value summary	****	****	ns	ns
Number of values	8	8	8	8
**Data does not exhibit a normal distribution, so use a non-parametric equivalent-the Kruskal-Wallis test.**				
Kruskal-Wallis test				
P value	0.0916			
Exact or approximate P value?	Approximate			
P value summary	ns			
Do the medians vary signif. (P < 0.05)?	No			
Number of groups	4			
Kruskal-Wallis statistic	6.452			
Dunn's multiple comparisons test	Mean rank diff.	Significant?	Summary	Adjusted P Value
NS (Ipsi) vs. CFA (Ipsi)	0	No	ns	>0.9999
NS (Ipsi) vs. CFA (Control)	9.25	No	ns	0.1277
NS (Ipsi) vs. NS (Contro)	6.75	No	ns	0.4168

**PWT 6 h**				
	CFA (Ipsi)	NS (Ipsi)	CFA (Control)	NS (Contro)
Test for normal distribution				
Shapiro-Wilk test				
W	0.6412	0.6412	0.9704	0.8872
P value	0.0005	0.0005	0.9014	0.2205
Passed normality test (alpha=0.05)?	No	No	Yes	Yes
P value summary	***	***	ns	ns
Number of values	8	8	8	8
**Data does not exhibit a normal distribution, so use a non-parametric equivalent-the Kruskal-Wallis test.**				
Kruskal-Wallis test				
P value	0.0004			
Exact or approximate P value?	Approximate			
P value summary	***			
Do the medians vary signif. (P < 0.05)?	Yes			
Number of groups	4			
Kruskal-Wallis statistic	18.17			
Dunn's multiple comparisons test	Mean rank diff.	Significant?	Summary	Adjusted P Value
NS (Ipsi) vs. CFA (Ipsi)	16.75	Yes	**	0.001
NS (Ipsi) vs. CFA (Control)	-0.5	No	ns	>0.9999
NS (Ipsi) vs. NS (Contro)	2.75	No	ns	>0.9999
**PWT 1 d**				
	CFA (Ipsi)	NS (Ipsi)	CFA (Control)	NS (Contro)
Test for normal distribution				
Shapiro-Wilk test				
W	0.786	0.5659	0.8988	0.8668
P value	0.0202	<0.0001	0.2818	0.1402
Passed normality test (alpha=0.05)?	No	No	Yes	Yes
P value summary	*	****	ns	ns
Number of values	8	8	8	8
**Data does not exhibit a normal distribution, so use a non-parametric equivalent-the Kruskal-Wallis test.**				
Kruskal-Wallis test				
P value	0.0001			

Exact or approximate P value?	Approximate			
P value summary	***			
Do the medians vary signif. (P < 0.05)?	Yes			
Number of groups	4			
Kruskal-Wallis statistic	20.67			
Dunn's multiple comparisons test	Mean rank diff.	Significant?	Summary	Adjusted P Value
NS (Ipsi) vs. CFA (Ipsi)	20	Yes	****	<0.0001
NS (Ipsi) vs. CFA (Control)	3.875	No	ns	>0.9999
NS (Ipsi) vs. NS (Contro)	8.125	No	ns	0.2449
**PWT 2 d**				
	CFA (Ipsi)	NS (Ipsi)	CFA (Control)	NS (Contro)
Test for normal distribution				
Shapiro-Wilk test				
W	0.6412	0.5659	0.9366	0.7927
P value	0.0005	<0.0001	0.5781	0.0239
Passed normality test (alpha=0.05)?	No	No	Yes	No
P value summary	***	****	ns	*
Number of values	8	8	8	8
**Data does not exhibit a normal distribution, so use a non-parametric equivalent-the Kruskal-Wallis test.**				
Kruskal-Wallis test				
P value	0.0004			
Exact or approximate P value?	Approximate			
P value summary	***			
Do the medians vary signif. (P < 0.05)?	Yes			
Number of groups	4			
Kruskal-Wallis statistic	18.39			
Dunn's multiple comparisons test	Mean rank diff.	Significant?	Summary	Adjusted P Value
NS (Ipsi) vs. CFA (Ipsi)	16.25	Yes	**	0.0015
NS (Ipsi) vs. CFA (Control)	2.375	No	ns	>0.9999
NS (Ipsi) vs. NS (Contro)	-1.625	No	ns	>0.9999
**PWT 3 d**				
	CFA (Ipsi)	NS (Ipsi)	CFA	NS (Contro)

			(Control)	
Test for normal distribution				
Shapiro-Wilk test				
W	0.6412	0.4184	0.7565	0.9146
P value	0.0005	<0.0001	0.0096	0.3876
Passed normality test (alpha=0.05)?	No	No	No	Yes
P value summary	***	****	**	ns
Number of values	8	8	8	8
**Data does not exhibit a normal distribution, so use a non-parametric equivalent-the Kruskal-Wallis test.**				
Kruskal-Wallis test				
P value	0.0002			
Exact or approximate P value?	Approximate			
P value summary	***			
Do the medians vary signif. (P < 0.05)?	Yes			
Number of groups	4			
Kruskal-Wallis statistic	19.33			
Dunn's multiple comparisons test	Mean rank diff.	Significant?	Summary	Adjusted P Value
NS (Ipsi) vs. CFA (Ipsi)	19.38	Yes	****	<0.0001
NS (Ipsi) vs. CFA (Control)	4.625	No	ns	0.9616
NS (Ipsi) vs. NS (Contro)	5.5	No	ns	0.7125
**PWT 5 d**				
	CFA (Ipsi)	NS (Ipsi)	CFA (Control)	NS (Contro)
Test for normal distribution				
Shapiro-Wilk test				
W	0.7434	0.4184	0.9013	0.8007
P value	0.0069	<0.0001	0.297	0.0291
Passed normality test (alpha=0.05)?	No	No	Yes	No
P value summary	**	****	ns	*
Number of values	8	8	8	8
**Data does not exhibit a normal distribution, so use a non-parametric equivalent-the Kruskal-Wallis test.**				
Kruskal-Wallis test				
P value	0.0002			
Exact or approximate P value?	Approximate			
P value summary	***			

Do the medians vary signif. (P < 0.05)?	Yes			
Number of groups	4			
Kruskal-Wallis statistic	19.32			
Dunn's multiple comparisons test	Mean rank diff.	Significant?	Summary	Adjusted P Value
NS (Ipsi) vs. CFA (Ipsi)	19.38	Yes	****	<0.0001
NS (Ipsi) vs. CFA (Control)	4.625	No	ns	0.962
NS (Ipsi) vs. NS (Contro)	5.5	No	ns	0.7128
**PWT 7 d**				
	CFA (Ipsi)	NS (Ipsi)	CFA (Control)	NS (Contro)
Test for normal distribution				
Shapiro-Wilk test				
W	0.5659	0.5659	0.8658	0.9499
P value	<0.0001	<0.0001	0.137	0.7104
Passed normality test (alpha=0.05)?	No	No	Yes	Yes
P value summary	****	****	ns	ns
Number of values	8	8	8	8
**Data does not exhibit a normal distribution, so use a non-parametric equivalent-the Kruskal-Wallis test.**				
Kruskal-Wallis test				
P value	0.0002			
Exact or approximate P value?	Approximate			
P value summary	***			
Do the medians vary signif. (P < 0.05)?	Yes			
Number of groups	4			
Kruskal-Wallis statistic	19.71			
Dunn's multiple comparisons test	Mean rank diff.	Significant?	Summary	Adjusted P Value
NS (Ipsi) vs. CFA (Ipsi)	19.25	Yes	***	0.0001
NS (Ipsi) vs. CFA (Control)	6.625	No	ns	0.4652
NS (Ipsi) vs. NS (Contro)	3.125	No	ns	>0.9999
**PWT 10 d**				
	CFA (Ipsi)	NS (Ipsi)	CFA (Control)	NS (Contro)
Test for normal distribution				

Shapiro-Wilk test				
W	0.6647	0.5676	0.8504	0.9064
P value	0.0009	<0.0001	0.0962	0.3296
Passed normality test (alpha=0.05)?	No	No	Yes	Yes
P value summary	***	****	ns	ns
Number of values	8	8	8	8
**Data does not exhibit a normal distribution, so use a non-parametric equivalent-the Kruskal-Wallis test.**				
Kruskal-Wallis test				
P value	0.0003			
Exact or approximate P value?	Approximate			
P value summary	***			
Do the medians vary signif. (P < 0.05)?	Yes			
Number of groups	4			
Kruskal-Wallis statistic	18.54			
Dunn's multiple comparisons test	Mean rank diff.	Significant?	Summary	Adjusted P Value
NS (Ipsi) vs. CFA (Ipsi)	17.75	Yes	***	0.0004
NS (Ipsi) vs. CFA (Control)	4.25	No	ns	>0.9999
NS (Ipsi) vs. NS (Contro)	1	No	ns	>0.9999
**PWT 14 d**				
	CFA (Ipsi)	NS (Ipsi)	CFA (Control)	NS (Contro)
Test for normal distribution				
Shapiro-Wilk test				
W	0.5659	0.5659	0.9525	0.9099
P value	<0.0001	<0.0001	0.7363	0.3534
Passed normality test (alpha=0.05)?	No	No	Yes	Yes
P value summary	****	****	ns	ns
Number of values	8	8	8	8
**Data does not exhibit a normal distribution, so use a non-parametric equivalent-the Kruskal-Wallis test.**				
Kruskal-Wallis test				
P value	0.0005			
Exact or approximate P value?	Approximate			
P value summary	***			
Do the medians vary signif. (P < 0.05)?	Yes			

Number of groups	4			
Kruskal-Wallis statistic	17.82			
Dunn's multiple comparisons test	Mean rank diff.	Significant?	Summary	Adjusted P Value
NS (Ipsi) vs. CFA (Ipsi)	17.25	Yes	***	0.0006
NS (Ipsi) vs. CFA (Control)	0.75	No	ns	>0.9999
NS (Ipsi) vs. NS (Contro)	4	No	ns	>0.9999
**PWT 21 d**				
	CFA (Ipsi)	NS (Ipsi)	CFA (Control)	NS (Contro)
Test for normal distribution				
Shapiro-Wilk test				
W	0.5659	0.4184	0.9568	0.8323
P value	<0.0001	<0.0001	0.779	0.0627
Passed normality test (alpha=0.05)?	No	No	Yes	Yes
P value summary	****	****	ns	ns
Number of values	8	8	8	8
**Data does not exhibit a normal distribution, so use a non-parametric equivalent-the Kruskal-Wallis test.**				
Kruskal-Wallis test				
P value	0.0004			
Exact or approximate P value?	Approximate			
P value summary	***			
Do the medians vary signif. (P < 0.05)?	Yes			
Number of groups	4			
Kruskal-Wallis statistic	18.3			
Dunn's multiple comparisons test	Mean rank diff.	Significant?	Summary	Adjusted P Value
NS (Ipsi) vs. CFA (Ipsi)	18.63	Yes	***	0.0002
NS (Ipsi) vs. CFA (Control)	3.625	No	ns	>0.9999
NS (Ipsi) vs. NS (Contro)	5.75	No	ns	0.6463
Two-way ANOVA	Ordinary			
Alpha	0.05			
Source of Variation	% of total variation	P value	P value summary	Significant?
Interaction	11.13	<0.0001	****	Yes

Row Factor	29.78	<0.0001	****	Yes
Column Factor	56.56	<0.0001	****	Yes

Figure 1B PWL

Figure 1B	-3	-1	6 h	1	2	3	5	7	10	14	21
CFA (Ipsi)	11.367	12.633	3.067	2.4	3.133	3.4	3.433	4.567	9	11.9	12.5
	12.433	14.3	2.933	2.533	3.067	3.533	5.667	4.833	9.5	11.8	9.9
	14.7	14.3	3.633	2.033	3.7	3.033	4.567	4.4	8.967	11.4	11.667
	14	13.1	3.467	2.967	3.9	3.967	5.167	5.133	8.333	10.9	11.633
	13.433	12.5	2.567	4.5	5.1	5.5	5.7	5.2	9.433	10.667	12.167
	11.867	10.6	2.767	3.8	4.333	4.8	4.167	5.667	9.067	10.467	12.267
	14.7	12.467	2.367	3.433	4.6	4.433	5.467	7.5	10.667	12.033	13.367
	10.233	11.7	2.3	3.133	4.067	4.133	2.767	4.5	9.333	10.2	10
NS (Ipsi)	12.633	13.333	10.267	12.433	13.233	12.9	13.133	12.533	12.867	13.8	12.567
	10.867	11.433	12.033	13.1	12.867	13.533	12.167	13.333	12	12.133	13.633
	11.367	13.2	10.833	12.767	13.8	11.467	12.067	12.567	12.333	13.4	11.033
	12.867	12.9	13.833	12.433	12.033	12.7	12.133	13.067	11.833	11.6	13.267
	13.867	13.3	11.167	10.233	13.1	13.767	12.333	11.833	13.5	12.267	13.433
	13.267	12.1	12.833	10.2	12.233	13.3	12.1	13.333	12.467	12.467	12
	13.8	13.167	11.733	13	13.067	11.3	13.4	10.7	12.4	13.433	13.1
	12.5	12.367	12.8	13.1	12.967	12.1	13.167	12.767	13.2	12.433	12.867
CFA (Contro)	13.917	12.634	11.816	11.22	12.356	12.08	12.921	13.865	12.914	12.334	11.503
	13.896	12.608	13.529	13.704	13.334	11.957	11.794	11.8	11.408	12.022	11.885

	12.621	13.64	12.233	13.774	13.627	13.332	13.188	13.728	13.864	12.736	12.935
	12.266	11.028	12.234	11.897	12.11	12.526	11.618	12.954	12.008	12.209	12.804
	12.794	13.005	11.868	12.669	11.563	12.145	12.476	11.96	13.303	12.795	11.827
	12.923	13.061	11.99	13.282	11.311	12.22	12.791	13.765	12.779	13.944	12.036
	13.843	13.038	13.334	13.478	11.332	12.972	11.487	12.315	13.696	12.696	11.051
	13.524	13.349	12.816	11.85	11.64	13.997	13.901	11.134	13.279	13.146	12.08
NS (Contro)	11.389	13.28	13.949	11.851	13.264	11.047	13.365	11.358	11.468	13.469	12.096
	11.475	11.983	11.219	13.259	11.818	11.381	13.127	12.095	13.417	13.049	11.444
	13.973	13.004	11.259	12.973	11.07	11.38	12.622	12.06	13.148	11.258	12.482
	11.443	13.544	11.544	11.807	12.781	12.597	11.848	11.181	13.005	11.378	12.53
	11.639	12.227	13.942	13.903	12.797	13.003	13.206	13.918	13.583	11.727	11.612
	12.791	11.92	12.102	11.294	12.489	12.521	11.692	12.722	12.119	11.304	13.295
	12.54	13.11	13.892	12.291	13.39	12.897	13.038	12.623	13.996	13.964	11.538
	12.197	11.644	12.047	13.453	12.173	13.648	13.346	11.738	13.34	11.639	13.223

**Figure 1B PWL -3 d**				
	CFA (Ipsi)	NS (Ipsi)	CFA (Control)	NS (Contro)
Test for normal distribution				
Shapiro-Wilk test				
W	0.9378	0.924	0.8859	0.8587
P value	0.5891	0.4633	0.2144	0.1166
Passed normality test (alpha=0.05)?	Yes	Yes	Yes	Yes
P value summary	ns	ns	ns	ns
Number of values	8	8	8	8
**Data exhibits a normal distribution**				
ANOVA summary				

F	1.189			
P value	0.3318			
P value summary	ns			
Significant diff. among means (P < 0.05)?	No			
R squared	0.113			
Dunnett's multiple comparisons test	Mean Diff.	Significant?	Summary	Adjusted P Value
CFA (Ipsi) vs. NS (Ipsi)	0.1956	No	ns	0.9719
CFA (Ipsi) vs. CFA (Control)	-0.3814	No	ns	0.8377
CFA (Ipsi) vs. NS (Contro)	0.6608	No	ns	0.5132
**Figure 1B PWL -1 d**				
	CFA (Ipsi)	NS (Ipsi)	CFA (Control)	NS (Contro)
Test for normal distribution				
Shapiro-Wilk test				
W	0.937	0.8553	0.8172	0.9031
P value	0.5818	0.1076	0.0436	0.3079
Passed normality test (alpha=0.05)?	Yes	Yes	No	Yes
P value summary	ns	ns	*	ns
Number of values	8	8	8	8
**Data does not exhibit a normal distribution, so use a non-parametric equivalent-the Kruskal-Wallis test.**				
Kruskal-Wallis test				
P value	0.9075			
Exact or approximate P value?	Approximate			
P value summary	ns			
Do the medians vary signif. (P < 0.05)?	No			
Number of groups	4			
Kruskal-Wallis statistic	0.5512			
Dunn's multiple comparisons test	Mean rank diff.	Significant?	Summary	Adjusted P Value
NS (Ipsi) vs. CFA (Ipsi)	1.25	No	ns	>0.9999
NS (Ipsi) vs. CFA (Control)	-1	No	ns	>0.9999
NS (Ipsi) vs. NS (Contro)	2.25	No	ns	>0.9999
**Figure 1B PWL 6 h**				
	CFA (Ipsi)	NS (Ipsi)	CFA	NS (Contro)

			(Control)	
Test for normal distribution				
Shapiro-Wilk test				
W	0.9438	0.9769	0.8673	0.8034
P value	0.6493	0.946	0.1417	0.0311
Passed normality test (alpha=0.05)?	Yes	Yes	Yes	No
P value summary	ns	ns	ns	*
Number of values	8	8	8	8
**Data does not exhibit a normal distribution, so use a non-parametric equivalent-the Kruskal-Wallis test.**				
Kruskal-Wallis test				
P value	0.0004			
Exact or approximate P value?	Approximate			
P value summary	***			
Do the medians vary signif. (P < 0.05)?	Yes			
Number of groups	4			
Kruskal-Wallis statistic	18.4			
Dunn's multiple comparisons test	Mean rank diff.	Significant?	Summary	Adjusted P Value
NS (Ipsi) vs. CFA (Ipsi)	13.38	Yes	*	0.0131
NS (Ipsi) vs. CFA (Control)	-4.125	No	ns	>0.9999
NS (Ipsi) vs. NS (Contro)	-3.75	No	ns	>0.9999
**Figure 1B PWL 1 d**				
	CFA (Ipsi)	NS (Ipsi)	CFA (Control)	NS (Contro)
Test for normal distribution				
Shapiro-Wilk test				
W	0.9759	0.7327	0.897	0.9491
P value	0.9398	0.0052	0.2715	0.7024
Passed normality test (alpha=0.05)?	Yes	No	Yes	Yes
P value summary	ns	**	ns	ns
Number of values	8	8	8	8
**Data does not exhibit a normal distribution, so use a non-parametric equivalent-the Kruskal-Wallis test.**				
Kruskal-Wallis test				
P value	0.0004			
Exact or approximate P value?	Approximate			
P value summary	***			

Do the medians vary signif. (P < 0.05)?	Yes			
Number of groups	4			
Kruskal-Wallis statistic	18.06			
Dunn's multiple comparisons test	Mean rank diff.	Significant?	Summary	Adjusted P Value
NS (Ipsi) vs. CFA (Ipsi)	14.13	Yes	**	0.0078
NS (Ipsi) vs. CFA (Control)	-3.625	No	ns	>0.9999
NS (Ipsi) vs. NS (Contro)	-2	No	ns	>0.9999
**Figure 1B PWL 2 d**				
	CFA (Ipsi)	NS (Ipsi)	CFA (Control)	NS (Contro)
Test for normal distribution				
Shapiro-Wilk test				
W	0.9664	0.9364	0.8636	0.9503
P value	0.8687	0.5756	0.1303	0.7144
Passed normality test (alpha=0.05)?	Yes	Yes	Yes	Yes
P value summary	ns	ns	ns	ns
Number of values	8	8	8	8
**Data exhibits a normal distribution**				
ANOVA summary				
F	266.6			
P value	<0.0001			
P value summary	****			
Significant diff. among means (P < 0.05)?	Yes			
R squared	0.9662			
Dunnett's multiple comparisons test	Mean Diff.	Significant?	Summary	Adjusted P Value
NS (Ipsi) vs. CFA (Ipsi)	8.925	Yes	****	<0.0001
NS (Ipsi) vs. CFA (Control)	0.7534	No	ns	0.1264
NS (Ipsi) vs. NS (Contro)	0.4398	No	ns	0.5049
**Figure 1B PWL 3 d**				
	CFA (Ipsi)	NS (Ipsi)	CFA (Control)	NS (Contro)
Test for normal distribution				
Shapiro-Wilk test				
W	0.9759	0.9285	0.8818	0.9159

P value	0.9398	0.5025	0.1958	0.3974
Passed normality test (alpha=0.05)?	Yes	Yes	Yes	Yes
P value summary	ns	ns	ns	ns
Number of values	8	8	8	8
**Data exhibits a normal distribution**				
ANOVA summary				
F	196.7			
P value	<0.0001			
P value summary	****			
Significant diff. among means (P < 0.05)?	Yes			
R squared	0.9547			
Dunnett's multiple comparisons test	Mean Diff.	Significant?	Summary	Adjusted P Value
NS (Ipsi) vs. CFA (Ipsi)	8.534	Yes	****	<0.0001
NS (Ipsi) vs. CFA (Control)	-0.02025	No	ns	>0.9999
NS (Ipsi) vs. NS (Contro)	0.3241	No	ns	0.788
**Figure 1B PWL 5 d**				
	CFA (Ipsi)	NS (Ipsi)	CFA (Control)	NS (Contro)
Test for normal distribution				
Shapiro-Wilk test				
W	0.9031	0.7861	0.9443	0.8168
P value	0.3083	0.0203	0.6542	0.0432
Passed normality test (alpha=0.05)?	Yes	No	Yes	No
P value summary	ns	*	ns	*
Number of values	8	8	8	8
**Data does not exhibit a normal distribution, so use a non-parametric equivalent-the Kruskal-Wallis test.**				
Kruskal-Wallis test				
P value	0.0005			
Exact or approximate P value?	Approximate			
P value summary	***			
Do the medians vary signif. (P < 0.05)?	Yes			
Number of groups	4			
Kruskal-Wallis statistic	17.94			
Dunn's multiple comparisons test	Mean rank	Significant?	Summary	Adjusted P

	diff.			Value
NS (Ipsi) vs. CFA (Ipsi)	15.75	Yes	**	0.0024
NS (Ipsi) vs. CFA (Control)	1.25	No	ns	>0.9999
NS (Ipsi) vs. NS (Contro)	-2	No	ns	>0.9999
**Figure 1B PWL 7 d**				
	CFA (Ipsi)	NS (Ipsi)	CFA (Control)	NS (Contro)
Test for normal distribution				
Shapiro-Wilk test				
W	0.7848	0.8629	0.9065	0.934
P value	0.0196	0.1284	0.3302	0.5536
Passed normality test (alpha=0.05)?	No	Yes	Yes	Yes
P value summary	*	ns	ns	ns
Number of values	8	8	8	8
**Data does not exhibit a normal distribution, so use a non-parametric equivalent-the Kruskal-Wallis test.**				
Kruskal-Wallis test				
P value	0.0004			
Exact or approximate P value?	Approximate			
P value summary	***			
Do the medians vary signif. (P < 0.05)?	Yes			
Number of groups	4			
Kruskal-Wallis statistic	18.01			
Dunn's multiple comparisons test	Mean rank diff.	Significant?	Summary	Adjusted P Value
NS (Ipsi) vs. CFA (Ipsi)	16.75	Yes	**	0.0011
NS (Ipsi) vs. CFA (Control)	-0.5	No	ns	>0.9999
NS (Ipsi) vs. NS (Contro)	2.75	No	ns	>0.9999
**Figure 1B PWL 10 d**				
	CFA (Ipsi)	NS (Ipsi)	CFA (Control)	NS (Contro)
Test for normal distribution				
Shapiro-Wilk test				
W	0.8967	0.9538	0.9269	0.904
P value	0.2695	0.7495	0.4886	0.3138
Passed normality test (alpha=0.05)?	Yes	Yes	Yes	Yes
P value summary	ns	ns	ns	ns

Number of values	8	8	8	8
**Data exhibits a normal distribution**				
ANOVA summary				
F	47.05			
P value	<0.0001			
P value summary	****			
Significant diff. among means (P < 0.05)?	Yes			
R squared	0.8345			
Holm-Sidak's multiple comparisons test	Mean Diff.	Significant?	Summary	Adjusted P Value
NS (Ipsi) vs. CFA (Ipsi)	3.288	Yes	****	<0.0001
NS (Ipsi) vs. CFA (Control)	-0.3314	No	ns	0.4324
NS (Ipsi) vs. NS (Contro)	-0.4345	No	ns	0.4324
**Figure 1B PWL 14 d**				
	CFA (Ipsi)	NS (Ipsi)	CFA (Control)	NS (Contro)
Test for normal distribution				
Shapiro-Wilk test				
W	0.9174	0.9247	0.9214	0.8226
P value	0.4094	0.469	0.4413	0.0497
Passed normality test (alpha=0.05)?	Yes	Yes	Yes	No
P value summary	ns	ns	ns	*
Number of values	8	8	8	8
**Data does not exhibit a normal distribution, so use a non-parametric equivalent-the Kruskal-Wallis test.**				
Kruskal-Wallis test				
P value	0.0057			
Exact or approximate P value?	Approximate			
P value summary	**			
Do the medians vary signif. (P < 0.05)?	Yes			
Number of groups	4			
Kruskal-Wallis statistic	12.54			
Dunn's multiple comparisons test	Mean rank diff.	Significant?	Summary	Adjusted P Value
NS (Ipsi) vs. CFA (Ipsi)	13.88	Yes	**	0.0093
NS (Ipsi) vs. CFA (Control)	-0.875	No	ns	>0.9999
NS (Ipsi) vs. NS (Contro)	5.5	No	ns	0.7229

**Figure 1B PWL 21 d**				
	CFA (Ipsi)	NS (Ipsi)	CFA (Control)	NS (Contro)
Test for normal distribution				
Shapiro-Wilk test				
W	0.9083	0.9003	0.9472	0.8979
P value	0.3423	0.2909	0.6826	0.2764
Passed normality test (alpha=0.05)?	Yes	Yes	Yes	Yes
P value summary	ns	ns	ns	ns
Number of values	8	8	8	8
**Data exhibits a normal distribution**				
Dunnett's multiple comparisons test	Mean Diff.	95.00% CI of diff.	Significant?	Summary
NS (Ipsi) vs. CFA (Ipsi)	-1.05	-2.144 to 0.04455	No	ns
NS (Ipsi) vs. CFA (Control)	-0.3275	-1.422 to 0.7669	No	ns
NS (Ipsi) vs. NS (Contro)	-0.5899	-1.684 to 0.5046	No	ns
Two-way ANOVA	Ordinary			
Alpha	0.05			
Source of Variation	% of total variation	P value	P value summary	Significant?
Interaction	10.66	<0.0001	****	Yes
Row Factor	22.06	<0.0001	****	Yes
Column Factor	54.11	<0.0001	****	Yes

Figure 1C

Western blot	Con	1/4	1	3	7	14	21
	100.644	260.076	172.740	131.869	121.869	87.619	97.619
	102.774	192.628	151.518	107.062	97.062	107.263	97.263
	101.638	257.566	220.902	155.547	125.547	114.218	104.218
	96.240	200.503	182.115	101.487	101.487	96.943	96.943
	98.705	216.258	229.990	140.147	140.147	106.362	110.362

Figure 1C							
	Con	1/4	1	3	7	14	21
Test for normal distribution							
Shapiro-Wilk test							
W	0.9587	0.8554	0.9278	0.9364	0.9338	0.9522	0.8053
P value	0.7991	0.2123	0.5813	0.6403	0.6223	0.7529	0.0894
Passed normality test (alpha=0.05)?	Yes	Yes	Yes	Yes	Yes	Yes	Yes
P value summary	ns	ns	ns	ns	ns	ns	ns
Number of values	5	5	5	5	5	5	5
**Data exhibits a normal distribution**							
ANOVA summary							
F	28.59						
P value	<0.0001						
P value summary	****						
Significant diff. among means (P < 0.05)?	Yes						
R squared	0.8597						
Dunnett's multiple comparisons test	Mean Diff.	95.00% CI of diff.	Significant?	Summary	Adjusted P Value		
Con vs. 1/4	-125.4	-161.6 to -89.17	Yes	****	<0.0001		
Con vs. 1	-91.45	-127.7 to -55.21	Yes	****	<0.0001		
Con vs. 3	-27.22	-63.46 to 9.017	No	ns	0.199		
Con vs. 7	-17.22	-53.46 to 19.02	No	ns	0.6198		
Con vs. 14	-2.481	-38.72 to 33.76	No	ns	0.9997		
Con vs. 21	-1.281	-37.52 to 34.96	No	ns	0.9999		

Figure 1D

qPCR	Con	1/4	1	3	7	14	21
	1.015	5.044	3.959	2.943	2.643	2.451	2.451
	1.011	4.747	3.632	2.506	2.306	2.897	2.197
	0.978	4.649	3.128	2.743	2.743	2.913	1.913
	0.973	4.564	4.340	3.563	3.563	1.860	1.860
	1.023	4.313	4.314	3.903	3.203	2.308	1.608
Figure 1D							
	Con	1/4	1	3	7	14	21
Test for normal distribution							
Shapiro-Wilk test							
W	0.8482	0.9867	0.9111	0.9336	0.9667	0.9136	0.9735
P value	0.189	0.9668	0.4741	0.6208	0.8536	0.4897	0.8975
Passed normality test (alpha=0.05)?	Yes	Yes	Yes	Yes	Yes	Yes	Yes
P value summary	ns	ns	ns	ns	ns	ns	ns
Number of values	5	5	5	5	5	5	5
**Data exhibits a normal distribution**							
Dunnett's multiple comparisons test	Mean Diff.	95.00% CI of diff.	Significant?	Summary	Adjusted P Value		
Con vs. 1/4	-3.663	-4.383 to -2.944	Yes	****	<0.0001		
Con vs. 1	-2.875	-3.594 to -2.155	Yes	****	<0.0001		
Con vs. 3	-2.132	-2.851 to -1.412	Yes	****	<0.0001		
Con vs. 7	-1.892	-2.611 to -1.172	Yes	****	<0.0001		
Con vs. 14	-1.486	-2.205 to -0.7667	Yes	****	<0.0001		
Con vs. 21	-1.006	-1.725 to -0.2867	Yes	**	0.0036		

Figure 1E

Western blot	Con	1/4	1	3	7	14	21
	97.46	59.76	59.66	68.41	80.04	97.39	96.63
	112.85	40.83	53.81	60.85	60.27	87.27	116.02
	93.84	44.25	69.10	64.86	69.06	89.88	82.41
	102.35	37.09	47.52	57.28	69.15	85.63	106.49
	93.50	45.69	54.15	74.09	69.86	75.85	96.16
Figure 1E							
	Con	1/4	1	3	7	14	21
Test for normal distribution							
Shapiro-Wilk test							
W	0.8645	0.8866	0.9491	0.9876	0.9043	0.9714	0.9738
P value	0.2448	0.3404	0.7305	0.9707	0.4343	0.8842	0.8989
Passed normality test (alpha=0.05)?	Yes	Yes	Yes	Yes	Yes	Yes	Yes
P value summary	ns	ns	ns	ns	ns	ns	ns
Number of values	5	5	5	5	5	5	5
**Data exhibits a normal distribution**							
Dunnett's multiple comparisons test	Mean Diff.	95.00% CI of diff.	Significant?	Summary	Adjusted P Value		
Con vs. 1/4	54.48	39.66 to 69.29	Yes	****	<0.0001		
Con vs. 1	43.15	28.34 to 57.97	Yes	****	<0.0001		
Con vs. 3	34.9	20.09 to 49.72	Yes	****	<0.0001		
Con vs. 7	30.32	15.51 to 45.14	Yes	****	<0.0001		
Con vs. 14	12.8	-2.018 to 27.61	No	ns	0.1102		
Con vs. 21	0.458	-14.36 to 15.27	No	ns	0.9999		

Figure 1J

Positive neurons	Con	1/4	3	7
	13.2	58.7	37	20.8
	11.6	61.4	37.2	19
	10.5	69.6	38.8	21.3

	13.5	61.8	40	20.2
	13.5	65.9	37.8	21.5
Figure 1J				
	Con	1/4	3	7
Test for normal distribution				
Shapiro-Wilk test				
W	0.8248	0.9467	0.9149	0.9165
P value	0.1272	0.7136	0.4974	0.5077
Passed normality test (alpha=0.05)?	Yes	Yes	Yes	Yes
P value summary	ns	ns	ns	ns
Number of values	5	5	5	5
**Data exhibits a normal distribution**				
Dunnett's multiple comparisons test	Mean Diff.	Significant?	Summary	Adjusted P Value
Con vs. 1/4	-51.02	Yes	****	<0.0001
Con vs. 3	-25.7	Yes	****	<0.0001
Con vs. 7	-8.1	Yes	***	0.0002

Figure 1K

Western blot	Con	1/4	1	3	7	14	21
	102.78	157.14	266.56	146.67	134.30	126.67	101.70
	99.79	165.17	279.07	172.24	113.24	117.24	95.73
	98.69	147.65	284.98	137.82	121.29	113.82	94.73
	98.36	128.05	275.84	161.35	142.61	102.35	113.76
	100.38	140.09	264.49	157.09	123.35	121.09	93.59
Figure 1K							
	Con	1/4	1	3	7	14	21
Test for normal distribution							
Shapiro-Wilk test							
W	0.9086	0.9885	0.9374	0.988	0.9648	0.9668	0.8152
P value	0.4593	0.974	0.648	0.9724	0.8408	0.8546	0.1071
Passed normality test (alpha=0.05)?	Yes	Yes	Yes	Yes	Yes	Yes	Yes
P value summary	ns	ns	ns	ns	ns	ns	ns
Number of values	5	5	5	5	5	5	5
**Data exhibits a normal distribution**							
Dunnett's multiple comparisons test	Mean Diff.	95.00% CI of diff.	Significant?	Summary	Adjusted P Value		

Con vs. 1/4	-47.62	-65.49 to -29.75	Yes	****	<0.0001		
Con vs. 1	-174.2	-192.1 to -156.3	Yes	****	<0.0001		
Con vs. 3	-55.03	-72.90 to -37.17	Yes	****	<0.0001		
Con vs. 7	-26.96	-44.82 to -9.087	Yes	**	0.0016		
Con vs. 14	-16.23	-34.10 to 1.635	No	ns	0.0857		
Con vs. 21	0.09886	-17.77 to 17.97	No	ns	>0.9999		

Figure 1L

qPCR	Con	1/4	1	3	7	14	21
	0.98	3.94	6.46	4.44	2.95	2.84	1.65
	1.03	5.25	5.13	3.01	3.40	2.01	1.80
	0.99	4.15	6.63	4.24	4.41	2.44	1.31
	1.02	5.06	5.84	5.06	3.36	2.26	1.26
	0.98	5.31	4.81	4.40	2.81	1.80	0.91
Figure 1L							
	Con	1/4	1	3	7	14	21
Test for normal distribution							
Shapiro-Wilk test							
W	0.8476	0.825	0.9179	0.8835	0.8736	0.9625	0.9605
P value	0.1869	0.1276	0.5167	0.3257	0.2811	0.8249	0.8118
Passed normality test (alpha=0.05)?	Yes	Yes	Yes	Yes	Yes	Yes	Yes
P value summary	ns	ns	ns	ns	ns	ns	ns
Number of values	5	5	5	5	5	5	5
**Data exhibits a normal distribution**							
Dunnett's multiple comparisons test	Mean Diff.	95.00% CI of diff.	Significant?	Summary	Adjusted P Value		
Con vs. 1/4	-3.743	-4.733 to -2.754	Yes	****	<0.0001		

Con vs. 1	-4.775	-5.764 to -3.785	Yes	****	<0.0001		
Con vs. 3	-3.232	-4.221 to -2.242	Yes	****	<0.0001		
Con vs. 7	-2.386	-3.376 to -1.396	Yes	****	<0.0001		
Con vs. 14	-1.272	-2.261 to -0.2818	Yes	**	0.0079		
Con vs. 21	-0.3858	-1.376 to 0.6040	No	ns	0.7794		

Figure 1O

Western blot	Con	1/4	1	3	7	14	21
	101.28	47.42	47.83	55.38	76.67	76.64	86.59
	105.95	41.55	46.50	49.43	81.44	82.44	118.82
	99.22	34.59	49.37	58.79	67.50	91.25	88.00
	94.20	41.64	53.89	60.47	48.84	65.36	104.08
	99.35	48.81	56.16	59.28	71.08	81.28	98.20
Figure 1O							
	Con	1/4	1	3	7	14	21
Test for normal distribution							
Shapiro-Wilk test							
W	0.9621	0.9244	0.919	0.8586	0.9102	0.9673	0.9222
P value	0.8225	0.5585	0.5234	0.2233	0.4689	0.8577	0.5445
Passed normality test (alpha=0.05)?	Yes	Yes	Yes	Yes	Yes	Yes	Yes
P value summary	ns	ns	ns	ns	ns	ns	ns
Number of values	5	5	5	5	5	5	5
**Data exhibits a normal distribution**							
Dunnett's multiple comparisons test	Mean Diff.	95.00% CI of diff.	Significant?	Summary	Adjusted P Value		
Con vs. 1/4	57.2	42.50 to 71.89	Yes	****	<0.0001		
Con vs. 1	49.25	34.56 to 63.94	Yes	****	<0.0001		

Con vs. 3	43.33	28.64 to 58.02	Yes	****	<0.0001		
Con vs. 7	30.89	16.20 to 45.59	Yes	****	<0.0001		
Con vs. 14	20.61	5.913 to 35.30	Yes	**	0.0035		
Con vs. 21	0.862	-13.83 to 15.56	No	ns	0.9998		

Figure 1U

Positive neurons	Naive	1	3	10	Naive
	15.2	59.7	41	30.8	15.2
	14.6	49.4	33.2	25	14.6
	13.3	64.6	32.8	33.3	13.3
	11.5	60.8	35	22.2	11.5
	16.5	45.9	19.8	25.5	16.5
Figure 1U					
	Naive	1	3	10	
Test for normal distribution					
Shapiro-Wilk test					
W	0.9831	0.8994	0.8898	0.9308	
P value	0.9504	0.4063	0.3559	0.6017	
Passed normality test (alpha=0.05)?	Yes	Yes	Yes	Yes	
P value summary	ns	ns	ns	ns	
Number of values	5	5	5	5	
**Data exhibits a normal distribution**					
Dunnett's multiple comparisons test	Mean Diff.	95.00% CI of diff.	Significant ?	Summary	Adjusted P Value
Naive vs. 1	-41.86	-51.85 to -31.87	Yes	****	<0.0001
Naive vs. 3	-18.14	-28.13 to -8.151	Yes	***	0.0007
Naive vs. 10	-13.14	-23.13 to -3.151	Yes	**	0.0096

Figure 3A

Fig 5A PWT	BL	1/4	1	3	5	7	10
Naive	21.368	21.368	21.368	21.368	21.368	21.368	21.368
	23.368	25.368	25.368	25.368	25.368	25.368	25.368
	21.48	25.368	25.368	25.368	25.368	24.48	25.368
	25.368	25.368	22.48	25.368	25.368	25.368	25.368

	25.368	25.368	25.368	25.368	25.368	25.368	25.368
	28.368	28.368	28.368	28.368	28.368	28.368	28.368
CFA	19.368	2.933	5.313	5.313	5.313	8.058	13.48
	25.368	2.933	5.313	5.313	5.313	8.058	11.48
	25.368	2.933	5.313	5.313	5.313	8.058	8.058
	25.368	2.933	5.313	2.933	5.313	5.313	8.058
	21.48	2.933	2.933	5.313	5.313	5.313	12.48
	25.368	5.313	2.933	2.933	2.933	8.058	8.058
CFA + veh	20.368	2.933	5.313	8.058	5.313	7.058	12.48
	25.368	5.313	5.313	5.313	5.313	8.058	12.48
	25.368	5.313	5.313	5.313	5.313	8.058	12.48
	25.368	2.933	2.933	5.313	2.933	8.058	8.058
	22.48	2.933	2.933	2.933	5.313	5.313	12.48
	25.368	2.933	2.933	2.933	2.933	8.058	8.058
CFA + GSK 5µg	25.368	6.058	3.313	6.058	6.058	9.058	10.48
	25.368	6.058	10.48	7.058	6.058	10.058	9.48
	25.368	6.058	10.48	9.48	10.48	6.058	11.48
	25.368	10.48	6.058	8.48	10.48	10.48	12.48
	23.48	6.058	6.058	7.058	6.058	10.48	12.48
	25.368	6.058	6.058	6.058	6.058	10.48	15.368
CFA + GSK 25µg	25.368	10.48	10.48	10.48	11.48	10.48	14.48
	22.48	10.48	10.48	10.48	9.48	10.48	13.48
	25.368	10.48	10.48	10.48	8.48	10.48	18.368
	25.368	10.48	10.48	10.48	12.48	10.48	17.368
	25.368	6.058	10.48	10.48	10.48	16.368	13.48
	25.368	6.058	6.058	6.058	10.48	15.368	16.368
CFA + GSK 50µg	25.368	12.268	13.868	12.368	15.368	15.368	15.368
	25.368	10.38	13.868	10.48	9.48	16.368	20.368
	25.368	11.38	8.98	10.48	16.368	17.368	19.368
	25.368	14.38	13.98	16.48	16.48	16.48	16.48
	25.368	13.268	13.98	16.368	15.368	18.368	18.368
	25.368	14.268	12.868	15.368	14.368	17.368	17.368

**Figure 3A PWT -3 d**							
	Naive	CFA	CFA + veh	CFA + GSK 5µg	CFA + GSK 25µg	CFA + GSK 50µg	
Test for normal distribution							
Shapiro-Wilk test							
W	0.9191	0.7035	0.7055	0.4961	0.4961	Invalid input	

						data	
P value	0.4986	0.0067	0.0071	<0.0001	<0.0001		
Passed normality test (alpha=0.05)?	Yes	No	No	No	No		
P value summary	ns	**	**	****	****		
Number of values	6	6	6	6	6	6	
**Data does not exhibit a normal distribution, so use a non-parametric equivalent-the Kruskal-Wallis test**							
Kruskal-Wallis test							
P value	0.6586						
Exact or approximate P value?	Approximate						
P value summary	ns						
Do the medians vary signif. (P < 0.05)?	No						
Number of groups	6						
Kruskal-Wallis statistic	3.269						
Dunn's multiple comparisons test	Mean rank diff.	Significant?	Summary	Adjusted P Value			
CFA vs. Naive	-0.1667	No	ns	>0.9999			
CFA vs. CFA + veh	-0.5	No	ns	>0.9999			
CFA vs. CFA + GSK 5µg	-4.333	No	ns	>0.9999			
CFA vs. CFA + GSK 25µg	-3.917	No	ns	>0.9999			
CFA vs. CFA + GSK 50µg	-6.583	No	ns	0.8521			
**Figure 3A PWT 6 h**							
	Naive	CFA	CFA + veh	CFA + GSK 5µg	CFA + GSK 25µg	CFA + GSK 50µg	
Test for normal distribution							
Shapiro-Wilk test							
W	0.8157	0.4961	0.6399	0.4961	0.6399	0.9336	
P value	0.081	<0.0001	0.0014	<0.0001	0.0014	0.6078	
Passed normality test (alpha=0.05)?	Yes	No	No	No	No	Yes	
P value summary	ns	****	**	****	**	ns	
Number of values	6	6	6	6	6	6	
**Data does not exhibit a normal distribution, so use a non-parametric equivalent-the Kruskal-Wallis test.**							
Kruskal-Wallis test							
P value	<0.0001						

Exact or approximate P value?	Approximate						
P value summary	****						
Do the medians vary signif. (P < 0.05)?	Yes						
Number of groups	6						
Kruskal-Wallis statistic	32.59						
Dunn's multiple comparisons test	Mean rank diff.	Significant?	Summary	Adjusted P Value			
CFA vs. Naive	-27.5	Yes	****	<0.0001			
CFA vs. CFA + veh	-1	No	ns	>0.9999			
CFA vs. CFA + GSK 5µg	-11.17	No	ns	0.3136			
CFA vs. CFA + GSK 25µg	-14.67	No	ns	0.0725			
CFA vs. CFA + GSK 50µg	-20.67	Yes	**	0.0029			
**Figure 3A PWT 1 d**							
	Naive	CFA	CFA + veh	CFA + GSK 5µg	CFA + GSK 25µg	CFA + GSK 50µg	
Test for normal distribution							
Shapiro-Wilk test							
W	0.9153	0.6399	0.6827	0.846	0.4961	0.6356	
P value	0.4725	0.0014	0.004	0.1461	<0.0001	0.0012	
Passed normality test (alpha=0.05)?	Yes	No	No	Yes	No	No	
P value summary	ns	**	**	ns	****	**	
Number of values	6	6	6	6	6	6	
**Data does not exhibit a normal distribution, so use a non-parametric equivalent-the Kruskal-Wallis test.**							
Kruskal-Wallis test							
P value	<0.0001						
Exact or approximate P value?	Approximate						
P value summary	****						
Do the medians vary signif. (P < 0.05)?	Yes						
Number of groups	6						
Kruskal-Wallis statistic	30.83						
Dunn's multiple comparisons test	Mean rank	Significant?	Summary	Adjusted P			

	diff.			Value			
CFA vs. Naive	-25.83	Yes	****	<0.0001			
CFA vs. CFA + veh	1.167	No	ns	>0.9999			
CFA vs. CFA + GSK 5µg	-8.417	No	ns	0.812			
CFA vs. CFA + GSK 25µg	-13.25	No	ns	0.1393			
CFA vs. CFA + GSK 50µg	-18.67	Yes	**	0.0097			
**Figure 3A PWT 3 d**							
	Naive	CFA	CFA + veh	CFA + GSK 5µg	CFA + GSK 25µg	CFA + GSK 50µg	
Test for normal distribution							
Shapiro-Wilk test							
W	0.8157	0.6399	0.8637	0.8899	0.4961	0.8325	
P value	0.081	0.0014	0.2022	0.3177	<0.0001	0.1128	
Passed normality test (alpha=0.05)?	Yes	No	Yes	Yes	No	Yes	
P value summary	ns	**	ns	ns	****	ns	
Number of values	6	6	6	6	6	6	
**Data does not exhibit a normal distribution, so use a non-parametric equivalent-the Kruskal-Wallis test.**							
Kruskal-Wallis test							
P value	<0.0001						
Exact or approximate P value?	Approximate						
P value summary	****						
Do the medians vary signif. (P < 0.05)?	Yes						
Number of groups	6						
Kruskal-Wallis statistic	31.76						
Dunn's multiple comparisons test	Mean rank diff.	Significant?	Summary	Adjusted P Value			
CFA vs. Naive	-27.33	Yes	****	<0.0001			
CFA vs. CFA + veh	-1.5	No	ns	>0.9999			
CFA vs. CFA + GSK 5µg	-9.5	No	ns	0.5754			
CFA vs. CFA + GSK 25µg	-15.17	No	ns	0.0594			
CFA vs. CFA + GSK 50µg	-20.5	Yes	**	0.0034			
**Figure 3A PWT 5 d**							
	Naive	CFA	CFA + veh	CFA + GSK	CFA + GSK	CFA + GSK	

				5µg	25µg	50µg	
Test for normal distribution							
Shapiro-Wilk test							
W	0.8157	0.4961	0.6399	0.6399	0.9818	0.7514	
P value	0.081	<0.0001	0.0014	0.0014	0.96	0.0206	
Passed normality test (alpha=0.05)?	Yes	No	No	No	Yes	No	
P value summary	ns	****	**	**	ns	*	
Number of values	6	6	6	6	6	6	
**Data does not exhibit a normal distribution, so use a non-parametric equivalent-the Kruskal-Wallis test.**							
Kruskal-Wallis test							
P value	<0.0001						
Exact or approximate P value?	Approximate						
P value summary	****						
Do the medians vary signif. (P < 0.05)?	Yes						
Number of groups	6						
Kruskal-Wallis statistic	32.35						
Dunn's multiple comparisons test	Mean rank diff.	Significant?	Summary	Adjusted P Value			
CFA vs. Naive	-26.5	Yes	****	<0.0001			
CFA vs. CFA + veh	1	No	ns	>0.9999			
CFA vs. CFA + GSK 5µg	-9.833	No	ns	0.5123			
CFA vs. CFA + GSK 25µg	-14.25	No	ns	0.0898			
CFA vs. CFA + GSK 50µg	-19.42	Yes	**	0.0063			
**Figure 3A PWT 7 d**							
	Naive	CFA	CFA + veh	CFA + GSK 5µg	CFA + GSK 25µg	CFA + GSK 50µg	
Test for normal distribution							
Shapiro-Wilk test							
W	0.8956	0.6399	0.6752	0.7071	0.6786	0.9697	
P value	0.3484	0.0014	0.0034	0.0073	0.0036	0.8905	
Passed normality test (alpha=0.05)?	Yes	No	No	No	No	Yes	
P value summary	ns	**	**	**	**	ns	
Number of values	6	6	6	6	6	6	
**Data does not exhibit a normal distribution, so use a non-parametric equivalent-the Kruskal-Wallis**							

test.							
Kruskal-Wallis test							
P value	<0.0001						
Exact or approximate P value?	Approximate						
P value summary	****						
Do the medians vary signif. (P < 0.05)?	Yes						
Number of groups	6						
Kruskal-Wallis statistic	31.48						
Dunn's multiple comparisons test	Mean rank diff.	Significant?	Summary	Adjusted P Value			
CFA vs. Naive	-26.5	Yes	****	<0.0001			
CFA vs. CFA + veh	-0.5	No	ns	>0.9999			
CFA vs. CFA + GSK 5µg	-8	No	ns	0.9206			
CFA vs. CFA + GSK 25µg	-13.83	No	ns	0.1082			
CFA vs. CFA + GSK 50µg	-20.17	Yes	**	0.0041			
**Figure 3A PWT 10 d**							
	Naive	CFA	CFA + veh	CFA + GSK 5µg	CFA + GSK 25µg	CFA + GSK 50µg	
Test for normal distribution							
Shapiro-Wilk test							
W	0.8157	0.8074	0.6399	0.9492	0.8958	0.9854	
P value	0.081	0.0684	0.0014	0.7338	0.3498	0.975	
Passed normality test (alpha=0.05)?	Yes	Yes	No	Yes	Yes	Yes	
P value summary	ns	ns	**	ns	ns	ns	
Number of values	6	6	6	6	6	6	
**Data does not exhibit a normal distribution, so use a non-parametric equivalent-the Kruskal-Wallis test.**							
Kruskal-Wallis test							
P value	<0.0001						
Exact or approximate P value?	Approximate						
P value summary	****						
Do the medians vary signif. (P < 0.05)?	Yes						
Number of groups	6						
Kruskal-Wallis statistic	29.16						

Dunn's multiple comparisons test	Mean rank diff.	Significant?	Summary	Adjusted P Value			
CFA vs. Naive	-25.42	Yes	***	0.0001			
CFA vs. CFA + veh	-1.583	No	ns	>0.9999			
CFA vs. CFA + GSK 5µg	-3.417	No	ns	>0.9999			
CFA vs. CFA + GSK 25µg	-13.92	No	ns	0.1067			
CFA vs. CFA + GSK 50µg	-18.17	Yes	*	0.0133			
**Figure 3A** PWT CFA vs Baseline							
	BL	1/4	1	3	5	7	10
Test for normal distribution							
Shapiro-Wilk test							
W	0.7035	0.4961	0.6399	0.6399	0.4961	0.6399	0.8074
P value	0.0067	<0.0001	0.0014	0.0014	<0.0001	0.0014	0.0684
Passed normality test (alpha=0.05)?	No	No	No	No	No	No	Yes
P value summary	**	****	**	**	****	**	ns
Number of values	6	6	6	6	6	6	6
**Data does not exhibit a normal distribution, so use a non-parametric equivalent-the Kruskal-Wallis test.**							
Kruskal-Wallis test							
P value	<0.0001						
Exact or approximate P value?	Approximate						
P value summary	****						
Do the medians vary signif. (P < 0.05)?	Yes						
Number of groups	7						
Kruskal-Wallis statistic	34.11						
Dunn's multiple comparisons test	Mean rank diff.	Significant?	Summary	Adjusted P Value			
BL vs. 1/4	31.83	Yes	####	<0.0001			
BL vs. 1	25.33	Yes	##	0.0012			
BL vs. 3	25.33	Yes	##	0.0012			
BL vs. 5	23.17	Yes	##	0.0041			
BL vs. 7	13.33	No	ns	0.3027			
BL vs. 10	7	No	ns	>0.9999			

Two-way ANOVA	Ordinary						
Alpha	0.05						
Source of Variation	% of total variation	P value	P value summary	Significant?			
Interaction	9.282	<0.0001	****	Yes			
Row Factor	34.87	<0.0001	****	Yes			
Column Factor	51.02	<0.0001	****	Yes			

Figure 3B

Figure 3B PWL	BL	1/4	1	3	5	7	10
Naive	11.781	12.067	11.2	12.5	12.867	13.233	11.4
	12.207	13.3	10.967	11.633	11.867	12.167	12.567
	11.768	12.233	13.5	12.833	12.567	11.967	12.133
	12.626	12.333	11.433	12.767	12.467	11.8	12.233
	12.433	11.5	13.467	12.233	11.7	12.6	11.633
	12.636	12.9	12.3	12.4	12.133	12.5	12.733
CFA	12.089	2.85	3.35	3.05	4.683	5.833	7.733
	11.759	1.417	1.917	3.717	4.417	3.533	9.833
	11.8	3.583	4.083	3.817	4.65	7.333	8.467
	12.011	2.417	2.917	5.017	4.917	4.933	8.6
	12.69	3.483	3.983	4.017	3.383	5.333	8.667
	12.641	2.483	2.983	4.017	4.383	5.2	8.667
CFA + veh	12.198	3.15	3.45	3.717	4.35	4.3	9.067
	12.504	2.95	3.25	4.95	4.25	4.933	8.367
	12.589	2.95	3.25	3.65	4.883	5.533	8.933
	12.617	3.617	3.917	3.75	4.417	5.1	8.533
	11.737	2.85	3.15	4.583	5.083	5.467	8.3
	12.757	2.783	3.083	4.217	4.517	5.833	8.4
CFA + GSK 5µg	12.773	4.057	2.523	5.123	6.423	4.657	9.957
	11.39	3.923	6.223	4.723	5.957	5.657	9.957
	11.673	4.69	3.69	4.323	4.157	3.723	7.857
	13.383	3.89	4.357	4.857	4.523	7.357	7.423
	13.396	3.523	3.09	4.223	4.757	7.023	11.19
	12.503	3.123	4.757	5.023	4.69	7.857	9.99
CFA + GSK 25µg	12.384	4.62	4.687	4.653	5.953	6.303	10.203
	12.494	4.687	4.387	5.387	3.453	7.137	9.07
	10.325	5.387	6.187	5.787	7.887	6.837	8.97
	14.15	5.22	3.653	6.053	5.42	6.87	10.237
	11.893	4.787	5.987	6.187	6.52	7.237	10.137

	12.082	3.92	5.353	6.32	6.253	6.67	10.37
CFA + GSK 50μg	11.875	3.783	5.35	6.15	7.45	6.333	9.833
	11.44	6.717	6.883	7.983	7.583	9.167	10.7
	13.609	5.45	6.983	6.05	6.517	7.867	8.6
	10.929	6.15	8.017	8.183	8.35	7.9	11.867
	14.579	6.817	6.683	7.283	7.617	8.267	10.767
	12.71	6.75	7.917	7.217	7.383	7.7	11

**Figure 3B PWL -3 d**							
	Naive	CFA	CFA + veh	CFA + GSK 5μg	CFA + GSK 25μg	CFA + GSK 50μg	
Test for normal distribution							
Shapiro-Wilk test							
W	0.8547	0.8536	0.8695	0.901	0.9342	0.9584	
P value	0.1718	0.1681	0.2241	0.3797	0.613	0.8077	
Passed normality test (alpha=0.05)?	Yes	Yes	Yes	Yes	Yes	Yes	
P value summary	ns	ns	ns	ns	ns	ns	
Number of values	6	6	6	6	6	6	
**Data exhibits a normal distribution**							
Kruskal-Wallis test							
P value	0.9688						
Exact or approximate P value?	Approximate						
P value summary	ns						
Do the medians vary signif. (P < 0.05)?	No						
Number of groups	6						
Kruskal-Wallis statistic	0.9189						
Dunn's multiple comparisons test	Mean rank diff.	Significant?	Summary	Adjusted P Value			
CFA vs. Naive	-0.6667	No	ns	>0.9999			
CFA vs. CFA + veh	-3.5	No	ns	>0.9999			
CFA vs. CFA + GSK 5 ug	-4.167	No	ns	>0.9999			
CFA vs. CFA + GSK 25 ug	0	No	ns	>0.9999			

CFA vs. CFA + GSK 50 ug	-2.667	No	ns	>0.9999			
**Figure 3B PWL 6 h**							
	Naive	CFA	CFA + veh	CFA + GSK 5µg	CFA + GSK 25µg	CFA + GSK 50µg	
Test for normal distribution							
Shapiro-Wilk test							
W	0.9739	0.9325	0.8318	0.9694	0.9402	0.8035	
P value	0.9176	0.5997	0.1114	0.8881	0.6607	0.0631	
Passed normality test (alpha=0.05)?	Yes	Yes	Yes	Yes	Yes	Yes	
P value summary	ns	ns	ns	ns	ns	ns	
Number of values	6	6	6	6	6	6	
**Data exhibits a normal distribution**							
ANOVA summary							
F	151.6						
P value	<0.0001						
P value summary	****						
Significant diff. among means (P < 0.05)?	Yes						
R squared	0.9619						
Dunnett's multiple comparisons test	Mean Diff.	95.00% CI of diff.	Significant?	Summary	Adjusted P Value		
CFA vs. Naive	-9.683	-10.78 to -8.586	Yes	****	<0.0001		
CFA vs. CFA + veh	-0.3445	-1.442 to 0.7529	No	ns	0.8713		
CFA vs. CFA + GSK 5µg	-1.162	-2.260 to -0.06475	Yes	*	0.035		
CFA vs. CFA + GSK 25µg	-2.065	-3.162 to -0.9672	Yes	***	0.0001		
CFA vs. CFA + GSK	-3.239	-4.336	Yes	****	<0.0001		

50µg		to -2.142					
**Figure 3B PWL 1 d**							
	Naive	CFA	CFA + veh	CFA + GSK 5µg	CFA + GSK 25µg	CFA + GSK 50µg	
Test for normal distribution							
Shapiro-Wilk test							
W	0.8573	0.9325	0.8318	0.9728	0.9545	0.9103	
P value	0.1801	0.5997	0.1114	0.9108	0.7769	0.4381	
Passed normality test (alpha=0.05)?	Yes	Yes	Yes	Yes	Yes	Yes	
P value summary	ns	ns	ns	ns	ns	ns	
Number of values	6	6	6	6	6	6	
**Data exhibits a normal distribution**							
ANOVA summary							
F	73.74						
P value	<0.0001						
P value summary	****						
Significant diff. among means (P < 0.05)?	Yes						
R squared	0.9248						
Dunnett's multiple comparisons test	Mean Diff.	95.00% CI of diff.	Significant?	Summary	Adjusted P Value		
CFA vs. Naive	-8.939	-10.47 to -7.404	Yes	****	<0.0001		
CFA vs. CFA + veh	-0.1445	-1.680 to 1.391	No	ns	0.9997		
CFA vs. CFA + GSK 5µg	-0.9012	-2.436 to 0.6340	No	ns	0.4663		
CFA vs. CFA + GSK 25µg	-1.837	-3.372 to -0.3017	Yes	*	0.0131		
CFA vs. CFA + GSK 50µg	-3.767	-5.302 to -2.232	Yes	****	<0.0001		

**Figure 3B PWL 3 d**							
	Naive	CFA	CFA + veh	CFA + GSK 5µg	CFA + GSK 25µg	CFA + GSK 50µg	
Test for normal distribution							
Shapiro-Wilk test							
W	0.9128	0.9206	0.8779	0.9146	0.897	0.8963	
P value	0.4548	0.5094	0.2596	0.4671	0.3566	0.3527	
Passed normality test (alpha=0.05)?	Yes	Yes	Yes	Yes	Yes	Yes	
P value summary	ns	ns	ns	ns	ns	ns	
Number of values	6	6	6	6	6	6	
**Data exhibits a normal distribution**							
ANOVA summary							
F	166.3						
P value	<0.0001						
P value summary	****						
Significant diff. among means (P < 0.05)?	Yes						
R squared	0.9652						
Dunnett's multiple comparisons test	Mean Diff.	95.00% CI of diff.	Significant?	Summary	Adjusted P Value		
CFA vs. Naive	-8.455	-9.385 to -7.526	Yes	****	<0.0001		
CFA vs. CFA + veh	-0.2053	-1.135 to 0.7243	No	ns	0.965		
CFA vs. CFA + GSK 5µg	-0.7728	-1.702 to 0.1568	No	ns	0.129		
CFA vs. CFA + GSK 25µg	-1.792	-2.722 to -0.8623	Yes	****	<0.0001		
CFA vs. CFA + GSK 50µg	-3.205	-4.135 to -2.276	Yes	****	<0.0001		

**Figure 3B PWL 5 d**							
	Naive	CFA	CFA + veh	CFA + GSK 5µg	CFA + GSK 25µg	CFA + GSK 50µg	
Test for normal distribution							
Shapiro-Wilk test							
W	0.9634	0.8241	0.8969	0.8719	0.9508	0.9151	
P value	0.8453	0.0958	0.3557	0.2337	0.7469	0.4707	
Passed normality test (alpha=0.05)?	Yes	Yes	Yes	Yes	Yes	Yes	
P value summary	ns	ns	ns	ns	ns	ns	
Number of values	6	6	6	6	6	6	
**Data exhibits a normal distribution**							
ANOVA summary							
F	82.81						
P value	<0.0001						
P value summary	****						
Significant diff. among means (P < 0.05)?	Yes						
R squared	0.9324						
Dunnett's multiple comparisons test	Mean Diff.	95.00% CI of diff.	Significant?	Summary	Adjusted P Value		
CFA vs. Naive	-7.861	-9.094 to -6.629	Yes	****	<0.0001		
CFA vs. CFA + veh	-0.1778	-1.410 to 1.055	No	ns	0.9945		
CFA vs. CFA + GSK 5µg	-0.679	-1.911 to 0.5534	No	ns	0.4595		
CFA vs. CFA + GSK 25µg	-1.509	-2.741 to -0.2764	Yes	*	0.0121		
CFA vs. CFA + GSK 50µg	-3.078	-4.310 to -1.845	Yes	****	<0.0001		
**Figure 3B PWL 7 d**							
	Naive	CFA	CFA +	CFA +	CFA +	CFA +	

			veh	GSK 5µg	GSK 25µg	GSK 50µg	
Test for normal distribution							
Shapiro-Wilk test							
W	0.9484	0.9567	0.9551	0.9313	0.9567	0.9304	
P value	0.7272	0.7942	0.7812	0.59	0.7941	0.5829	
Passed normality test (alpha=0.05)?	Yes	Yes	Yes	Yes	Yes	Yes	
P value summary	ns	ns	ns	ns	ns	ns	
Number of values	6	6	6	6	6	6	
**Data exhibits a normal distribution**							
ANOVA summary							
F	45.26						
P value	<0.0001						
P value summary	****						
Significant diff. among means (P < 0.05)?	Yes						
R squared	0.8829						
Dunnett's multiple comparisons test	Mean Diff.	95.00% CI of diff.	Significant?	Summary	Adjusted P Value		
CFA vs. Naive	-7.017	-8.518 to -5.516	Yes	****	<0.0001		
CFA vs. CFA + veh	0.1665	-1.334 to 1.667	No	ns	0.9983		
CFA vs. CFA + GSK 5µg	-0.6848	-2.185 to 0.8157	No	ns	0.6295		
CFA vs. CFA + GSK 25µg	-1.482	-2.982 to 0.01905	No	ns	0.0539		
CFA vs. CFA + GSK 50µg	-2.512	-4.012 to -1.011	Yes	***	0.0005		
**Figure 3B PWL 10 d**							
	Naive	CFA	CFA + veh	CFA + GSK 5µg	CFA + GSK 25µg	CFA + GSK 50µg	

Test for normal distribution							
Shapiro-Wilk test							
W	0.9469	0.8763	0.851	0.8722	0.7579	0.9441	
P value	0.7148	0.2524	0.1603	0.2353	0.0238	0.6921	
Passed normality test (alpha=0.05)?	Yes	Yes	Yes	Yes	No	Yes	
P value summary	ns	ns	ns	ns	*	ns	
Number of values	6	6	6	6	6	6	
**Data does not exhibit a normal distribution, so use a non-parametric equivalent-the Kruskal-Wallis test.**							
Kruskal-Wallis test							
P value	0.0003						
Exact or approximate P value?	Approximate						
P value summary	***						
Do the medians vary signif. (P < 0.05)?	Yes						
Number of groups	6						
Kruskal-Wallis statistic	23.11						
Dunn's multiple comparisons test	Mean rank diff.	Significant?	Summary	Adjusted P Value			
CFA vs. Naive	-23.33	Yes	***	0.0006			
CFA vs. CFA + veh	1.333	No	ns	>0.9999			
CFA vs. CFA + GSK 5µg	-5.667	No	ns	>0.9999			
CFA vs. CFA + GSK 25µg	-10.83	No	ns	0.3742			
CFA vs. CFA + GSK 50µg	-13.5	No	ns	0.1321			
Figure 3B PWL CFA vs Baseline							
	BL	1/4	1	3	5	7	10
Test for normal distribution							
Shapiro-Wilk test							
W	0.8536	0.9325	0.9325	0.9206	0.8241	0.9567	0.8763
P value	0.1681	0.5997	0.5997	0.5094	0.0958	0.7942	0.2524

Passed normality test (alpha=0.05)?	Yes	Yes	Yes	Yes	Yes	Yes	Yes
P value summary	ns	ns	ns	ns	ns	ns	ns
Number of values	6	6	6	6	6	6	6
**Data exhibits a normal distribution**							
ANOVA summary							
F	119.8						
P value	<0.0001						
P value summary	****						
Significant diff. among means (P < 0.05)?	Yes						
R squared	0.9536						
Dunnett's multiple comparisons test	Mean Diff.	95.00% CI of diff.	Significant?	Summary	Adjusted P Value		
BL vs. 1/4	9.46	8.264 to 10.65	Yes	####	<0.0001		
BL vs. 1	8.96	7.764 to 10.15	Yes	####	<0.0001		
BL vs. 3	8.226	7.031 to 9.421	Yes	####	<0.0001		
BL vs. 5	7.76	6.564 to 8.955	Yes	####	<0.0001		
BL vs. 7	6.804	5.609 to 7.999	Yes	####	<0.0001		
BL vs. 10	3.504	2.309 to 4.699	Yes	####	<0.0001		
Two-way ANOVA	Ordinary						
Alpha	0.05						
Source of Variation	% of total variation	P value	P value summary	Significant?			
Interaction	10.39	<0.0001	****	Yes			
Row Factor	44.21	<0.0001	****	Yes			
Column Factor	40.59	<0.0001	****	Yes			

Figure 3C

Figure 3C PWT	BL	2	3	5	7
CFA + GSK-J4	25.368	4.338	6.736	11.978	11.978
	25.368	6.736	11.978	11.978	11.978
	25.368	4.338	6.736	6.736	6.736
	17.81	6.736	6.736	11.978	11.978
	25.368	4.338	6.736	11.978	11.978
	25.368	6.736	6.736	6.736	11.978
CFA + Vehicle	25.368	6.736	6.736	6.736	4.338
	25.368	4.338	4.338	2.805	4.338
	17.81	4.338	4.338	4.338	4.338
	25.368	2.805	4.338	2.805	6.736
	17.81	4.338	4.338	2.805	2.805
	25.368	4.338	4.338	4.338	6.736
**Figure 3C PWT BL**					
	CFA + GSK-J4	CFA + Veh			
Test for normal distribution					
Shapiro-Wilk test					
W	0.4961	0.6399			
P value	<0.0001	0.0014			
Passed normality test (alpha=0.05)?	No	No			
P value summary	****	**			
Number of values	6	6			
**Data does not exhibit a normal distribution, so use a non-parametric equivalent-the Mann Whitney test.**					
Mann Whitney test					
P value	>0.9999				
Exact or approximate P value?	Exact				
P value summary	ns				
Significantly different (P < 0.05)?	No				
One- or two-tailed P value?	Two-tailed				
Sum of ranks in column A,B	42 , 36				
Mann-Whitney U	15				
**Figure 3C PWT 2 d**					
	CFA + GSK-J4	CFA + Veh			

Test for normal distribution					
Shapiro-Wilk test					
W	0.6827	0.801			
P value	0.004	0.06			
Passed normality test (alpha=0.05)?	No	Yes			
P value summary	**	ns			
Number of values	6	6			
**Data does not exhibit a normal distribution, so use a non-parametric equivalent-the Mann Whitney test.**					
Mann Whitney test					
P value	0.3636				
Exact or approximate P value?	Exact				
P value summary	ns				
Significantly different (P < 0.05)?	No				
One- or two-tailed P value?	Two-tailed				
Sum of ranks in column A,B	46.50 , 31.50				
Mann-Whitney U	10.5				
**Figure 3C PWT 3 d**					
	CFA + GSK-J4	CFA + Veh			
Test for normal distribution					
Shapiro-Wilk test					
W	0.4961	0.4961			
P value	<0.0001	<0.0001			
Passed normality test (alpha=0.05)?	No	No			
P value summary	****	****			
Number of values	6	6			
**Data does not exhibit a normal distribution, so use a non-parametric equivalent-the Mann Whitney test.**					
Mann Whitney test					
P value	0.013				
Exact or approximate P value?	Exact				
P value summary	*				
Significantly different (P < 0.05)?	Yes				
One- or two-tailed P value?	Two-tailed				

Sum of ranks in column A,B	54.50 , 23.50				
Mann-Whitney U	2.5				
**Figure 3C PWT 5 d**					
	CFA + GSK-J4	CFA + Veh			
Test for normal distribution					
Shapiro-Wilk test					
W	0.6399	0.7977			
P value	0.0014	0.0561			
Passed normality test (alpha=0.05)?	No	Yes			
P value summary	**	ns			
Number of values	6	6			
**Data does not exhibit a normal distribution, so use a non-parametric equivalent-the Mann Whitney test.**					
Mann Whitney test					
P value	0.0065				
Exact or approximate P value?	Exact				
P value summary	**				
Significantly different (P < 0.05)?	Yes				
One- or two-tailed P value?	Two-tailed				
Sum of ranks in column A,B	56 , 22				
Mann-Whitney U	1				
**Figure 3C PWT 7 d**					
	CFA + GSK-J4	CFA + Veh			
Test for normal distribution					
Shapiro-Wilk test					
W	0.4961	0.8483			
P value	<0.0001	0.1525			
Passed normality test (alpha=0.05)?	No	Yes			
P value summary	****	ns			
Number of values	6	6			
**Data does not exhibit a normal distribution, so use a non-parametric equivalent-the Mann Whitney test.**					
Mann Whitney test					
P value	0.0065				

Exact or approximate P value?	Exact				
P value summary	**				
Significantly different (P < 0.05)?	Yes				
One- or two-tailed P value?	Two-tailed				
Sum of ranks in column A,B	56 , 22				
Mann-Whitney U	1				
**Figure 3C** PWL CFA Veh: vs Baseline					
	BL	2	3	5	7
Test for normal distribution					
Shapiro-Wilk test					
W	0.6399	0.801	0.4961	0.7977	0.8483
P value	0.0014	0.06	<0.0001	0.0561	0.1525
Passed normality test (alpha=0.05)?	No	Yes	No	Yes	Yes
P value summary	**	ns	****	ns	ns
**Data does not exhibit a normal distribution, so use a non-parametric equivalent-the Kruskal-Wallis test.**					
Kruskal-Wallis test					
P value	0.0019				
Exact or approximate P value?	Approximate				
P value summary	**				
Do the medians vary signif. (P < 0.05)?	Yes				
Number of groups	5				
Kruskal-Wallis statistic	17.02				
Dunn's multiple comparisons test	Mean rank diff.	Significant?	Summary	Adjusted P Value	
BL vs. 2	15	Yes	##	0.0069	
BL vs. 3	13.42	Yes	#	0.0203	
BL vs. 5	18.17	Yes	###	0.0006	
BL vs. 7	13.42	Yes	#	0.0203	
Two-way RM ANOVA	Matching: Stacked				
Assume sphericity?	No				
Alpha	0.05				
Source of Variation	% of total	P value	P value	Significant	

	variation		summary	?	
Interaction	2.385	0.0038	**	Yes	
Row Factor	84.4	<0.0001	****	Yes	
Column Factor	5.639	0.0006	***	Yes	

Figure 3D

Figure 3D PWL	BL	2	3	5	7
CFA + GSK-J4	11.533	4.933	6.933	8.367	10
	12.6	3.4	6.3	9.467	10
	13	4.567	6.433	8.833	9.533
	12.433	4.9	6.2	7.833	8.8
	12.3	4.567	5.967	7.6	8.633
	12.067	3.933	6.333	7.733	9.8
CFA + Vehicle	12.833	4.567	5.367	6.467	7.033
	12.5	4.833	5.133	5.6	5.7
	12.1	3.967	4.767	4.733	5.833
	11.533	4.8	5.967	5.833	6.967
	11.633	4.3	5.6	6	6.833
	13.133	4.5	5.233	6.167	6.667
Figure 3D **PWL BL**					
	CFA + GSK-J4	CFA + Veh			
Test for normal distribution					
Shapiro-Wilk test					
W	0.9861	0.9388			
P value	0.9776	0.6499			
Passed normality test (alpha=0.05)?	Yes	Yes			
P value summary	ns	ns			
Number of values	6	6			
**Data exhibits a normal distribution**					
Unpaired t test					
P value	0.9219				
P value summary	ns				
Significantly different (P < 0.05)?	No				
One- or two-tailed P value?	Two-tailed				
t, df	t=0.1006, df=10				
Figure 3D **PWL 2 d**					
	CFA +	CFA + Veh			

	GSK-J4				
Test for normal distribution					
Shapiro-Wilk test					
W	0.8749	0.9339			
P value	0.2465	0.6109			
Passed normality test (alpha=0.05)?	Yes	Yes			
P value summary	ns	ns			
Number of values	6	6			
**Data exhibits a normal distribution**					
Unpaired t test					
P value	0.6987				
P value summary	ns				
Significantly different (P < 0.05)?	No				
One- or two-tailed P value?	Two-tailed				
t, df	t=0.3984, df=10				
Figure 3D **PWL 3 d**					
	CFA + GSK-J4	CFA + Veh			
Test for normal distribution					
Shapiro-Wilk test					
W	0.9175	0.9909			
P value	0.4878	0.9912			
Passed normality test (alpha=0.05)?	Yes	Yes			
P value summary	ns	ns			
Number of values	6	6			
**Data exhibits a normal distribution**					
Unpaired t test					
P value	0.0008				
P value summary	***				
Significantly different (P < 0.05)?	Yes				
One- or two-tailed P value?	Two-tailed				
t, df	t=4.771, df=10				
Figure 3D **PWL 5 d**					
	CFA + GSK-J4	CFA + Veh			

Test for normal distribution					
Shapiro-Wilk test					
W	0.9038	0.9232			
P value	0.3972	0.529			
Passed normality test (alpha=0.05)?	Yes	Yes			
P value summary	ns	ns			
Number of values	6	6			
**Data exhibits a normal distribution**					
Unpaired t test					
P value	<0.0001				
P value summary	****				
Significantly different (P < 0.05)?	Yes				
One- or two-tailed P value?	Two-tailed				
t, df	t=6.484, df=10				
Figure 3D **PWL 7 d**					
	CFA + GSK-J4	CFA + Veh			
Test for normal distribution					
Shapiro-Wilk test					
W	0.8423	0.8207			
P value	0.1361	0.0895			
Passed normality test (alpha=0.05)?	Yes	Yes			
P value summary	ns	ns			
Number of values	6	6			
**Data exhibits a normal distribution**					
Unpaired t test					
P value	<0.0001				
P value summary	****				
Significantly different (P < 0.05)?	Yes				
One- or two-tailed P value?	Two-tailed				
t, df	t=8.592, df=10				
Figure 3D PWL CFA + Veh: vs Baseline					
	BL	2	3	5	7
Test for normal distribution					

Shapiro-Wilk test					
W	0.9388	0.9339	0.9909	0.9232	0.8207
P value	0.6499	0.6109	0.9912	0.529	0.0895
Passed normality test (alpha=0.05)?	Yes	Yes	Yes	Yes	Yes
P value summary	ns	ns	ns	ns	ns
**Data exhibits a normal distribution**					
ANOVA summary					
F	207.2				
P value	<0.0001				
P value summary	****				
Significant diff. among means (P < 0.05)?	Yes				
R squared	0.9707				
Dunnett's multiple comparisons test	Mean Diff.	95.00% CI of diff.	Significant?	Summary	Adjusted P Value
BL vs. 2	7.794	6.998 to 8.590	Yes	####	<0.0001
BL vs. 3	6.944	6.148 to 7.740	Yes	####	<0.0001
BL vs. 5	6.489	5.693 to 7.284	Yes	####	<0.0001
BL vs. 7	5.783	4.987 to 6.579	Yes	####	<0.0001
Two-way RM ANOVA	Matching: Stacked				
Assume sphericity?	No				
Alpha	0.05				
Source of Variation	% of total variation	P value	P value summary	Significant?	
Interaction	4.812	<0.0001	****	Yes	
Row Factor	87.11	<0.0001	****	Yes	
Column Factor	5.01	<0.0001	****	Yes	

Figure 3E

Western blot	Con	1/4	1	3	7	14	21
	102.78	255.34	173.38	155.59	117.42	126.37	104.85
	99.79	264.47	181.69	155.24	155.65	111.76	109.41
	98.69	243.15	151.28	173.07	127.82	130.64	108.85
	98.36	296.83	177.10	150.50	103.62	104.59	106.23

	100.38	288.66	178.79	150.83	137.61	108.28	104.88
Figure 3E	Con	1/4	1	3	7	14	21
Test for normal distribution							
Shapiro-Wilk test							
W	0.9086	0.9398	0.7766	0.7589	0.9963	0.8883	0.8453
P value	0.4593	0.6644	0.0515	0.0359	0.9965	0.3486	0.1803
Passed normality test (alpha=0.05)?	Yes	Yes	Yes	No	Yes	Yes	Yes
P value summary	ns	ns	ns	*	ns	ns	ns
Number of values	5	5	5	5	5	5	5
**Data does not exhibit a normal distribution, so use a non-parametric equivalent-the Kruskal-Wallis test.**							
Kruskal-Wallis test							
P value	<0.0001						
Exact or approximate P value?	Approximate						
P value summary	****						
Do the medians vary signif. (P < 0.05)?	Yes						
Number of groups	7						
Kruskal-Wallis statistic	30.53						
Dunn's multiple comparisons test	Mean rank diff.	Significant?	Summary	Adjusted P Value			
Con vs. 1/4	-30	Yes	****	<0.0001			
Con vs. 1	-24.2	Yes	**	0.0011			
Con vs. 3	-19.8	Yes	*	0.0135			
Con vs. 7	-13.4	No	ns	0.232			
Con vs. 14	-10.2	No	ns	0.6931			
Con vs. 21	-7.4	No	ns	>0.9999			

Figure 3F

Western blot	Con	1/4	1	3	7	14	21
	99.94	190.24	273.50	149.01	124.31	106.31	102.05
	94.86	202.23	259.77	170.69	150.11	130.26	109.69
	106.54	153.35	303.15	165.08	146.13	107.86	109.20
	99.19	159.28	256.17	179.29	102.17	134.66	106.50
	99.48	212.12	282.28	186.48	147.09	118.21	102.05

Figure 3F							
	Con	1/4	1	3	7	14	21
Test for normal distribution							
Shapiro-Wilk test							
W	0.8997	0.9012	0.9369	0.9754	0.8283	0.8955	0.8376
P value	0.4084	0.4167	0.6443	0.9084	0.1351	0.3853	0.1584
Passed normality test (alpha=0.05)?	Yes	Yes	Yes	Yes	Yes	Yes	Yes
P value summary	ns	ns	ns	ns	ns	ns	ns
Number of values	5	5	5	5	5	5	5
**Data exhibits a normal distribution**							
Dunnett's multiple comparisons test	Mean Diff.	95.00% CI of diff.	Significant?	Summary	Adjusted P Value		
Con vs. 1/4	-83.44	-111.6 to -55.29	Yes	****	<0.0001		
Con vs. 1	-175	-203.1 to -146.8	Yes	****	<0.0001		
Con vs. 3	-70.11	-98.26 to -41.96	Yes	****	<0.0001		
Con vs. 7	-33.96	-62.11 to -5.809	Yes	*	0.0134		
Con vs. 14	-19.46	-47.61 to 8.692	No	ns	0.2657		
Con vs. 21	-5.897	-34.05 to 22.25	No	ns	0.9821		

Figure 3G

Western blot	Con	CFA	CFA + GSK	CFA + Veh	Con
	102.95	304.09	99.33	328.90	102.95
	103.71	383.39	109.71	290.12	103.71
	98.07	260.48	106.53	320.08	98.07
	105.32	351.21	91.36	379.03	105.32
	89.95	279.09	85.92	354.16	89.95
Figure 3G					
	Con	CFA	CFA +	CFA +	

			GSK	Veh	
Test for normal distribution					
Shapiro-Wilk test					
W	0.8618	0.9472	0.9478	0.9899	
P value	0.2348	0.7175	0.7213	0.9792	
Passed normality test (alpha=0.05)?	Yes	Yes	Yes	Yes	
P value summary	ns	ns	ns	ns	
Number of values	5	5	5	5	
**Data exhibits a normal distribution**					
ANOVA summary					
F	87.99				
P value	<0.0001				
P value summary	****				
Significant diff. among means (P < 0.05)?	Yes				
R squared	0.9429				
Dunnett's multiple comparisons test	Mean Diff.	95.00% CI of diff.	Significant ?	Summary	Adjusted P Value
Con vs. CFA	-215.7	-266.7 to -164.6	Yes	****	<0.0001
Con vs. CFA + GSK	1.43	-49.61 to 52.47	No	ns	0.9997
Con vs. CFA + Veh	-234.5	-285.5 to -183.4	Yes	****	<0.0001
ANOVA summary					
F	87.99				
P value	<0.0001				
P value summary	****				
Significant diff. among means (P < 0.05)?	Yes				
R squared	0.9429				
Dunnett's multiple comparisons test	Mean Diff.	95.00% CI of diff.	Significant ?	Summary	Adjusted P Value
CFA vs. Con	215.7	164.6 to 266.7	Yes	####	<0.0001
CFA vs. CFA + GSK	217.1	166.0 to 268.1	Yes	####	<0.0001
CFA vs. CFA + Veh	-18.81	-69.85 to 32.23	No	ns	0.6643

Figure 3H

Western blot	Con	CFA	CFA + GSK	CFA + Veh	Western blot
	100.50	285.96	95.03	305.38	
	100.94	281.41	113.98	312.00	
	104.85	279.87	85.77	284.59	
	97.16	260.42	96.67	303.80	
	96.56	315.84	98.70	324.02	
Figure 3H					
	Con	CFA	CFA + GSK	CFA + Veh	
Test for normal distribution					
Shapiro-Wilk test					
W	0.9258	0.9199	0.9267	0.9638	
P value	0.568	0.5295	0.5743	0.8342	
Passed normality test (alpha=0.05)?	Yes	Yes	Yes	Yes	
P value summary	ns	ns	ns	ns	
Number of values	5	5	5	5	
**Data exhibits a normal distribution**					
ANOVA summary					
F	359				
P value	<0.0001				
P value summary	****				
Significant diff. among means (P < 0.05)?	Yes				
R squared	0.9854				
Dunnett's multiple comparisons test	Mean Diff.	95.00% CI of diff.	Significant ?	Summary	Adjusted P Value
Con vs. CFA	-184.7	-206.7 to -162.7	Yes	****	<0.0001
Con vs. CFA + GSK	1.969	-20.03 to 23.97	No	ns	0.991
Con vs. CFA + Veh	-206	-228.0 to -184.0	Yes	****	<0.0001
ANOVA summary					
F	359				
P value	<0.0001				
P value summary	****				
Significant diff. among means (P < 0.05)?	Yes				

R squared	0.9854				
Dunnett's multiple comparisons test	Mean Diff.	95.00% CI of diff.	Significant?	Summary	Adjusted P Value
CFA vs. Con	184.7	162.7 to 206.7	Yes	####	<0.0001
CFA vs. CFA + GSK	186.7	164.7 to 208.7	Yes	####	<0.0001
CFA vs. CFA + Veh	-21.26	-43.26 to 0.7364	No	ns	0.0591

Figure 3I-J

Figure 1C KDM6B						
Con	6 h	1 d	3 d	7 d	14 d	21 d
100.6437991	260.0761272	172.7400516	131.8685218	121.8685218	87.6189089	97.6189089
102.7738007	192.6279098	151.5179501	107.0621818	97.0621818	107.2634348	97.2634348
101.6377998	257.5661525	220.9018009	155.5465621	125.5465621	114.2182224	104.2182224
96.2398372	200.5034942	182.1147788	101.4873741	101.4873741	96.94316081	96.94316081
98.70476317	216.2581114	229.9899875	140.1467269	140.1467269	106.3622373	110.3622373
Mean of each column						
100.000	225.406	191.453	127.222	117.222	102.481	101.281
Figure 1E H3K27me3						
Con	6 h	1 d	3 d	7 d	14 d	21 d
97.4551436	59.76	59.66	68.41	80.04	97.39	96.63
112.8452856	40.83	53.81	60.85	60.27	87.27	116.02
93.8439432	44.25	69.1	64.86	69.06	89.88	82.41
102.352007	37.09	47.52	57.28	69.15	85.63	106.49
93.50362065	45.69	54.15	74.09	69.86	75.85	96.16
Mean of each column						
100	45.524	56.848	65.098	69.676	87.204	99.542
Figure 3E TNF-α						
Con	6 h	1 d	3 d	7 d	14 d	21 d

102.7755848	255.344	173.381	155.587	117.417	126.373	104.854
99.79481884	264.474	181.69	155.243	155.646	111.761	109.412
98.68976885	243.15	151.283	173.074	127.817	130.642	108.845
98.36033139	296.832	177.098	150.498	103.623	104.593	106.232
100.3794961	288.66	178.79	150.829	137.612	108.278	104.875
Mean of each column						
100.000	269.692	172.448	157.046	128.423	116.329	106.844
Fig 1C KDM6B	Fig 5E TNF-α					
100	100					
225.406	269.692					
191.453	172.448					
127.222	157.046					
117.222	128.423					
102.481	116.329					
101.281	106.844					
Pearson r						
r	0.9423					
95% confidence interval	0.6514 to 0.9917					
R squared	0.8878					
P value						
P (two-tailed)	0.0015					
P value summary	**					
Significant? (alpha = 0.05)	Yes					
Figure 1E H3K27me3	Figure 3E TNF-α					
100	100					
45.524	269.692					

56.848	172.448					
65.098	157.046					
69.676	128.423					
87.204	116.329					
99.542	106.844					
Pearson r						
r	-0.8811					
95% confidence interval	-0.9823 to -0.3805					
R squared	0.7763					
P value						
P (two-tailed)	0.0088					
P value summary	**					
Significant? (alpha = 0.05)	Yes					

Figure 3K and L

Figure 1K KDM6B						
Con	6 h	1 d	3 d	7 d	14 d	21 d
102.7755848	157.141	266.558	146.6680225	134.2999795	126.6680225	101.7023145
99.79481883	165.167	279.072	172.2374065	113.2397729	117.2374065	95.7282284
98.68976884	147.647	284.982	137.8189408	121.2868098	113.8189408	94.7282693
98.36033138	128.053	275.843	161.3510942	142.6054718	102.3510942	113.7572825
100.3794961	140.086	264.49	157.0921802	123.3453622	121.0921802	93.5895993
Mean of each column						
100.000	147.619	274.189	155.034	126.955	116.234	99.901
Figure 1O H3K27me3						
Con	6 h	1 d	3 d	7 d	14 d	21 d
101.2794639	47.42	47.83	55.38	76.67	76.64	86.59
105.952544	41.55	46.5	49.43	81.44	82.44	118.82

2						
99.2221329 6	34.59	49.37	58.79	67.5	91.25	88
94.1963673 4	41.64	53.89	60.47	48.84	65.36	104.08
99.3494915 5	48.81	56.16	59.28	71.08	81.28	98.2
Mean of each column						
100.000	42.802	50.750	56.670	69.106	79.394	99.138
Figurer 3F TNF-α						
Con	6 h	1 d	3 d	7 d	14 d	21 d
99.9352083 4	190.238	273.5	149.007	124.306	106.305	102.049
94.8585448	202.229	259.766	170.688	150.108	130.261	109.687
106.537834	153.349	303.151	165.084	146.131	107.86	109.203
99.1904993 3	159.284	256.17	179.286	102.166	134.664	106.495
99.4779135 5	212.122	282.277	186.484	147.092	118.209	102.051
Mean of each column						
100.000	183.444	254.973	170.110	133.961	119.460	105.897
Figure 1K KDM6B	Figure 3F TNF-α					
100	100					
147.619	183.444					
274.189	254.973					
155.034	170.11					
126.955	133.961					
116.234	119.46					
99.901	105.897					
Pearson r						
r	0.9635					
95% confidence interval	0.7666 to 0.9948					
R squared	0.9282					
P value						
P	0.0005					

(two-tailed)						
P value summary	***					
Significant? (alpha = 0.05)	Yes					
Figure 1O H3K27me3	Figure 3F TNF-α					
100	100					
42.802	183.444					
50.75	254.973					
56.67	170.11					
69.106	133.961					
79.394	119.46					
99.138	105.897					
Pearson r						
r	-0.8348					
95% confidence interval	-0.9750 to -0.2201					
R squared	0.6969					
P value						
P (two-tailed)	0.0194					
P value summary	*					
Significant? (alpha = 0.05)	Yes					

Figure 4A

Figure 4A PWT	BL	1/4	1	3	5	7	10
Con	25.368	21.1	23.2	21.8	24.8	21.1	24.4
	25.368	21.7	21.7	23.2	24.3	24.9	22.9
	23.368	23.7	23	23.7	22.1	21.8	21.3
	21.368	22.1	22.2	21.4	23	24.3	22.5
	20.48	23.5	24.5	22	22.7	20.5	24.4
CFA	25.368	3.933	2.933	5.313	8.058	9.058	11.933
	20.48	3.133	5.313	3.533	7.313	8.358	9.313
	24.368	3.533	2.933	4.933	6.313	8.158	7.833

	25.368	2.033	2.933	2.333	5.313	7.058	7.933
	25.368	1.933	5.313	3.933	5.313	10.058	9.313
CFA + AAV-KDM6B shRNA	25.368	8.98	11.868	10.813	12.98	12.48	15.313
	21.368	10.98	10.98	11.98	13.98	13.48	13.48
	23.368	10.558	8.98	13.558	10.98	15.48	14.058
	20.48	10.98	10.98	13.868	13.868	14.368	16.368
	24.368	12.868	12.558	11.868	11.98	16.368	16.368
CFA + AAV-shRNA NC	25.368	2.033	8.058	8.058	8.058	10.48	12.058
	23.368	3.933	5.313	5.313	5.313	10.48	10.313
	25.368	1.933	2.933	5.313	8.058	9.058	8.933
	20.48	4	2.933	2.933	5.313	8.058	7.933
	20.368	2.933	2.933	2.933	5.313	8.058	8.933
Figure 4A BL PWT							
	Con	CFA + veh	CFA + AAV-KDM6B shRNA	CFA + AAV-shRNA NC			
Test for normal distribution							
Shapiro-Wilk test							
W	0.8799	0.6783	0.9492	0.8233			
P value	0.309	0.0056	0.7317	0.1239			
Passed normality test (alpha=0.05)?	Yes	No	Yes	Yes			
P value summary	ns	**	ns	ns			
Number of values	5	5	5	5			
**Data exhibits a normal distribution**							
ANOVA summary							
F	0.3325						
P value	0.802						
P value summary	ns						
Significant diff. among means (P < 0.05)?	No						
R squared	0.05869						
Dunnett's multiple comparisons test	Mean Diff.	95.00% CI of diff.	Significant?	Summary	Adjusted P Value		
CFA + veh vs. Con	1	-2.653 to 4.653	No	ns	0.8199		
CFA + veh vs. CFA + AAV-KDM6B shRNA	1.2	-2.453 to 4.853	No	ns	0.7323		
CFA + veh vs. CFA +	1.2	-2.453	No	ns	0.7323		

AAV-shRNA NC		to 4.853					
Figure 4A 6h PWT							
	Con	CFA + veh	CFA + AAV-KDM6B shRNA	CFA + AAV-shRNA NC			
Test for normal distribution							
Shapiro-Wilk test							
W	0.9049	0.8917	0.9387	0.8516			
P value	0.4375	0.3657	0.6567	0.1995			
Passed normality test (alpha=0.05)?	Yes	Yes	Yes	Yes			
P value summary	ns	ns	ns	ns			
Number of values	5	5	5	5			
**Data exhibits a normal distribution**							
ANOVA summary							
F	339.3						
P value	<0.0001						
P value summary	****						
Significant diff. among means (P < 0.05)?	Yes						
R squared	0.9845						
Dunnett's multiple comparisons test	Mean Diff.	95.00% CI of diff.	Significant?	Summary	Adjusted P Value		
CFA + veh vs. Con	-19.51	-21.34 to -17.67	Yes	****	<0.0001		
CFA + veh vs. CFA + AAV-KDM6B shRNA	-7.96	-9.794 to -6.127	Yes	****	<0.0001		
CFA + veh vs. CFA + AAV-shRNA NC	-0.0534	-1.887 to 1.780	No	ns	0.9996		
Figure 4A 1d PWT							
	Con	CFA + veh	CFA + AAV-KDM6B shRNA	CFA + AAV-shRNA NC			
Test for normal distribution							
Kolmogorov-Smirnov test							

KS distance	0.1969	0.3674	0.2724	0.3455			
P value	>0.1000	0.0261	>0.1000	0.051			
Passed normality test (alpha=0.05)?	Yes	No	Yes	Yes			
P value summary	ns	*	ns	ns			
Number of values	5	5	5	5			
**Data does not exhibit a normal distribution, so use a non-parametric equivalent-the Kruskal-Wallis test.**							
Kruskal-Wallis test							
P value	0.0009						
Exact or approximate P value?	Approximate						
P value summary	***						
Do the medians vary signif. (P < 0.05)?	Yes						
Number of groups	4						
Kruskal-Wallis statistic	16.58						
Dunn's multiple comparisons test	Mean rank diff.	Significant?	Summary	Adjusted P Value			
CFA + veh vs. Con	-12.7	Yes	**	0.0017			
CFA + veh vs. CFA + AAV-KDM6B shRNA	-7.7	No	ns	0.11			
CFA + veh vs. CFA + AAV-shRNA NC	-0.4	No	ns	>0.9999			
Figure 4A 3d PWT							
	Con	CFA + veh	CFA + AAV-KDM6B shRNA	CFA + AAV-shRNA NC			
Test for normal distribution							
Shapiro-Wilk test							
W	0.9054	0.9607	0.9186	0.8778			
P value	0.4406	0.8128	0.521	0.2995			
Passed normality test (alpha=0.05)?	Yes	Yes	Yes	Yes			
P value summary	ns	ns	ns	ns			
Number of values	5	5	5	5			
**Data exhibits a normal distribution**							
ANOVA summary							
F	171.4						

P value	<0.0001						
P value summary	****						
Significant diff. among means (P < 0.05)?	Yes						
R squared	0.9698						
Dunnett's multiple comparisons test	Mean Diff.	95.00% CI of diff.	Significant?	Summary	Adjusted P Value		
CFA + veh vs. Con	-18.41	-20.80 to -16.02	Yes	****	<0.0001		
CFA + veh vs. CFA + AAV-KDM6B shRNA	-8.408	-10.80 to -6.019	Yes	****	<0.0001		
CFA + veh vs. CFA + AAV-shRNA NC	-0.901	-3.290 to 1.488	No	ns	0.6494		
Figure 4A 5d PWT							
	Con	CFA + veh	CFA + AAV-KDM6B shRNA	CFA + AAV-shRNA NC			
Test for normal distribution							
Shapiro-Wilk test							
W	0.931	0.897	0.9173	0.684			
P value	0.6031	0.3937	0.513	0.0065			
Passed normality test (alpha=0.05)?	Yes	Yes	Yes	No			
P value summary	ns	ns	ns	**			
Number of values	5	5	5	5			
**Data does not exhibit a normal distribution, so use a non-parametric equivalent-the Kruskal-Wallis test.**							
Kruskal-Wallis test							
P value	0.001						
Exact or approximate P value?	Approximate						
P value summary	***						
Do the medians vary signif. (P < 0.05)?	Yes						
Number of groups	4						
Kruskal-Wallis statistic	16.37						

Dunn's multiple comparisons test	Mean rank diff.	Significant?	Summary	Adjusted P Value			
CFA + veh vs. Con	-12.4	Yes	**	0.0025			
CFA + veh vs. CFA + AAV-KDM6B shRNA	-7.4	No	ns	0.1379			
CFA + veh vs. CFA + AAV-shRNA NC	0.2	No	ns	>0.9999			
Figure 4A 7d PWT							
	Con	CFA + veh	CFA + AAV-KDM6B shRNA	CFA + AAV-shRNA NC			
Test for normal distribution							
Shapiro-Wilk test							
W	0.8846	0.9881	0.9831	0.8159			
P value	0.3309	0.9726	0.9506	0.1085			
Passed normality test (alpha=0.05)?	Yes	Yes	Yes	Yes			
P value summary	ns	ns	ns	ns			
Number of values	5	5	5	5			
**Data exhibits a normal distribution**							
ANOVA summary							
F	92.93						
P value	<0.0001						
P value summary	****						
Significant diff. among means (P < 0.05)?	Yes						
R squared	0.9457						
Dunnett's multiple comparisons test	Mean Diff.	95.00% CI of diff.	Significant?	Summary	Adjusted P Value		
CFA + veh vs. Con	-13.98	-16.44 to -11.53	Yes	****	<0.0001		
CFA + veh vs. CFA + AAV-KDM6B shRNA	-5.897	-8.352 to -3.442	Yes	****	<0.0001		
CFA + veh vs. CFA + AAV-shRNA NC	-0.6888	-3.144 to 1.766	No	ns	0.8095		

Figure 4A 10d PWT							
	Con	CFA + veh	CFA + AAV-KDM6B shRNA	CFA + AAV-shRNA NC			
Test for normal distribution							
Shapiro-Wilk test							
W	0.9039	0.8542	0.8803	0.9257			
P value	0.4317	0.2083	0.3108	0.5671			
Passed normality test (alpha=0.05)?	Yes	Yes	Yes	Yes			
P value summary	ns	ns	ns	ns			
Number of values	5	5	5	5			
**Data exhibits a normal distribution**							
Dunnett's multiple comparisons test	Mean Diff.	95.00% CI of diff.	Significant?	Summary	Adjusted P Value		
CFA + veh vs. Con	-13.84	-16.27 to -11.40	Yes	****	<0.0001		
CFA + veh vs. CFA + AAV-KDM6B shRNA	-5.852	-8.283 to -3.422	Yes	****	<0.0001		
CFA + veh vs. CFA + AAV-shRNA NC	-0.369	-2.799 to 2.061	No	ns	0.9599		
Figure 4A PWT CFA: vs Baseline							
	BL	1/4	1	3	5	7	10
Test for normal distribution							
Shapiro-Wilk test							
W	0.6783	0.8917	0.684	0.9607	0.897	0.9881	0.8542
P value	0.0056	0.3657	0.0065	0.8128	0.3937	0.9726	0.2083
Passed normality test (alpha=0.05)?	No	Yes	No	Yes	Yes	Yes	Yes
P value summary	**	ns	**	ns	ns	ns	ns
Number of values	5	5	5	5	5	5	5
**Data exhibits a normal distribution**							
ANOVA summary							
F	136.3						
P value	<0.0001						
P value summary	****						
Significant diff. among means	Yes						

(P < 0.05)?							
R squared	0.9669						
Dunnett's multiple comparisons test	Mean Diff.	95.00% CI of diff.	Significant?	Summary	Adjusted P Value		
BL vs. 1/4	21.28	18.85 to 23.71	Yes	####	<0.0001		
BL vs. 1	20.31	17.88 to 22.74	Yes	####	<0.0001		
BL vs. 3	20.18	17.75 to 22.61	Yes	####	<0.0001		
BL vs. 5	17.73	15.30 to 20.16	Yes	####	<0.0001		
BL vs. 7	15.65	13.22 to 18.08	Yes	####	<0.0001		
BL vs. 10	14.93	12.50 to 17.36	Yes	####	<0.0001		
Two-way ANOVA	Ordinary						
Alpha	0.05						
Source of Variation	% of total variation	P value	P value summary	Significant?			
Interaction	11.27	<0.0001	****	Yes			
Row Factor	29.79	<0.0001	****	Yes			
Column Factor	55.79	<0.0001	****	Yes			

Figure 4B

Figure 4B PWL	BL	1/4	1	3	5	7	10
Con	12.8	12.8	11.875	11.781	12.089	12.198	12.757
	12.1	12.4	12.44	12.207	11.759	12.504	12.773
	12.2	11.4	12.609	11.768	11.8	12.589	11.39
	12.2	11.1	11.929	12.626	12.011	12.617	11.673
	12.9	12.9	13.579	12.433	12.69	11.737	13.383
CFA + veh	11.1	3	2.3	2.1	3.6	3.9	7.91
	12.1	3.3	3.8	3.6	2.8	3.8	6.8
	13.8	2.1	4.1	3.3	3	4.7	7
	13.3	1.7	1.5	3	2.8	3.5	6.3
	12.4	1.9	2.7	3.1	3.4	3.5	7

CFA + AAV-KDM6B shRNA	11.5	6.1	6.7	7.3	8.2	6	8.9
	11.4	4.7	6.4	7.6	6.8	7.6	9.5
	12.9	7.5	6.1	5	7.1	7.9	8.8
	13.1	5.1	6.2	5.6	6.8	7.6	9
	12.5	5.3	7	6.4	8	8.8	7.8
CFA + AAV-shRNA NC	11.3	2.5	3.3	2.9	3.5	3.1	5.6
	12.6	2.3	3.6	2.9	2.7	3.8	6.1
	11.4	2.1	2.5	3.3	4.8	4.5	6.5
	13.5	2.7	3.5	2.8	3	3.6	6.7
	12.2	2.6	2.7	3.1	2.8	4.7	7.4
Figure 4B BL PWL							
	Con	CFA + veh	CFA + AAV-KDM6B shRNA	CFA + AAV-shRNA NC			
Test for normal distribution							
Shapiro-Wilk test							
W	0.8002	0.9757	0.8659	0.93			
P value	0.0813	0.9103	0.2503	0.5962			
Passed normality test (alpha=0.05)?	Yes	Yes	Yes	Yes			
P value summary	ns	ns	ns	ns			
Number of values	5	5	5	5			
**Data exhibits a normal distribution**							
ANOVA summary							
F	0.1744						
P value	0.9122						
P value summary	ns						
Significant diff. among means (P < 0.05)?	No						
R squared	0.03166						
Dunnett's multiple comparisons test	Mean Diff.	95.00% CI of diff.	Significant?	Summary	Adjusted P Value		
CFA + veh vs. Con	0.1	-1.248 to 1.448	No	ns	0.9948		
CFA + veh vs. CFA + AAV-KDM6B shRNA	0.26	-1.088 to 1.608	No	ns	0.9238		
CFA + veh vs. CFA + AAV-shRNA NC	0.34	-1.008 to 1.688	No	ns	0.8511		

Figure 4B 6h PWL							
	Con	CFA + veh	CFA + AAV-KDM6B shRNA	CFA + AAV-shRNA NC			
Test for normal distribution							
Shapiro-Wilk test							
W	0.8693	0.883	0.8998	0.957			
P value	0.2634	0.3232	0.4088	0.7869			
Passed normality test (alpha=0.05)?	Yes	Yes	Yes	Yes			
P value summary	ns	ns	ns	ns			
Number of values	5	5	5	5			
**Data exhibits a normal distribution**							
ANOVA summary							
F	169.8						
P value	<0.0001						
P value summary	****						
Significant diff. among means (P < 0.05)?	Yes						
R squared	0.9695						
Dunnett's multiple comparisons test	Mean Diff.	95.00% CI of diff.	Significant?	Summary	Adjusted P Value		
CFA + veh vs. Con	-9.72	-11.01 to -8.433	Yes	****	<0.0001		
CFA + veh vs. CFA + AAV-KDM6B shRNA	-3.34	-4.627 to -2.053	Yes	****	<0.0001		
CFA + veh vs. CFA + AAV-shRNA NC	-0.04	-1.327 to 1.247	No	ns	0.9996		
Figure 4B 1d PWL							
	Con	CFA + veh	CFA + AAV-KDM6B shRNA	CFA + AAV-shRNA NC			
Test for normal distribution							
Shapiro-Wilk test							
W	0.887	0.948	0.943	0.8824			
P value	0.3424	0.7231	0.6871	0.3202			

Passed normality test (alpha=0.05)?	Yes	Yes	Yes	Yes			
P value summary	ns	ns	ns	ns			
Number of values	5	5	5	5			
**Data exhibits a normal distribution**							
ANOVA summary							
F	199.8						
P value	<0.0001						
P value summary	****						
Significant diff. among means (P < 0.05)?	Yes						
R squared	0.974						
Dunnett's multiple comparisons test	Mean Diff.	95.00% CI of diff.	Significant?	Summary	Adjusted P Value		
CFA + veh vs. Con	-9.606	-10.77 to -8.446	Yes	****	<0.0001		
CFA + veh vs. CFA + AAV-KDM6B shRNA	-3.6	-4.761 to -2.439	Yes	****	<0.0001		
CFA + veh vs. CFA + AAV-shRNA NC	-0.24	-1.401 to 0.9208	No	ns	0.9089		
Figure 4B 3d PWL							
	Con	CFA + veh	CFA + AAV-KDM6B shRNA	CFA + AAV-shRNA NC			
Test for normal distribution							
Shapiro-Wilk test							
W	0.8956	0.9018	0.9432	0.9053			
P value	0.3861	0.4202	0.6887	0.4399			
Passed normality test (alpha=0.05)?	Yes	Yes	Yes	Yes			
P value summary	ns	ns	ns	ns			
Number of values	5	5	5	5			
**Data exhibits a normal distribution**							
ANOVA summary							
F	217.2						
P value	<0.0001						

P value summary	****						
Significant diff. among means (P < 0.05)?	Yes						
R squared	0.976						
Dunnett's multiple comparisons test	Mean Diff.	95.00% CI of diff.	Significant?	Summary	Adjusted P Value		
CFA + veh vs. Con	-9.143	-10.22 to -8.069	Yes	****	<0.0001		
CFA + veh vs. CFA + AAV-KDM6B shRNA	-3.36	-4.434 to -2.286	Yes	****	<0.0001		
CFA + veh vs. CFA + AAV-shRNA NC	0.02	-1.054 to 1.094	No	ns	>0.9999		
Figure 4B 5d PWL							
	Con	CFA + veh	CFA + AAV-KDM6B shRNA	CFA + AAV-shRNA NC			
Test for normal distribution							
Shapiro-Wilk test							
W	0.8498	0.8668	0.8234	0.8238			
P value	0.1939	0.2538	0.124	0.1248			
Passed normality test (alpha=0.05)?	Yes	Yes	Yes	Yes			
P value summary	ns	ns	ns	ns			
Number of values	5	5	5	5			
**Data exhibits a normal distribution**							
ANOVA summary							
F	241.3						
P value	<0.0001						
P value summary	****						
Significant diff. among means (P < 0.05)?	Yes						
R squared	0.9784						
Dunnett's multiple comparisons test	Mean Diff.	95.00% CI of diff.	Significant?	Summary	Adjusted P Value		
CFA + veh vs. Con	-8.95	-9.943	Yes	****	<0.0001		

		to -7.957					
CFA + veh vs. CFA + AAV-KDM6B shRNA	-4.26	-5.253 to -3.267	Yes	****	<0.0001		
CFA + veh vs. CFA + AAV-shRNA NC	-0.24	-1.233 to 0.7528	No	ns	0.8656		
Figure 4B 7d PWL							
	Con	CFA + veh	CFA + AAV-KDM6B shRNA	CFA + AAV-shRNA NC			
Test for normal distribution							
Shapiro-Wilk test							
W	0.8407	0.8259	0.9144	0.9469			
P value	0.1669	0.1295	0.4944	0.7151			
Passed normality test (alpha=0.05)?	Yes	Yes	Yes	Yes			
P value summary	ns	ns	ns	ns			
Number of values	5	5	5	5			
**Data exhibits a normal distribution**							
ANOVA summary							
F	173.8						
P value	<0.0001						
P value summary	****						
Significant diff. among means (P < 0.05)?	Yes						
R squared	0.9702						
Dunnett's multiple comparisons test	Mean Diff.	95.00% CI of diff.	Significant?	Summary	Adjusted P Value		
CFA + veh vs. Con	-8.449	-9.559 to -7.339	Yes	****	<0.0001		
CFA + veh vs. CFA + AAV-KDM6B shRNA	-3.7	-4.810 to -2.590	Yes	****	<0.0001		
CFA + veh vs. CFA + AAV-shRNA NC	-0.06	-1.170 to 1.050	No	ns	0.998		

Figure 4B 10d PWL							
	Con	CFA + veh	CFA + AAV-KDM6B shRNA	CFA + AAV-shRNA NC			
Test for normal distribution							
Shapiro-Wilk test							
W	0.9091	0.9209	0.9013	0.9927			
P value	0.4621	0.5359	0.4173	0.9882			
Passed normality test (alpha=0.05)?	Yes	Yes	Yes	Yes			
P value summary	ns	ns	ns	ns			
Number of values	5	5	5	5			
**Data exhibits a normal distribution**							
ANOVA summary							
F	76.75						
P value	<0.0001						
P value summary	****						
Significant diff. among means (P < 0.05)?	Yes						
R squared	0.935						
Dunnett's multiple comparisons test	Mean Diff.	95.00% CI of diff.	Significant?	Summary	Adjusted P Value		
CFA + veh vs. Con	-5.393	-6.515 to -4.271	Yes	****	<0.0001		
CFA + veh vs. CFA + AAV-KDM6B shRNA	-1.798	-2.920 to -0.6760	Yes	**	0.002		
CFA + veh vs. CFA + AAV-shRNA NC	0.542	-0.5800 to 1.664	No	ns	0.4727		
Figure 4B PWL CFA: vs Baseline							
	BL	1/4	1	3	5	7	10
Test for normal distribution							
Shapiro-Wilk test							
W	0.9757	0.883	0.948	0.9018	0.8668	0.8259	0.9209
P value	0.9103	0.3232	0.7231	0.4202	0.2538	0.1295	0.5359
Passed normality test (alpha=0.05)?	Yes	Yes	Yes	Yes	Yes	Yes	Yes

P value summary	ns	ns	ns	ns	ns	ns	ns
Number of values	5	5	5	5	5	5	5
**Data exhibits a normal distribution**							
ANOVA summary							
F	124.2						
P value	<0.0001						
P value summary	****						
Significant diff. among means (P < 0.05)?	Yes						
R squared	0.9638						
Dunnett's multiple comparisons test	Mean Diff.	95.00% CI of diff.	Significant?	Summary	Adjusted P Value		
BL vs. 1/4	10.14	8.868 to 11.41	Yes	####	<0.0001		
BL vs. 1	9.66	8.388 to 10.93	Yes	####	<0.0001		
BL vs. 3	9.52	8.248 to 10.79	Yes	####	<0.0001		
BL vs. 5	9.42	8.148 to 10.69	Yes	####	<0.0001		
BL vs. 7	8.66	7.388 to 9.932	Yes	####	<0.0001		
BL vs. 10	5.538	4.266 to 6.810	Yes	####	<0.0001		
Two-way ANOVA	Ordinary						
Alpha	0.05						
Source of Variation	% of total variation	P value	P value summary	Significant?			
Interaction	11.13	<0.0001	****	Yes			
Row Factor	29.78	<0.0001	****	Yes			
Column Factor	56.56	<0.0001	****	Yes			

Figure 4C

Western blot	Con	CFA + veh	CFA + AAV-KDM6B shRNA	CFA + AAV-shRNA NC	

	79.20	275.28	134.41	361.35	
	110.76	374.38	115.38	313.92	
	120.85	279.43	121.77	357.16	
	94.59	374.14	96.14	283.17	
	94.59	364.14	196.14	283.17	
Figure 4C KDM6B	Con	CFA + veh	CFA + AAV-KDM6B shRNA	CFA + AAV-shRNA NC	
Test for normal distribution					
Shapiro-Wilk test					
W	0.9577	0.7444	0.873	0.8347	
P value	0.7918	0.0264	0.2788	0.1509	
Passed normality test (alpha=0.05)?	Yes	No	Yes	Yes	
P value summary	ns	*	ns	ns	
Number of values	5	5	5	5	
**Data does not exhibit a normal distribution, so use a non-parametric equivalent-the Kruskal-Wallis test.**					
Kruskal-Wallis test					
P value	0.0015				
Exact or approximate P value?	Approximate				
P value summary	**				
Do the medians vary signif. (P < 0.05)?	Yes				
Number of groups	4				
Kruskal-Wallis statistic	15.41				
Dunn's multiple comparisons test	Mean rank diff.	Significant?	Summary	Adjusted P Value	
Con vs. CFA + veh	-12.4	Yes	**	0.0027	
Con vs. CFA + AAV-KDM6B shRNA	-3.8	No	ns	0.9284	
Con vs. CFA + AAV-shRNA NC	-11.4	Yes	**	0.0069	
Kruskal-Wallis test					
P value	0.002				
Exact or approximate P value?	Exact				
P value summary	**				
Do the medians vary signif. (P < 0.05)?	Yes				
Number of groups	3				
Kruskal-Wallis statistic	9.517				
Dunn's multiple comparisons test	Mean rank	Significant?	Summary	Adjusted P	

	diff.			Value	
CFA + veh vs. CFA + AAV-KDM6B shRNA	8	Yes	##	0.0093	
CFA + veh vs. CFA + AAV-shRNA NC	1	No	ns	>0.9999	
Figure 4C TNF-α					
Western blot	Con	CFA + veh	CFA + AAV-KDM6B shRNA	CFA + AAV-shRNA NC	
	105.98	324.12	134.75	396.46	
	96.74	296.78	142.13	299.37	
	91.63	390.26	100.15	291.16	
	100.78	282.32	121.13	384.99	
	104.87	358.33	153.37	277.46	
Figure 4C TNF-α	Con	CFA + veh	CFA + AAV-KDM6B shRNA	CFA + AAV-shRNA NC	
Test for normal distribution					
Shapiro-Wilk test					
W	0.9393	0.9559	0.9705	0.8184	
P value	0.6611	0.7794	0.8784	0.1134	
Passed normality test (alpha=0.05)?	Yes	Yes	Yes	Yes	
P value summary	ns	ns	ns	ns	
Number of values	5	5	5	5	
**Data exhibits a normal distribution**					
ANOVA summary					
F	55.77				
P value	<0.0001				
P value summary	****				
Significant diff. among means (P < 0.05)?	Yes				
R squared	0.9127				
Dunnett's multiple comparisons test	Mean Diff.	95.00% CI of diff.	Significant?	Summary	Adjusted P Value
Con vs. CFA + veh	-230.4	-291.6 to -169.1	Yes	****	<0.0001
Con vs. CFA + AAV-KDM6B shRNA	-30.3	-91.54 to 30.94	No	ns	0.4544
Con vs. CFA + AAV-shRNA NC	-229.9	-291.1 to -168.6	Yes	****	<0.0001

Dunnett's multiple comparisons test	Mean Diff.	95.00% CI of diff.	Significant?	Summary	Adjusted P Value
CFA + veh vs. Con	230.4	169.1 to 291.6	Yes	####	<0.0001
CFA + veh vs. CFA + AAV-KDM6B shRNA	200.1	138.8 to 261.3	Yes	####	<0.0001
CFA + veh vs. CFA + AAV-shRNA NC	0.4768	-60.76 to 61.72	No	ns	>0.9999

Figure 4D

qPCR	Con	CFA + veh	CFA + AAV-KDM6B shRNA	CFA + AAV-shRNA NC	
	0.98	3.58	1.54	4.95	
	1.03	4.76	2.33	4.79	
	0.97	4.6	2.1	4.68	
	1.05	4.6	1.38	4.87	
	1.01	4.92	1.91	4.85	
Figure 4D KDM6B					
	Con	CFA + veh	CFA + AAV-KDM6B shRNA	CFA + AAV-shRNA NC	
Test for normal distribution					
Shapiro-Wilk test					
W	0.95	0.7779	0.9579	0.9759	
P value	0.7374	0.0528	0.793	0.9118	
Passed normality test (alpha=0.05)?	Yes	Yes	Yes	Yes	
P value summary	ns	ns	ns	ns	
Number of values	5	5	5	5	
**Data exhibits a normal distribution**					
ANOVA summary					
F	163.5				
P value	<0.0001				
P value summary	****				
Significant diff. among means (P < 0.05)?	Yes				
R squared	0.9684				
Dunnett's multiple comparisons test	Mean Diff.	95.00% CI of diff.	Significant?	Summary	Adjusted P Value
Con vs. CFA + veh	-3.484	-4.029 to -2.939	Yes	****	<0.0001

Con vs. CFA + AAV-KDM6B shRNA	-0.844	-1.389 to -0.2989	Yes	**	0.0027
Con vs. CFA + AAV-shRNA NC	-3.82	-4.365 to -3.275	Yes	****	<0.0001
Dunnett's multiple comparisons test	Mean Diff.	95.00% CI of diff.	Significant?	Summary	Adjusted P Value
CFA + veh vs. Con	3.484	2.939 to 4.029	Yes	####	<0.0001
CFA + veh vs. CFA + AAV-KDM6B shRNA	2.64	2.095 to 3.185	Yes	####	<0.0001
CFA + veh vs. CFA + AAV-shRNA NC	-0.336	-0.8811 to 0.2091	No	ns	0.2902
Figure 4D					
TNF-α qPCR	Con	CFA + veh	CFA + AAV-KDM6B shRNA	CFA + AAV-shRNA NC	
	1.1	4.85	1.56	4.62	
	1.09	4.55	1.93	4.77	
	1.08	4.62	1.92	4.6	
	0.92	4.5	1.07	4.86	
	1.02	4.99	1.37	4.83	
Figure 4D TNF-α					
	Con	CFA + veh	CFA + AAV-KDM6B shRNA	CFA + AAV-shRNA NC	
Test for normal distribution					
Shapiro-Wilk test					
W	0.8292	0.9031	0.915	0.8714	
P value	0.1372	0.427	0.498	0.2721	
Passed normality test (alpha=0.05)?	Yes	Yes	Yes	Yes	
P value summary	ns	ns	ns	ns	
Number of values	5	5	5	5	
**Data exhibits a normal distribution**					
ANOVA summary					
F	394.2				
P value	<0.0001				
P value summary	****				
Significant diff. among means (P < 0.05)?	Yes				
R squared	0.9867				

Dunnett's multiple comparisons test	Mean Diff.	95.00% CI of diff.	Significant?	Summary	Adjusted P Value
Con vs. CFA + veh	-3.66	-4.026 to -3.294	Yes	****	<0.0001
Con vs. CFA + AAV-KDM6B shRNA	-0.528	-0.8941 to -0.1619	Yes	**	0.0049
Con vs. CFA + AAV-shRNA NC	-3.694	-4.060 to -3.328	Yes	****	<0.0001
Dunnett's multiple comparisons test	Mean Diff.	95.00% CI of diff.	Significant?	Summary	Adjusted P Value
CFA + veh vs. Con	3.66	3.294 to 4.026	Yes	####	<0.0001
CFA + veh vs. CFA + AAV-KDM6B shRNA	3.132	2.766 to 3.498	Yes	####	<0.0001
CFA + veh vs. CFA + AAV-shRNA NC	-0.034	-0.4001 to 0.3321	No	ns	0.99

Figure 4G

Figure 4G PWT	BL	1/4	1	3	5	7	10
Con	25.368	25.368	25.368	25.368	25.368	17.81	25.368
	25.368	25.368	17.81	17.81	17.81	25.368	25.368
	17.81	25.368	25.368	25.368	25.368	25.368	25.368
	25.368	25.368	25.368	25.368	25.368	25.368	25.368
	25.368	17.81	25.368	25.368	25.368	25.368	25.368
	25.368	25.368	25.368	25.368	25.368	25.368	17.81
	25.368	17.81	20.81	25.368	17.81	25.368	17.81
	25.368	25.368	17.81	25.368	25.368	25.368	25.368
CFA	25.368	4.338	4.338	2.805	2.805	4.338	4.338
	25.368	2.338	4.338	4.338	6.736	4.338	6.736
	25.368	4.338	6.736	4.338	6.736	6.736	11.691
	25.368	3.338	6.736	6.736	4.338	6.736	4.338
	25.368	3.338	2.805	4.338	6.736	6.736	6.736
	25.368	6.736	4.338	6.736	6.736	6.736	6.736
	25.368	4.338	4.338	6.736	4.338	11.691	6.736
	25.368	2.338	4.338	4.338	6.736	4.338	6.736
CFA + AAV-KDM6B shRNA	17.81	6.736	6.736	6.736	11.978	11.978	17.81
	25.368	6.736	6.736	6.736	4.338	4.338	6.736
	25.368	6.736	11.978	6.736	11.978	11.978	11.691
	25.368	11.978	17.81	17.81	17.81	17.81	17.81
	25.368	11.978	11.978	17.81	17.81	17.81	17.81
	25.368	11.978	6.736	11.978	6.736	6.736	11.691
	25.368	6.736	6.736	6.736	6.736	6.736	11.691

	25.368	11.978	6.736	11.978	17.81	17.81	11.691
CFA + AAV-shRNA NC	25.368	4.338	6.736	2.805	6.736	4.338	2.805
	25.368	4.338	4.338	2.805	4.338	6.736	4.338
	25.368	4.338	2.805	2.805	4.338	6.736	6.736
	17.81	4.338	4.338	4.338	4.338	6.736	6.736
	25.368	4.338	4.338	2.805	4.338	4.338	6.736
	25.368	6.736	4.338	6.736	4.338	11.978	11.691
	25.368	4.338	6.736	4.338	6.736	4.338	4.338
	17.81	2.805	2.805	6.736	2.805	2.805	6.736
Figure 4G BL PWT							
	Sham	CFA + veh	CFA + AAV-KDM6B shRNA	CFA + AAV-shRNA NC			
Test for normal distribution							
Shapiro-Wilk test							
W	0.4184	Invalid input data	0.4184	0.5659			
P value	<0.0001		<0.0001	<0.0001			
Passed normality test (alpha=0.05)?	No		No	No			
P value summary	****		****	****			
Number of values	8	8	8	8			
**Data does not exhibit a normal distribution, so use a non-parametric equivalent-the Kruskal-Wallis test.**							
Kruskal-Wallis test							
P value	0.5291						
Exact or approximate P value?	Approximate						
P value summary	ns						
Do the medians vary signif. (P < 0.05)?	No						
Number of groups	4						
Kruskal-Wallis statistic	2.214						
Dunn's multiple comparisons test	Mean rank diff.	Significant?	Summary	Adjusted P Value			
Con vs. CFA + veh	-2	No	ns	>0.9999			
Con vs. CFA + AAV-KDM6B shRNA	0	No	ns	>0.9999			

Con vs. CFA + AAV-shRNA NC	2	No	ns	>0.9999			
Figure 4G 6h PWT							
	Sham	CFA + veh	CFA + AAV-KDM6B shRNA	CFA + AAV-shRNA NC			
Test for normal distribution							
Shapiro-Wilk test							
W	0.5659	0.8796	0.6647	0.7065			
P value	<0.0001	0.1867	0.0009	0.0027			
Passed normality test (alpha=0.05)?	No	Yes	No	No			
P value summary	****	ns	***	**			
Number of values	8	8	8	8			
**Data does not exhibit a normal distribution, so use a non-parametric equivalent-the Kruskal-Wallis test.**							
Kruskal-Wallis test							
P value	<0.0001						
Exact or approximate P value?	Approximate						
P value summary	****						
Do the medians vary signif. (P < 0.05)?	Yes						
Number of groups	4						
Kruskal-Wallis statistic	26.42						
Dunn's multiple comparisons test	Mean rank diff.	Significant?	Summary	Adjusted P Value			
CFA + veh vs. Con	-21.06	Yes	****	<0.0001			
CFA + veh vs. CFA + AAV-KDM6B shRNA	-12.56	Yes	*	0.019			
CFA + veh vs. CFA + AAV-shRNA NC	-2.625	No	ns	>0.9999			
Figure 4G 1d PWT							
	Sham	CFA + veh	CFA + AAV-KDM6B shRNA	CFA + AAV-shRNA NC			
Test for normal distribution							

Shapiro-Wilk test							
W	0.6954	0.786	0.7201	0.8288			
P value	0.002	0.0202	0.0038	0.0577			
Passed normality test (alpha=0.05)?	No	No	No	Yes			
P value summary	**	*	**	ns			
Number of values	8	8	8	8			
**Data does not exhibit a normal distribution, so use a non-parametric equivalent-the Kruskal-Wallis test.**							
Kruskal-Wallis test							
P value	<0.0001						
Exact or approximate P value?	Approximate						
P value summary	****						
Do the medians vary signif. (P < 0.05)?	Yes						
Number of groups	4						
Kruskal-Wallis statistic	24.7						
Dunn's multiple comparisons test	Mean rank diff.	Significant?	Summary	Adjusted P Value			
CFA + veh vs. Con	-18.88	Yes	***	0.0001			
CFA + veh vs. CFA + AAV-KDM6B shRNA	-9.875	No	ns	0.0925			
CFA + veh vs. CFA + AAV-shRNA NC	0.75	No	ns	>0.9999			
Figure 4G 3d PWT							
	Sham	CFA + veh	CFA + AAV-KDM6B shRNA	CFA + AAV-shRNA NC			
Test for normal distribution							
Shapiro-Wilk test							
W	0.4184	0.8112	0.78	0.7649			
P value	<0.0001	0.0377	0.0174	0.0119			
Passed normality test (alpha=0.05)?	No	No	No	No			
P value summary	****	*	*	*			
Number of values	8	8	8	8			
**Data does not exhibit a normal distribution, so use a non-parametric equivalent-the Kruskal-Wallis test.**							

Kruskal-Wallis test							
P value	<0.0001						
Exact or approximate P value?	Approximate						
P value summary	****						
Do the medians vary signif. (P < 0.05)?	Yes						
Number of groups	4						
Kruskal-Wallis statistic	24.95						
Dunn's multiple comparisons test	Mean rank diff.	Significant?	Summary	Adjusted P Value			
CFA + veh vs. Con	-17.75	Yes	***	0.0003			
CFA + veh vs. CFA + AAV-KDM6B shRNA	-8.75	No	ns	0.1695			
CFA + veh vs. CFA + AAV-shRNA NC	3	No	ns	>0.9999			
Figure 4G 5d PWT							
	Sham	CFA + veh	CFA + AAV-KDM6B shRNA	CFA + AAV-shRNA NC			
Test for normal distribution							
Shapiro-Wilk test							
W	0.5659	0.7257	0.8582	0.786			
P value	<0.0001	0.0044	0.1151	0.0202			
Passed normality test (alpha=0.05)?	No	No	Yes	No			
P value summary	****	**	ns	*			
Number of values	8	8	8	8			
**Data does not exhibit a normal distribution, so use a non-parametric equivalent-the Kruskal-Wallis test.**							
Kruskal-Wallis test							
P value	<0.0001						
Exact or approximate P value?	Approximate						
P value summary	****						
Do the medians vary signif. (P < 0.05)?	Yes						
Number of groups	4						
Kruskal-Wallis statistic	22.95						

Dunn's multiple comparisons test	Mean rank diff.	Significant?	Summary	Adjusted P Value			
CFA + veh vs. Con	-16.94	Yes	***	0.0006			
CFA + veh vs. CFA + AAV-KDM6B shRNA	-7.5	No	ns	0.3038			
CFA + veh vs. CFA + AAV-shRNA NC	3.188	No	ns	>0.9999			
Figure 4G 7d PWT							
	Sham	CFA + veh	CFA + AAV-KDM6B shRNA	CFA + AAV-shRNA NC			
Test for normal distribution							
Shapiro-Wilk test							
W	0.4184	0.7691	0.8582	0.8435			
P value	<0.0001	0.0132	0.1151	0.0818			
Passed normality test (alpha=0.05)?	No	No	Yes	Yes			
P value summary	****	*	ns	ns			
Number of values	8	8	8	8			
**Data does not exhibit a normal distribution, so use a non-parametric equivalent-the Kruskal-Wallis test.**							
Kruskal-Wallis test							
P value	<0.0001						
Exact or approximate P value?	Approximate						
P value summary	****						
Do the medians vary signif. (P < 0.05)?	Yes						
Number of groups	4						
Kruskal-Wallis statistic	21.54						
Dunn's multiple comparisons test	Mean rank diff.	Significant?	Summary	Adjusted P Value			
CFA + veh vs. Con	-17.69	Yes	***	0.0003			
CFA + veh vs. CFA + AAV-KDM6B shRNA	-7.063	No	ns	0.37			
CFA + veh vs. CFA + AAV-shRNA NC	1.25	No	ns	>0.9999			

Figure 4G 10d PWT							
	Sham	CFA + veh	CFA + AAV-KDM6B shRNA	CFA + AAV-shRNA NC			
Test for normal distribution							
Shapiro-Wilk test							
W	0.5659	0.7522	0.8235	0.8641			
P value	<0.0001	0.0086	0.0508	0.1318			
Passed normality test (alpha=0.05)?	No	No	Yes	Yes			
P value summary	****	**	ns	ns			
Number of values	8	8	8	8			
**Data does not exhibit a normal distribution, so use a non-parametric equivalent-the Kruskal-Wallis test.**							
Kruskal-Wallis test							
P value	<0.0001						
Exact or approximate P value?	Approximate						
P value summary	****						
Do the medians vary signif. (P < 0.05)?	Yes						
Number of groups	4						
Kruskal-Wallis statistic	24.18						
Dunn's multiple comparisons test	Mean rank diff.	Significant?	Summary	Adjusted P Value			
CFA + veh vs. Con	-18.38	Yes	***	0.0002			
CFA + veh vs. CFA + AAV-KDM6B shRNA	-9.813	No	ns	0.0959			
CFA + veh vs. CFA + AAV-shRNA NC	1.188	No	ns	>0.9999			
Figure 4G PWT CFA: vs Baseline							
	BL	1/4	1	3	5	7	10
Test for normal distribution							
Shapiro-Wilk test							
W	Invalid input data	0.8796	0.786	0.8112	0.7257	0.7691	0.7522

P value		0.1867	0.0202	0.0377	0.0044	0.0132	0.0086
Passed normality test (alpha=0.05)?		Yes	No	No	No	No	No
P value summary		ns	*	*	**	*	**
Number of values	8	8	8	8	8	8	8
**Data does not exhibit a normal distribution, so use a non-parametric equivalent-the Kruskal-Wallis test.**							
Kruskal-Wallis test							
P value	<0.0001						
Exact or approximate P value?	Approximate						
P value summary	****						
Do the medians vary signif. (P < 0.05)?	Yes						
Number of groups	7						
Kruskal-Wallis statistic	31.16						
Dunn's multiple comparisons test	Mean rank diff.	Significant?	Summary	Adjusted P Value			
BL vs. 1/4	39.56	Yes	####	<0.0001			
BL vs. 1	32.25	Yes	###	0.0002			
BL vs. 3	29.81	Yes	###	0.0008			
BL vs. 5	24.94	Yes	##	0.0082			
BL vs. 7	21.94	Yes	#	0.0292			
BL vs. 10	19.5	No	ns	0.0738			
Two-way ANOVA	Ordinary						
Alpha	0.05						
Source of Variation	% of total variation	P value	P value summary	Significant?			
Interaction	10.66	<0.0001	****	Yes			
Row Factor	22.06	<0.0001	****	Yes			
Column Factor	54.11	<0.0001	****	Yes			

Figure 4H

Figure 4H PWL	BL	1/4	1	3	5	7	10
Con	12.967	12.8	13.1	12.533	13.267	13.767	13.6
	13.633	13.767	13.5	13.4	12.933	14.533	13.567

	13.133	12.6	12.2	12.7	13.333	12.433	13.767
	13.1	13.567	12.567	13.233	12.2	13.967	12.933
	13.5	13.467	13.167	13.033	12.433	13.433	12.833
	12.467	13.767	13.6	12.967	13.2	13.833	12.767
	12.467	13.733	12.833	13.733	13.767	13.5	13.133
	12.7	12.533	12.2	12.567	13.033	12.433	13.1
CFA	12.733	2	3.868	2.567	3	1.333	3.333
	12.233	1.833	3.067	4.067	4.333	4	4.167
	13.033	1.6	2.733	2.84	3.567	3.6	3.8
	13.6	2.067	3.067	3.067	3.6	4.267	3.933
	12.933	2.3	2	3.913	3.667	4.5	4.667
	13.2	2.033	2.967	2.076	3.5	2.867	3.933
	12.767	2.3	3.067	3.221	3.433	4	4.133
	12.5	1.5	2.5	2.349	3.467	4.4	4
CFA + AAV-KDM6B shRNA	12.6	4.3	5.83	5.967	5.133	6.533	7.833
	11.933	5.333	5.767	5.769	6.167	6.933	5.033
	12.9	5.267	6.34	5.34	7.233	8.567	7.667
	13.133	6.367	7.183	5.767	8.6	7.733	8.233
	14	6.733	6.133	6.133	7.967	6.633	8.767
	12.8	7.1	6.109	5.933	6.5	6.167	7.25
	14.133	4.833	6.233	7.233	5.867	7	6.833
	12.433	7.033	7.02	8.033	8.133	8	8.5
CFA + AAV-shRNA NC	13.833	2.1	3.968	2.667	2.433	3.3	2.433
	13.6	2.133	3.167	4.167	4.4	4.433	4.267
	11.9	3.2	2.833	6.44	3.7	3.667	3.9
	13.467	2.167	2.967	3.167	4.367	3.7	5.033
	12.733	2.4	2.1	4.013	4.1	3.767	4.767
	13.067	2.133	3.067	2.176	2.967	3.6	4.033
	13.467	2.4	3.167	3.321	4.1	3.533	4.233
	11.867	1.6	2.6	2.449	4.5	5.467	4.1
Figure 4H BL PWL							
	Sham	CFA + veh	CFA + AAV-KDM7B shRNA	CFA + AAV-shRNA NC			
Test for normal distribution							
Shapiro-Wilk test							
W	0.9305	0.9911	0.934	0.8693			
P value	0.5204	0.9965	0.5528	0.1483			
Passed normality test (alpha=0.05)?	Yes	Yes	Yes	Yes			
P value summary	ns	ns	ns	ns			

Number of values	8	8	8	8			
**Data exhibits a normal distribution**							
ANOVA summary							
F	0.07377						
P value	0.9736						
P value summary	ns						
Significant diff. among means (P < 0.05)?	No						
R squared	0.007842						
Dunnett's multiple comparisons test	Mean Diff.	95.00% CI of diff.	Significant?	Summary	Adjusted P Value		
CFA + veh vs. Con	-0.121	-0.8854 to 0.6434	No	ns	0.9605		
CFA + veh vs. CFA + AAV-KDM6B shRNA	-0.1166	-0.8810 to 0.6477	No	ns	0.9643		
CFA + veh vs. CFA + AAV-shRNA NC	-0.1169	-0.8812 to 0.6475	No	ns	0.9641		
Figure 4H 6h PWL							
	Sham	CFA + veh	CFA + AAV-KDM7B shRNA	CFA + AAV-shRNA NC			
Test for normal distribution							
Shapiro-Wilk test							
W	0.8061	0.9231	0.9091	0.8675			
P value	0.0333	0.4551	0.3478	0.1424			
Passed normality test (alpha=0.05)?	No	Yes	Yes	Yes			
P value summary	*	ns	ns	ns			
Number of values	8	8	8	8			
**Data does not exhibit a normal distribution, so use a non-parametric equivalent-the Kruskal-Wallis test.**							
Kruskal-Wallis test							
P value	<0.0001						
Exact or approximate P value?	Approximate						

P value summary	****						
Do the medians vary signif. (P < 0.05)?	Yes						
Number of groups	4						
Kruskal-Wallis statistic	27.08						
Dunn's multiple comparisons test	Mean rank diff.	Significant?	Summary	Adjusted P Value			
CFA + veh vs. Con	-22.19	Yes	****	<0.0001			
CFA + veh vs. CFA + AAV-KDM6B shRNA	-14.19	Yes	**	0.0074			
CFA + veh vs. CFA + AAV-shRNA NC	-4.375	No	ns	>0.9999			
Figure 4H 1d PWL							
	Sham	CFA + veh	CFA + AAV-KDM7B shRNA	CFA + AAV-shRNA NC			
Test for normal distribution							
Shapiro-Wilk test							
W	0.9239	0.9331	0.8762	0.9464			
P value	0.4624	0.5446	0.1731	0.6744			
Passed normality test (alpha=0.05)?	Yes	Yes	Yes	Yes			
P value summary	ns	ns	ns	ns			
Number of values	8	8	8	8			
**Data exhibits a normal distribution**							
ANOVA summary							
F	620.6						
P value	<0.0001						
P value summary	****						
Significant diff. among means (P < 0.05)?	Yes						
R squared	0.9852						
Dunnett's multiple comparisons test	Mean Diff.	95.00% CI of diff.	Significant?	Summary	Adjusted P Value		
CFA + veh vs. Con	-9.987	-10.65 to -9.326	Yes	****	<0.0001		

CFA + veh vs. CFA + AAV-KDM6B shRNA	-3.418	-4.079 to -2.757	Yes	****	<0.0001		
CFA + veh vs. CFA + AAV-shRNA NC	-0.075	-0.7362 to 0.5862	No	ns	0.9846		
Figure 4H 3d PWL							
	Sham	CFA + veh	CFA + AAV-KDM7B shRNA	CFA + AAV-shRNA NC			
Test for normal distribution							
Shapiro-Wilk test							
W	0.9477	0.9494	0.828	0.8707			
P value	0.688	0.705	0.0566	0.1532			
Passed normality test (alpha=0.05)?	Yes	Yes	Yes	Yes			
P value summary	ns	ns	ns	ns			
Number of values	8	8	8	8			
**Data exhibits a normal distribution**							
ANOVA summary							
F	202.3						
P value	<0.0001						
P value summary	****						
Significant diff. among means (P < 0.05)?	Yes						
R squared	0.9559						
Dunnett's multiple comparisons test	Mean Diff.	95.00% CI of diff.	Significant?	Summary	Adjusted P Value		
CFA + veh vs. Con	-10.01	-11.14 to -8.873	Yes	****	<0.0001		
CFA + veh vs. CFA + AAV-KDM6B shRNA	-3.259	-4.395 to -2.124	Yes	****	<0.0001		
CFA + veh vs. CFA + AAV-shRNA NC	-0.5375	-1.673 to 0.5979	No	ns	0.5128		
Figure 4H 5d PWL							

	Sham	CFA + veh	CFA + AAV-KDM7B shRNA	CFA + AAV-shRNA NC			
Test for normal distribution							
Shapiro-Wilk test							
W	0.9537	0.8656	0.9553	0.8455			
P value	0.748	0.1366	0.7643	0.0858			
Passed normality test (alpha=0.05)?	Yes	Yes	Yes	Yes			
P value summary	ns	ns	ns	ns			
Number of values	8	8	8	8			
**Data exhibits a normal distribution**							
ANOVA summary							
F	252.3						
P value	<0.0001						
P value summary	****						
Significant diff. among means (P < 0.05)?	Yes						
R squared	0.9643						
Dunnett's multiple comparisons test	Mean Diff.	95.00% CI of diff.	Significant?	Summary	Adjusted P Value		
CFA + veh vs. Con	-9.45	-10.42 to -8.478	Yes	****	<0.0001		
CFA + veh vs. CFA + AAV-KDM6B shRNA	-3.379	-4.351 to -2.407	Yes	****	<0.0001		
CFA + veh vs. CFA + AAV-shRNA NC	-0.25	-1.222 to 0.7224	No	ns	0.8595		
Figure 4H 7d PWL							
	Sham	CFA + veh	CFA + AAV-KDM7B shRNA	CFA + AAV-shRNA NC			
Test for normal distribution							
Shapiro-Wilk test							
W	0.9044	0.8037	0.9421	0.7723			
P value	0.3161	0.0314	0.6315	0.0144			

Passed normality test (alpha=0.05)?	Yes	No	Yes	No			
P value summary	ns	*	ns	*			
Number of values	8	8	8	8			
**Data does not exhibit a normal distribution, so use a non-parametric equivalent-the Kruskal-Wallis test.**							
Kruskal-Wallis test							
P value	<0.0001						
Exact or approximate P value?	Approximate						
P value summary	****						
Do the medians vary signif. (P < 0.05)?	Yes						
Number of groups	4						
Kruskal-Wallis statistic	26.2						
Dunn's multiple comparisons test	Mean rank diff.	Significant?	Summary	Adjusted P Value			
CFA + veh vs. Con	-19.81	Yes	****	<0.0001			
CFA + veh vs. CFA + AAV-KDM6B shRNA	-11.81	Yes	*	0.0353			
CFA + veh vs. CFA + AAV-shRNA NC	0.375	No	ns	>0.9999			
Figure 4H 10d PWL							
	Sham	CFA + veh	CFA + AAV-KDM7B shRNA	CFA + AAV-shRNA NC			
Test for normal distribution							
Shapiro-Wilk test							
W	0.9044	0.8037	0.9421	0.7723			
P value	0.3161	0.0314	0.6315	0.0144			
Passed normality test (alpha=0.05)?	Yes	No	Yes	No			
P value summary	ns	*	ns	*			
Number of values	8	8	8	8			
**Data does not exhibit a normal distribution, so use a non-parametric equivalent-the Kruskal-Wallis test.**							
Kruskal-Wallis test							
P value	<0.0001						
Exact or approximate P	Approxi						

value?	mate						
P value summary	****						
Do the medians vary signif. (P < 0.05)?	Yes						
Number of groups	4						
Kruskal-Wallis statistic	26.2						
Dunn's multiple comparisons test	Mean rank diff.	Significant?	Summary	Adjusted P Value			
CFA + veh vs. Con	-19.81	Yes	****	<0.0001			
CFA + veh vs. CFA + AAV-KDM6B shRNA	-11.81	Yes	*	0.0353			
CFA + veh vs. CFA + AAV-shRNA NC	0.375	No	ns	>0.9999			
Figure 4H PWL CFA: vs Baseline							
	BL	1/4	1	3	5	7	10
Test for normal distribution							
Shapiro-Wilk test							
W	0.9911	0.9231	0.9331	0.9494	0.8656	0.8037	0.9404
P value	0.9965	0.4551	0.5446	0.705	0.1366	0.0314	0.6149
Passed normality test (alpha=0.05)?	Yes	Yes	Yes	Yes	Yes	No	Yes
P value summary	ns	ns	ns	ns	ns	*	ns
Number of values	8	8	8	8	8	8	8
**Data does not exhibit a normal distribution, so use a non-parametric equivalent-the Kruskal-Wallis test.**							
Kruskal-Wallis test							
P value	<0.0001						
Exact or approximate P value?	Approximate						
P value summary	****						
Do the medians vary signif. (P < 0.05)?	Yes						
Number of groups	7						
Kruskal-Wallis statistic	39.73						
Dunn's multiple comparisons test	Mean rank diff.	Significant?	Summary	Adjusted P Value			
BL vs. 1/4	46.19	Yes	####	<0.0001			

BL vs. 1	33.75	Yes	###	0.0002			
BL vs. 3	30.94	Yes	###	0.0009			
BL vs. 5	22.94	Yes	#	0.0294			
BL vs. 7	19.56	No	ns	0.0985			
BL vs. 10	14.63	No	ns	0.4369			
Two-way ANOVA	Ordinary						
Alpha	0.05						
Source of Variation	% of total variation	P value	P value summary	Significant?			
Interaction	4.742	0.0052	**	Yes			
Row Factor	71.04	<0.0001	****	Yes			
Column Factor	11.15	<0.0001	****	Yes			

Figure 4I

Western blot KDM6B	Con	CFA+veh	CFA+AAV-KDM6BshRNA	CFA+AAV-shRNANC	
	104.87	225.28	113.77	261.35	
	87.21	374.38	107.42	313.92	
	97.22	279.43	99.87	357.16	
	110.29	374.14	95.75	283.17	
	100.41	364.14	99.32	283.17	
Figure 4I KDM6B					
	Sham	CFA + veh	CFA + AAV-KDM6B shRNA	CFA + AAV-shRNA NC	
Test for normal distribution					
Shapiro-Wilk test					
W	0.9811	0.8086	0.9191	0.9156	
P value	0.9403	0.0951	0.5243	0.5023	
Passed normality test (alpha=0.05)?	Yes	Yes	Yes	Yes	
P value summary	ns	ns	ns	ns	
Number of values	5	5	5	5	
**Data exhibits a normal distribution**					
ANOVA summary					
F	48.46				

P value	<0.0001				
P value summary	****				
Significant diff. among means (P < 0.05)?	Yes				
R squared	0.9009				
Dunnett's multiple comparisons test	Mean Diff.	95.00% CI of diff.	Significant?	Summary	Adjusted P Value
Con vs. CFA+veh	-223.5	-287.5 to -159.4	Yes	****	<0.0001
Con vs. CFA+AAV-KDM6BshRNA	-3.225	-67.29 to 60.84	No	ns	0.9983
Con vs. CFA+AAV-shRNANC	-199.8	-263.8 to -135.7	Yes	****	<0.0001
Dunnett's multiple comparisons test	Mean Diff.	95.00% CI of diff.	Significant?	Summary	Adjusted P Value
CFA+veh vs. Con	223.5	159.4 to 287.5	Yes	####	<0.0001
CFA+veh vs. CFA+AAV-KDM6BshRNA	220.3	156.2 to 284.3	Yes	####	<0.0001
CFA+veh vs. CFA+AAV-shRNANC	23.72	-40.35 to 87.79	No	ns	0.6612
Figure 4I					
WB TNF-alpha	Con	CFA+veh	CFA+AAV-KDM6BshRNA	CFA+AAV-shRNANC	
	97.10	324.12	98.39	296.46	
	89.04	296.78	111.93	299.37	
	110.16	410.26	116.59	291.16	
	106.98	282.32	118.96	384.99	
Figure 4I TNF-α					
	Sham	CFA + veh	CFA + AAV-KDM 6B shRNA	CFA + AAV-shRN A NC	
Test for normal distribution					
Shapiro-Wilk test					
W	0.9365	0.9448	0.8021	0.7356	
P value	0.641	0.7002	0.0843	0.0218	
Passed normality test (alpha=0.05)?	Yes	Yes	Yes	No	
P value summary	ns	ns	ns	*	

Number of values	5	5	5	5	
**Data does not exhibit a normal distribution, so use a non-parametric equivalent-the Kruskal-Wallis test.**					
Kruskal-Wallis test					
P value	0.0013				
Exact or approximate P value?	Approximate				
P value summary	**				
Do the medians vary signif. (P < 0.05)?	Yes				
Number of groups	4				
Kruskal-Wallis statistic	15.78				
Dunn's multiple comparisons test	Mean rank diff.	Significant?	Summary	Adjusted P Value	
Con vs. CFA+veh	-13	Yes	**	0.0015	
Con vs. CFA+AAV-KDM6BshRNA	-4.2	No	ns	0.785	
Con vs. CFA+AAV-shRNANC	-11.2	Yes	**	0.0083	
Dunnett's multiple comparisons test	Mean Diff.	Significant?	Summary	Adjusted P Value	
CFA+veh vs. CFA+AAV-KDM6BshRNA	221.3	Yes	****	<0.0001	
CFA+veh vs. CFA+AAV-shRNANC	24.48	No	ns	0.5249	

Figure 4J

KDM6B qPCR	Con	CFA+veh	CFA+AAV-KDM6BshRNA	CFA+AAV-shRNANC	
	1.25	4.60	0.92	5.07	
	0.85	4.28	1.01	4.65	
	0.98	5.05	0.99	4.40	
	0.91	4.09	0.90	4.91	
Figure 4J KDM6B					
	Sham	CFA + veh	CFA + AAV-KDM6B shRNA	CFA + AAV-shRNA NC	
Test for normal distribution					
Shapiro-Wilk test					
W	0.8997	0.8965	0.9326	0.9615	
P value	0.4083	0.3909	0.6139	0.8184	

Passed normality test (alpha=0.05)?	Yes	Yes	Yes	Yes	
P value summary	ns	ns	ns	ns	
Number of values	5	5	5	5	
**Data exhibits a normal distribution**					
ANOVA summary					
F	221.1				
P value	<0.0001				
P value summary	****				
Significant diff. among means (P < 0.05)?	Yes				
R square	0.9764				
Dunnett's multiple comparisons test	Mean Diff.	95.00% CI of diff.	Significant?	Summary	Adjusted P Value
Con vs. CFA+veh	-3.447	-3.950 to -2.944	Yes	****	<0.0001
Con vs. CFA+AAV-KDM6BshRNA	0.0254	-0.4778 to 0.5286	No	ns	0.9983
Con vs. CFA+AAV-shRNANC	-3.593	-4.096 to -3.090	Yes	****	<0.0001
Dunnett's multiple comparisons test	Mean Diff.	95.00% CI of diff.	Significant?	Summary	Adjusted P Value
CFA+veh vs. Con	3.447	2.944 to 3.950	Yes	####	<0.0001
CFA+veh vs. CFA+AAV-KDM6BshRNA	3.473	2.969 to 3.976	Yes	####	<0.0001
CFA+veh vs. CFA+AAV-shRNANC	-0.1456	-0.6488 to 0.3576	No	ns	0.796
Figure 4J TNF-α qPCR	Con	CFA+veh	CFA+AAV-KDM6BshRNA	CFA+AAV-shRNANC	
	1.19	4.11	0.98	4.78	
	0.91	4.65	0.94	4.65	
	1.06	4.80	1.05	4.66	
	0.82	4.22	1.09	4.35	
	1.03	4.62	1.04	4.47	
Figure 4J TNF-α					
	Sham	CFA + veh	CFA + AAV-KDM	CFA + AAV-shRN	

			6B shRNA	A NC	
Test for normal distribution					
Shapiro-Wilk test					
W	0.9846	0.8892	0.9759	0.9447	
P value	0.9578	0.3532	0.9117	0.6994	
Passed normality test (alpha=0.05)?	Yes	Yes	Yes	Yes	
P value summary	ns	ns	ns	ns	
Number of values	5	5	5	5	
**Data exhibits a normal distribution**					
ANOVA summary					
F	575.4				
P value	<0.0001				
P value summary	****				
Significant diff. among means (P < 0.05)?	Yes				
R square	0.9908				
Dunnett's multiple comparisons test	Mean Diff.	95.00% CI of diff.	Significant?	Summary	Adjusted P Value
Con vs. CFA+veh	-3.481	-3.792 to -3.170	Yes	****	<0.0001
Con vs. CFA+AAV-KDM6BshRNA	-0.0202	-0.3310 to 0.2906	No	ns	0.9965
Con vs. CFA+AAV-shRNANC	-3.58	-3.891 to -3.269	Yes	****	<0.0001
Dunnett's multiple comparisons test	Mean Diff.	95.00% CI of diff.	Significant?	Summary	Adjusted P Value
CFA+veh vs. Con	3.481	3.170 to 3.792	Yes	####	<0.0001
CFA+veh vs. CFA+AAV-KDM6BshRNA	3.461	3.150 to 3.772	Yes	####	<0.0001
CFA+veh vs. CFA+AAV-shRNANC	-0.099	-0.4098 to 0.2118	No	ns	0.7489

Figure 6C

Figure 6C qPCR	Con	CFA	CFA + shRNA		
	1.02	2.82	1.27		
	0.94	2.93	1.37		
	1.04	3.16	1.45		

Figure 6C TNF-α promoter					
	Con	CFA	CFA + AAV-KDM 6B shRNA		
Test for normal distribution					
Shapiro-Wilk test					
W	0.8637	0.96	0.9947		
P value	0.2779	0.6155	0.8608		
Passed normality test (alpha=0.05)?	Yes	Yes	Yes		
P value summary	ns	ns	ns		
Number of values	3	3	3		
**Data exhibits a normal distribution**					
ANOVA summary					
F	253.8				
P value	<0.0001				
P value summary	****				
Significant diff. among means (P < 0.05)?	Yes				
R square	0.9883				
Dunnett's multiple comparisons test	Mean Diff.	95.00% CI of diff.	Significant?	Summary	Adjusted P Value
Con vs. CFA	-1.97	-2.236 to -1.704	Yes	****	<0.0001
Con vs. CFA + shRNA	-0.3627	-0.6291 to -0.09620	Yes	*	0.0142
Dunnett's multiple comparisons test	Mean Diff.	95.00% CI of diff.	Significant?	Summary	Adjusted P Value
CFA vs. Con	1.97	1.704 to 2.236	Yes	####	<0.0001
CFA vs. CFA + shRNA	1.607	1.341 to 1.874	Yes	####	<0.0001

Figure 6D

Figure 6D Western blot	Con	CFA	CFA + shRNA		
	103.19	51.12	60.37		
	99.29	49.50	71.47		
	97.53	41.14	73.06		
Figure 6D H3K27me3					

	Con	CFA	CFA + AAV-KD M6B shRNA		
Test for normal distribution					
Shapiro-Wilk test					
W	1	0.9652	0.8454		
P value	>0.9999	0.6414	0.2283		
Passed normality test (alpha=0.05)?	Yes	Yes	Yes		
P value summary	ns	ns	ns		
Number of values	3	3	3		
**Data exhibits a normal distribution**					
ANOVA summary					
F	74.78				
P value	<0.0001				
P value summary	****				
Significant diff. among means (P < 0.05)?	Yes				
R square	0.9614				
Dunnett's multiple comparisons test	Mean Diff.	95.00% CI of diff.	Significant ?	Summary	Adjusted P Value
Con vs. CFA	52.74	40.31 to 65.18	Yes	****	<0.0001
Con vs. CFA + shRNA	31.7	19.27 to 44.13	Yes	***	0.0006
Dunnett's multiple comparisons test	Mean Diff.	95.00% CI of diff.	Significant ?	Summary	Adjusted P Value
CFA vs. Con	-52.74	-65.18 to -40.31	Yes	####	<0.0001
CFA vs. CFA + shRNA	-21.05	-33.48 to -8.614	Yes	##	0.0051

Figure 6F

Figure 6F qPCR	Con	CFA	CFA + shRNA		
	1.01	2.83	1.45		
	0.91	3.60	1.32		
	1.08	3.32	1.52		
Figure 6F TNF-α promoter					
	Con	CFA	CFA +		

			AAV-KD M6B shRNA		
Test for normal distribution					
Shapiro-Wilk test					
W	0.9569	0.8923	0.9665		
P value	0.6006	0.3615	0.6482		
Passed normality test (alpha=0.05)?	Yes	Yes	Yes		
P value summary	ns	ns	ns		
Number of values	3	3	3		
**Data exhibits a normal distribution**					
ANOVA summary					
F	77.15				
P value	<0.0001				
P value summary	****				
Significant diff. among means (P < 0.05)?	Yes				
R square	0.9626				
Dunnett's multiple comparisons test	Mean Diff.	95.00% CI of diff.	Significant ?	Summary	Adjusted P Value
Con vs. CFA	-2.249	-2.799 to -1.698	Yes	****	<0.0001
Con vs. CFA + shRNA	-0.429	-0.9794 to 0.1214	No	ns	0.1143
Dunnett's multiple comparisons test	Mean Diff.	95.00% CI of diff.	Significant ?	Summary	Adjusted P Value
CFA vs. Con	2.249	1.698 to 2.799	Yes	####	<0.0001
CFA vs. CFA + shRNA	1.82	1.269 to 2.370	Yes	###	0.0002

Figure 6G

Figure 6G	Con	CFA + veh	CFA + shRNA	CFA + shRNA NC	
	100.84	58.74	97.16	63.03	
	99.41	59.55	112.29	68.30	
	95.94	72.56	86.13	58.89	
	101.70	36.21	97.63	53.65	
	102.12	62.04	103.71	60.19	

Figure 6G H3K27me3					
	Con	CFA	CFA + AAV-KD M6B shRNA		
Test for normal distribution					
Shapiro-Wilk test					
W	0.9545	0.8678	0.8422		
P value	0.5894	0.2892	0.2198		
Passed normality test (alpha=0.05)?	Yes	Yes	Yes		
P value summary	ns	ns	ns		
Number of values	3	3	3		
**Data exhibits a normal distribution**					
ANOVA summary					
F	143.7				
P value	<0.0001				
P value summary	****				
Significant diff. among means (P < 0.05)?	Yes				
R square	0.9796				
Dunnett's multiple comparisons test	Mean Diff.	95.00% CI of diff.	Significant ?	Summary	Adjusted P Value
Con vs. CFA	51.19	42.36 to 60.01	Yes	****	<0.0001
Con vs. CFA + shRNA	34.67	25.84 to 43.49	Yes	****	<0.0001
Dunnett's multiple comparisons test	Mean Diff.	95.00% CI of diff.	Significant ?	Summary	Adjusted P Value
CFA vs. Con	-51.19	-60.01 to -42.36	Yes	####	<0.0001
CFA vs. CFA + shRNA	-16.52	-25.34 to -7.697	Yes	##	0.0031

Figure 6H

Figure 6H Western blot	Con	CFA + veh	CFA + shRNA	CFA + shRNA NC	
	105.79	60.35	98.65	62.18	
	101.85	64.82	96.89	62.93	
	96.38	42.85	104.31	63.97	

	96.15	41.23	99.76	45.18	
	99.83	56.01	98.46	68.85	
Figure 6H H3K27me3					
	Con	CFA + veh	CFA + AAV-KDM 6B shRNA	CFA + AAV-shRN A NC	
Test for normal distribution					
Shapiro-Wilk test					
W	0.8716	0.8823	0.9751	0.9906	
P value	0.2728	0.3198	0.9068	0.9816	
Passed normality test (alpha=0.05)?	Yes	Yes	Yes	Yes	
P value summary	ns	ns	ns	ns	
Number of values	5	5	5	5	
**Data exhibits a normal distribution**					
ANOVA summary					
F	35.85				
P value	<0.0001				
P value summary	****				
Significant diff. among means (P < 0.05)?	Yes				
R square	0.8705				
Dunnett's multiple comparisons test	Mean Diff.	95.00% CI of diff.	Significant?	Summary	Adjusted P Value
Con vs. CFA + veh	42.18	27.88 to 56.48	Yes	****	<0.0001
Con vs. CFA + shRNA	0.616	-13.68 to 14.91	No	ns	0.999
Con vs. CFA + shRNA NC	39.19	24.89 to 53.48	Yes	****	<0.0001
Dunnett's multiple comparisons test	Mean Diff.	95.00% CI of diff.	Significant?	Summary	Adjusted P Value
CFA + veh vs. Con	-42.18	-56.48 to -27.88	Yes	####	<0.0001
CFA + veh vs. CFA + shRNA	-41.56	-55.86 to -27.27	Yes	####	<0.0001
CFA + veh vs. CFA + shRNA NC	-2.992	-17.29 to 11.30	No	ns	0.906

Figure 6I

Western blot	Con	CFA + veh	CFA + shRNA	CFA + shRNA NC	
	105.79	60.35	98.65	62.18	
	101.85	64.82	96.89	62.93	
	96.38	42.85	104.31	63.97	
	96.15	41.23	99.76	45.18	
	99.83	56.01	98.46	68.85	
Figure 6I H3K27me3					
	Con	CFA + veh	CFA + AAV-KDM 6B shRNA	CFA + AAV-shRN A NC	
Test for normal distribution					
Shapiro-Wilk test					
W	0.9201	0.8915	0.8665	0.8036	
P value	0.5306	0.3646	0.2524	0.0867	
Passed normality test (alpha=0.05)?	Yes	Yes	Yes	Yes	
P value summary	ns	ns	ns	ns	
Number of values	5	5	5	5	
**Data exhibits a normal distribution**					
ANOVA summary					
F	57.73				
P value	<0.0001				
P value summary	****				
Significant diff. among means (P < 0.05)?	Yes				
R square	0.9154				
Dunnett's multiple comparisons test	Mean Diff.	95.00% CI of diff.	Significant?	Summary	Adjusted P Value
Con vs. CFA + veh	46.95	34.88 to 59.01	Yes	****	<0.0001
Con vs. CFA + shRNA	0.386	-11.68 to 12.45	No	ns	0.9996
Con vs. CFA + shRNA NC	39.38	27.31 to 51.44	Yes	****	<0.0001
Dunnett's multiple comparisons test	Mean Diff.	95.00% CI of diff.	Significant?	Summary	Adjusted P Value
CFA + veh vs. Con	-46.95	-59.01 to -34.88	Yes	####	<0.0001
CFA + veh vs. CFA + shRNA	-46.56	-58.63 to -34.50	Yes	####	<0.0001

CFA + veh vs. CFA + shRNA NC	-7.57	-19.63 to 4.495	No	ns	0.2776

Figure 6J

Western blot	Con	1/4	1	3	7	14	21
	97.46	196.69	303.81	168.51	165.39	147.02	103.63
	103.39	197.03	282.30	160.73	163.24	135.69	96.02
	103.30	230.85	311.70	171.40	186.65	141.27	82.41
	102.35	167.96	311.79	191.35	173.17	151.17	96.49
	93.50	200.45	283.76	171.67	151.61	140.32	106.16
Figure 6J p-p65							
	Con	1/4	1	3	7	14	21
Test for normal distribution							
Shapiro-Wilk test							
W	0.8319	0.9172	0.8083	0.8717	0.9808	0.9677	0.9082
P value	0.1438	0.5123	0.0945	0.2734	0.939	0.8603	0.4566
Passed normality test (alpha=0.05)?	Yes	Yes	Yes	Yes	Yes	Yes	Yes
P value summary	ns	ns	ns	ns	ns	ns	ns
Number of values	5	5	5	5	5	5	5
**Data exhibits a normal distribution**							
ANOVA summary							
F	143.8						
P value	<0.0001						
P value summary	****						
Significant diff. among means (P < 0.05)?	Yes						
R square	0.9686						
Dunnett's multiple comparisons test	Mean Diff.	95.00% CI of diff.	Significant?	Summary	Adjusted P Value		
Con vs. 1/4	-98.6	-120.7 to -76.46	Yes	****	<0.0001		
Con vs. 1	-198.7	-220.8 to -176.5	Yes	****	<0.0001		
Con vs. 3	-72.73	-94.86 to -50.60	Yes	****	<0.0001		

Con vs. 7	-68.01	-90.14 to -45.88	Yes	****	<0.0001		
Con vs. 14	-43.09	-65.23 to -20.96	Yes	****	<0.0001		
Con vs. 21	3.058	-19.07 to 25.19	No	ns	0.9977		

Figure 6K

Western blot	Con	1/4	1	3	7	14	21
	99.10	411.90	346.14	275.94	226.49	125.79	100.96
	93.25	311.61	321.53	292.01	151.11	153.98	106.04
	108.58	408.22	316.24	217.38	218.21	120.60	111.23
	103.35	392.66	346.48	238.13	162.85	132.45	143.01
	95.71	374.95	338.62	266.43	225.08	145.47	92.89
Figure 6K p-p65							
	Con	1/4	1	3	7	14	21
Test for normal distribution							
Shapiro-Wilk test							
W	0.9677	0.8354	0.8526	0.9595	0.7883	0.9467	0.8667
P value	0.8604	0.1527	0.203	0.8048	0.0648	0.7135	0.2534
Passed normality test (alpha=0.05)?	Yes	Yes	Yes	Yes	Yes	Yes	Yes
P value summary	ns	ns	ns	ns	ns	ns	ns
Number of values	5	5	5	5	5	5	5
**Data exhibits a normal distribution**							
ANOVA summary							
F	91.44						
P value	<0.0001						
P value summary	****						
Significant diff. among means (P < 0.05)?	Yes						
R square	0.9514						
Dunnett's multiple comparisons test	Mean Diff.	95.00% CI of diff.	Significant?	Summary	Adjusted P Value		
Con vs. 1/4	-279.9	-324.7 to	Yes	****	<0.0001		

		-235.1					
Con vs. 1	-233.8	-278.6 to -189.0	Yes	****	<0.0001		
Con vs. 3	-158	-202.8 to -113.2	Yes	****	<0.0001		
Con vs. 7	-96.75	-141.6 to -51.94	Yes	****	<0.0001		
Con vs. 14	-35.66	-80.47 to 9.151	No	ns	0.1588		
Con vs. 21	-10.83	-55.64 to 33.98	No	ns	0.9652		
